# Reduction of Urogenital Schistosomiasis with an Integrated Control Project in Sudan

**DOI:** 10.1371/journal.pntd.0003423

**Published:** 2015-01-08

**Authors:** Young-Ha Lee, Hoo Gn Jeong, Woo Hyun Kong, Soon-Hyung Lee, Han-Ik Cho, Hae-Sung Nam, Hassan Ahmed Hassan Ahmed Ismail, Gibril Nouman Abd Alla, Chung Hyeon Oh, Sung-Tae Hong

**Affiliations:** 1 Department of Infection Biology, Chungnam National University School of Medicine, Daejeon, Korea; 2 Korea Association of Health Promotion, Seoul, Korea; 3 Department of Preventive Medicine, Chungnam National University School of Medicine, Daejeon, Korea; 4 National Control Program for Schistosomiasis and Soil-Transmitted Helminthes, Federal Ministry of Health, Sudan; 5 Korea International Cooperation Agency, Seongnam, Korea; 6 Department of Parasitology and Tropical Medicine, Seoul National University College of Medicine, Seoul, Korea; University of Queensland, Australia

## Abstract

**Purpose:**

Schistosomiasis remains a major public health concern in Sudan, particularly *Schistosoma haematobium* infection. This study presents the disease-reduction outcomes of an integrated control program for schistosomiasis in Al Jabalain locality of White Nile State, Sudan from 2009 through 2011.

**Methods:**

The total population of the project sites was 482,902, and the major target group for intervention among them was 78,615 primary school students. For the cross-sectional study of the prevalence, urine and stool specimens were examined using the urine sedimentation method and the Kato cellophane thick smear method, respectively. To assess the impacts of health education for students and a drinking water supply facility at Al Hidaib village, questionnaire survey was done.

**Results:**

The overall prevalence for *S. haematobium* and *S. mansoni* at baseline was 28.5% and 0.4%, respectively. At follow-up survey after 6–9 months post-treatment, the prevalence of *S. haematobium* infection was reduced to 13.5% (95% CI = 0.331–0.462). A higher reduction in prevalence was observed among girls, those with moderately infected status (around 20%), and residents in rural areas, than among boys, those with high prevalence (>40%), and residents in urban areas. After health education, increased awareness about schistosomiasis was checked by questionnaire survey. Also, a drinking water facility was constructed at Al Hidaib village, where infection rate was reduced more compared to that in a neighboring village within the same unit. However, we found no significant change in the prevalence of *S. mansoni* infection between baseline and follow-up survey (95% CI = 0.933–6.891).

**Conclusions:**

At the end of the project, the prevalence of *S. haematobium* infection was reduced by more than 50% in comparison with the baseline rate. Approximately 200,000 subjects had received either praziquantel therapy, health education, or supply of clean water. To consolidate the achievements of this project, the integrated intervention should be adapted continuously.

## Author Summary

Schistosomiasis remains a major public health concern and is one of the major causes of morbidity among school-aged children in Sudan. To control schistosomiasis in White Nile State of Sudan, the Korea International Cooperation Agency (KOICA) implemented an integrated control program including mass chemotherapy with praziquantel and health education to school children and village residents, and construction of a drinking water supply facility at Al Hidaib village from 2009 to 2011. As a result of this project, the overall prevalence of *S. haematobium* infection was reduced by >50% in comparison with the baseline rates (95% CI = 0.331–0.462). The infection reduction rates were higher among girls, those with moderate infection status (around 20%), and residents of rural areas; than for those of boys, subjects with high infection status (>40%) and residents of urban areas. A supply of clean water at Al Hidaib village contributed significantly to the reduction in the prevalence of urogenital schistosomiasis in comparison to Khour Ajwal village, which is similar natural environment and lifestyle of Al Hidaib village. However, the prevalence of *S. mansoni* infection did not significantly change. Also, the awareness of knowledge about schistosomiasis and health improvement was apparently improved by the results of questionnaires survey. Through this project, approximately 200,000 individuals benefited from either drug treatment, health education, or a clean water supply. To consolidate the achievements of the project, sustainable integrated control activities should be implemented in the near future.

## Introduction

Schistosomiasis is a parasitic trematodiasis caused by several species of the genus *Schistosoma*, of which *S. mansoni, S. japonicum*, *S. mekongi*, and *S. haematobium* are of public health importance. These worms live in the veins around the intestine or urinary bladder. Eggs are released in the stool or urine of the host and hatch in water [1]. Humans are usually infected when they come into contact with contaminated fresh water such as collecting water, washing, bathing, playing, fishing, or cultivating crops. In general, children, women, fishermen, and farmers are the high risk groups in schistosomiasis, also other people can infect in the irrigation channels or rivers and suffer from hematuria and anemia, enlargement of the liver and spleen, and growth retardation [1], [2].

In 2012, schistosomiasis was considered endemic in 78 countries [3]. In sub-Saharan Africa, 70 million individuals were estimated to have experienced hematuria in the previous 2 weeks, 32 million had dysuria associated with *S. haematobium*, 18 million had major bladder wall pathology, and 10 million had *S. haematobium*-related major hydronephrosis, resulting in an estimated mortality of 150,000 people per year [4]. In 2012, the World Health Organization (WHO) estimated that approximately 249 million people required preventive chemotherapy annually; this figure includes 114 million school-age children, 231 million of whom were in Africa. However, only approximately 42 million people, merely 14.4% of the infected population, received schistosomiasis treatment [3]. Sudan is one of the endemically infected sub-Saharan countries in which both the urogenital and intestinal forms of schistosomiasis are prevalent in populations along the Nile River [5]. *S. haematobium* infection is dominant and the prevalences range from 1.7% to 56.0% in different localities, whereas the prevalence for *S. mansoni* is very low [6]–[10].

The Blue and White Nile Rivers meet in Sudan to form the Nile River. Among the countries crossing the Nile River, Sudan has the widest river basin areas, and it also has large irrigated agricultural sectors along the banks of the Nile River. The development of such irrigation schemes has led to significant environmental modification, favoring the spread of vector-borne diseases, including schistosomiasis [11]. Residents of these geographical environments along the Nile River have faced the risk of schistosomiasis for many centuries.

During 1979–1990, the Blue Nile Health Project was implemented as a comprehensive plan to control malaria, schistosomiasis, and diarrheal diseases at Gezira, Managil, and Rahad irrigation schemes. As a result, the prevalence of schistosomiasis was reduced from 53% to less than 10% [10]. However, there have been no further integrated programs to control schistosomiasis in Sudan thereafter. In 2000, the Sudanese government established a national schistosomiasis control program to reduce the prevalence of *S. haematobium* to less than 10% by 2013. As part of this program, the Korea International Cooperation Agency (KOICA) supported a schistosomiasis control project at the Al Jabalain locality in the White Nile State of Sudan during 2009–2011. The KOICA project included a mass chemotherapy according to baseline survey, health education with material development, construction of a drinking water supply facility at high endemic village, provision of laboratory equipment, dispatch of Korean experts to Sudan and follow-up evaluation.

The present study focused on the outcomes of praziquantel administraion and health education for students and residents, as well as the impacts of a filtered drinking water supply facility at Al Hidaib village among integrated control project supported by the KOICA.

## Methods

### Overview of the KOICA Project

An agreement (Project Code No. 2008-01-0000-039) to conduct “The Project for Combating Schistosomiasis in Sudan" was officially signed by the KOICA of the Republic of Korea and the Federal Ministry of Health (MOH) of the Republic of Sudan in Khartoum, Sudan in June, 2009. According to the signed agreement, the KOICA provided full funding for this project as an official development aid (ODA) program for developing countries. The project was carried out between August 2009 and June 2011 by the Korea Association of Health Promotion (KAHP), and the KAHP provided management consultation for the project. The pilot area of this project was Al Jabalain locality of White Nile State, Sudan, which is composed of 6 units. In Sudan, the unit is a last step of local administrative organization, thus we used the unit as a basic structure of implementation activities. This site was recommended by Sudanese government, because the prevalence of *S. haematobium* infection of Al Jabalain locality was so high according to the results surveyed by White Nile State MOH, Sudan (25.5%–54.1%).

**Figure 1:**
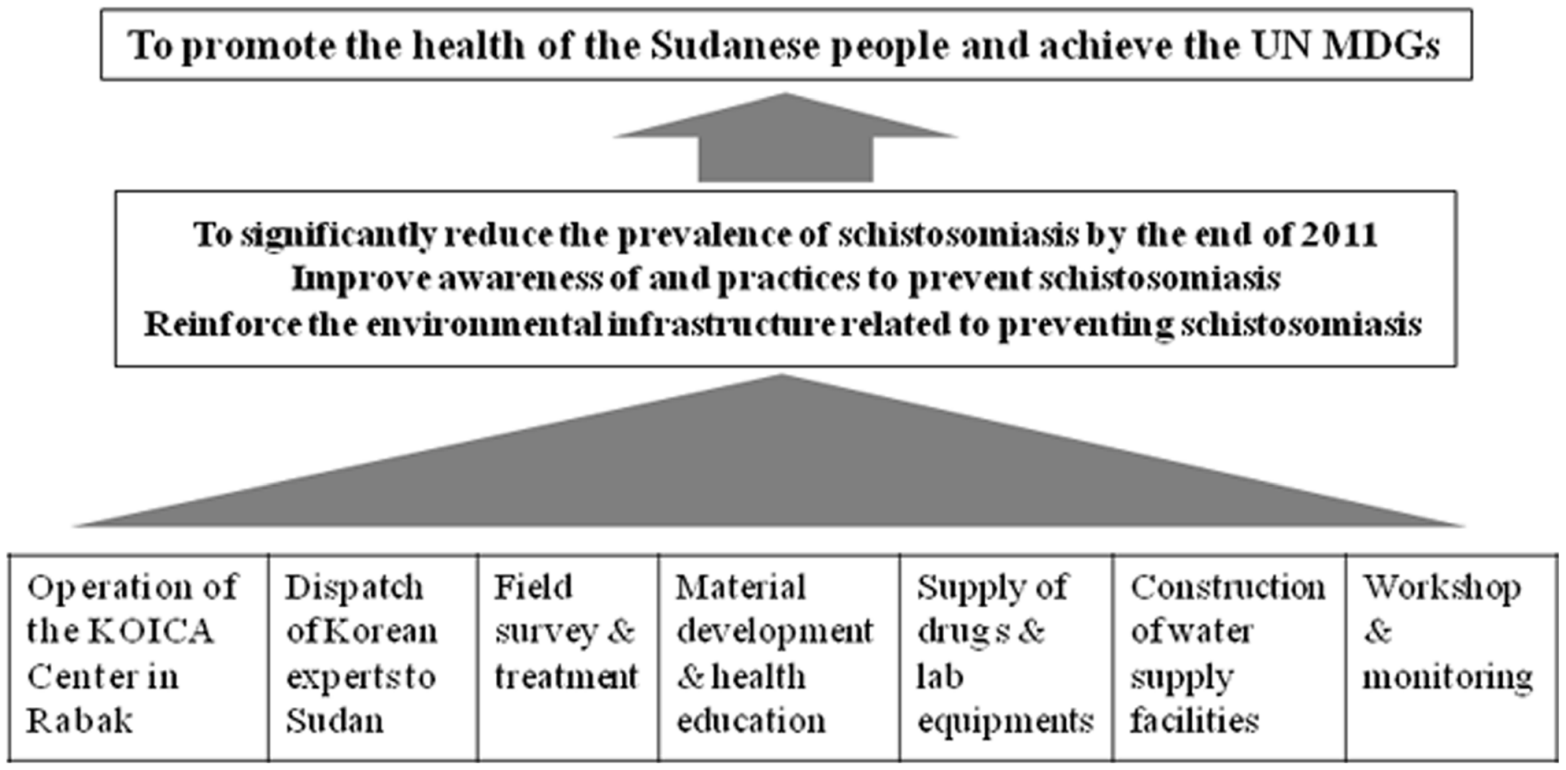
Flow diagram of "The Project for Combating Schistosomiasis in Sudan 2009–2011." KOICA, Korea International Cooperation Agency. MDG; Millennium Development Goals established following the Millennium Summit of the United Nations in 2000.

As shown in Fig. 1, the KOICA project included many activities; however, this study focused on the mass chemotherapy with baseline survey, the health education for primary school students and village residents, and the construction of a facility at Al Hidaib to supply drinking water. These 3 interventions were explained in detail below.

### Ethics statement

This study protocol was reviewed and approved by the institutional review board of KAHP, which was the implementing organization of this project (Approval No. 10-C-05). Data were collected at pilot areas according to the board-approved framework. Before conducting the survey at each school, informed verbal consent was first obtained from the chief of the villages and the parents of children during the village meeting and was also obtained from school teachers at schools prior to the recruitment of children. In villages, informed verbal consent was first obtained from the village leaders and/or the parents of children during a village meeting. As per traditional culture and due to the low literacy rates in village residents, verbal consent was deemed acceptable. The research program was approved by MOH, White Nile State, Sudan.

### Organization of KOICA Team for Implementation of the Project in Sudan

The implementation team for this project was composed of one Korean project manager (PM) in Sudan (team leader), one Sudanese PM, 2 Sudanese parasitology experts, 4 Sudanese laboratory technicians, 7 Sudanese health workers and 2 Sudanese drivers.

The Korean PM, who had stayed in Sudan during the project period, was an expert of parasitology and health administration. He managed the whole activities implemented in Sudan. The Sudanese PM and senior parasitology staff were public health experts, who worked more than 15 years at schistosomiasis control programs in Sudan. The Sudanese experts were responsible for the whole activities with the Korean PM, and implemented the health education, communication between Korean PM and Sudanese staff, contact with Sudanese administration officials and residents, drug delivery, and public relationships. Laboratory technicians have worked more than 5 years at parasitological examination using microscope, and worked as officials of MOH, White Nile State. They were responsible for performing the parasitological examination of urine and stools, and recording the results. Health workers were also officers of MOH, White Nile State, and learned about schistosomiasis control and information related with this project from Korean and Sudanese PM or parasitologists sufficiently. They were responsible for collecting the samples, questionnaire survey, assistance of health education and drug delivery.

In addition, two Korean parasitology experts and one administration officer visited project area in Sudan 5 times every 4–6 months for 2 weeks each time during the project periods. They managed the project and transferred the basic concept on parasite control. Also they monitored and evaluated the project progress with Sudanese experts and resolved the problems issued at implementation activities. Korean and Sudanese PM or parasitology experts provided training to health workers and laboratory technicians about the project implementation activities in case of necessary, from time to time, and individually or collectively.

### Project sites and their population

**Figure 2:**
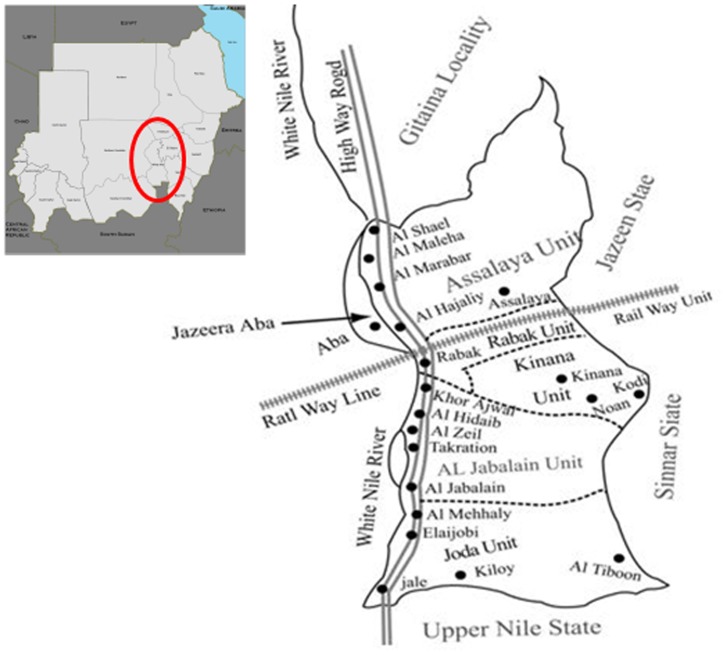
Location of the Al Jabalain locality, the site of "The Project for Combating Schistosomiasis in Sudan 2009–2011". The Al Jabalain locality is situated on the east bank of the White Nile River in southern White Nile State. Dot (•); baseline survey areas of each unit in the Al Jabalain locality of White Nile State, Sudan.

**Figure 3:**
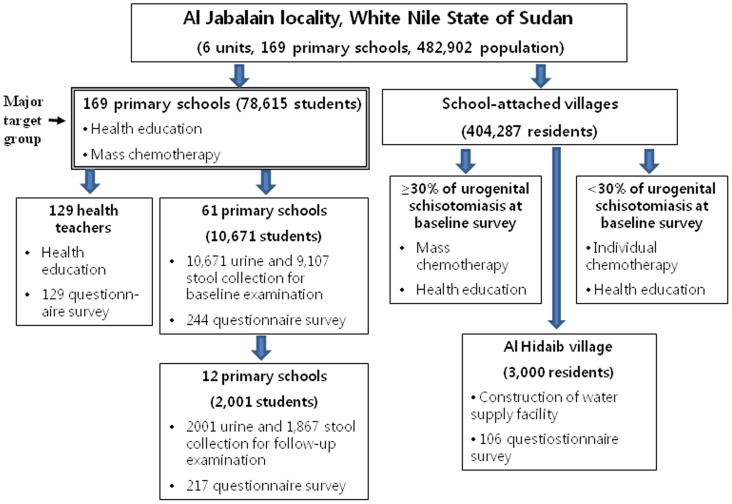
Flow chart of the participation and adherence in the present study. This project was carried out at Al Jabalain locality of White Nile State in Sudan from August 2009 to June 2011. The study areas included a total of 169 primary schools and 482,902 individuals, including 78,615 primary school students. For baseline survey, 10,671 urines and 9,107 stools were collected from 10,671 students of 61 primary schools before treatment. For follow-up evaluation, 12 schools were randomly selected among 61 schools for baseline survey, and 2,001 urines and 1,867 stools were collected from 2,001 students. According to the results of baseline survey of primary school belonging the village, village residents were received the different patterns of chemotherapy and health education.

The project site was Al Jabalain locality of White Nile State, Sudan (Fig. 2), which is composed of six units. We used the unit as a basic structure of implementation activities in Sudan. White Nile State is located along the White Nile River in the southeastern part of Sudan, and water canals are well developed in this region. As the land is almost flat, irrigation is based on a network of gravity-fed mud-lined canals. The Al Jabalain locality is situated on the east bank of the White Nile River in southern White Nile State. All villages included in the study were located adjacent to the White Nile River, and most houses were built with mud bricks. Most village residents used water from the river and earned their income from agriculture, livestock breeding, or fishing. The study areas included a total of 169 primary schools and 482,902 individuals, including 78,615 primary school students (Table 1). Rabak unit was an urbanized community, whereas the other Al Jabalain, Assalaya, Jazeera Aba, Joda and Kenana units were rural communities. Fig. 3 shows the adherence of participants to the present study.

**Table 1:** Number of students and village residents in the Al Jabalain locality of White Nile State, Sudan.

Units	No. of schools	No. of students	No. of village residents	Population by age group		
				Under 15 years old	Over 15 years old	Total*
Al Jabalain	30	10,226	58,745	30,347	38,624	68,971
Assalaya	29	10,444	59,101	30,600	38,945	69,545
Jazeera Aba	18	6,474	36,639	18,970	24,143	43,113
Joda	10	4,463	21,703	11,513	14,653	26,166
Kenana	34	17,750	91,975	48,279	61,446	109,725
Rabak	48	29,258	136,124	72,768	92,614	165,382
Total	169	78,615	404,287	212,477	270,425	482,902

*Total population  =  No. of students + No. of village residents.

### Sample size calculation and selection for baseline survey

According to the agreement, the total numbers for parasitological examination were approximately 10,000 among 78,615 students, and they were selected as follows. The numbers of total students (78,615) were approximately 7.8 times more than target number (approximately 10,000) for baseline survey. Assigned numbers of students of each unit were calculated by dividing of total student numbers of each unit by 7.8. Numbers of surveyed schools were calculated by dividing half of mean number of students of each school in each unit, because samples from the 1^st^, 3^rd^ and 5^th^ grade classes of each school were collected. Since there were some differences of students' number of each school, we substantially determined the number of schools to collect samples at each unit by a number of averages ± 20%.

**Figure 4:**
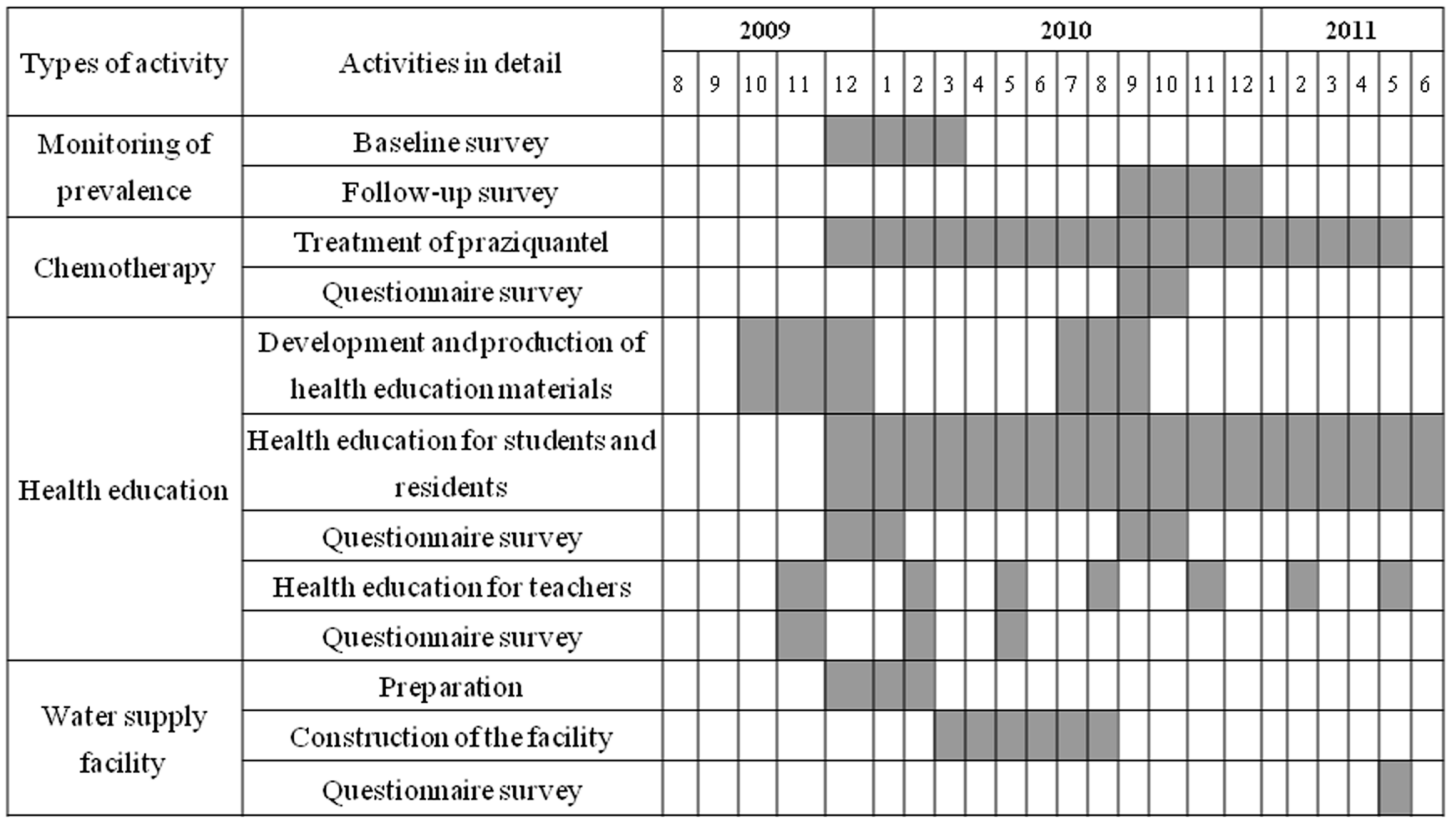
Implementation schedules of chemotherapy, health education, and construction of water supply facility. To evaluate the prevalence of *S. haematobium* and *S. mansoni* infection, baseline survey was implemented at 61 primary schools in Al Jabalain locality before treatment, and the follow-up survey was conducted at 6–9 months after the chemotherapy. Chemotherapy and health education for students and residents were carried out through the whole project period. A drinking water supply facility at the Al Hidaib village was completed at August, 2010, and questionnaire survey from residents was done 9 months after completion.

The number of schools surveying at baseline were finally selected considering the location, total number of students, accessibility to school, and environmental condition of schools. To evaluate the prevalence of *S. haematobium* and *S. mansoni* infection, baseline survey was implemented at 61 primary schools in Al Jabalain locality from December 2009 to March 201 (Table 2, Fig. 4). In one school, we collected the samples around 80-250, but not more than 300. If the number of students of one school was less than 100, all students were subjected. The STROBE check is available as Supporting Information (S1 Checklist).

**Table 2:** The prevalences of *S*. *haematobium* and *S. mansoni* infection of primary school children at the baseline survey.

Units*	No. of schools	Urine for *S. haematobium* examination		Stool for *S. mansoni* examination	
		No. of samples	No. of positive (%)	No. of samples	No. of positive (%)
Al Jabalain	9	1,617	347 (21.5)	1,449	3 (0.2)
Assalaya	10	1,526	215 (14.1)	1,218	60 (4.9)
Jazeera Aba	7	914	66 (7.2)	711	1 (0.1)
Joda	3	443	178 (40.2)	321	0 (0)
Kenana	12	2,307	430 (18.6)	1,940	0 (0)
Rabak	20	3,864	237 (6.1)	3,468	28 (0.8)
Total	61	10,671	1,473 (13.8)	9,107	92 (1.0)

*Rabak unit is urban community, whereas the other 5 units are rural communities.

### Collection of urine and stool samples

For evaluation of prevalence and intensity of schistosomiasis of the pilot areas, urine and fecal samples were collected from 1^st^, 3^rd^ and 5^th^ graders of the sentinel schools of project area. The sample collection team was composed of Korean PM in Sudan, Sudanese PM and Parasitology experts for health education, health workers, and driver. Health workers were responsible for sample collection basically, and receiving the oral consent from each school child.

Containers were delivered in the morning, and then stool and urine samples were collected at the same day (usually within 6 hours, or around 2–3 pm before leaving school), where the teachers helped to check who submitted the samples or not among enrolled students. After delivery the containers to students, Sudanese PM or experts educated students in the classes or ground of the school about prevention of schistosomiasis and public health. The collected samples were transferred to the Schistosomiasis Control Center established by the KOICA project in Rabak, White Nile State immediately.

### Parasitological examination

The prevalence and infection intensity of *S. haematobium* and *S. mansoni* infection in students were examined at baseline and follow-up surveys. The samples were basically examined on the day or next day after collection.

Four laboratory technicians examined the samples parasitogically. They have career more than 5 years about urine and stool examination. For quality control, Sudanese or Korean PM or parasitology experts reexamined about 10% of the slides and confirmed the first results. In case of disagreement, the results were discussed with the concerned technician and re-read the discordant slides until agreement was reached. To increase the capability of technicians, Korean experts educated all laboratory technicians about microscope manipulation, identification of parasites, urine and stool processing methods repeatedly.

For the diagnosis of *S. haematobium*, urine samples were subjected to a urine sedimentation method, as described elsewhere [12]. Briefly, the amount of urine of each tube was adjusted into 10 mL. Urine samples (10 mL) were centrifuged at 1,500 rpm for 5 minutes at room temperature, and pellets were vigorously shaken and examined for *S. haematobium* or *S. mansoni* eggs by microscopy. To examine the whole pellets of the tube as possible, all sediments of each tube were transferred into 2–4 slide glasses. The presence of *S. haematobium* or *S. mansoni* eggs of each child was the results of a total of 2–4 smears.

For the diagnosis of *S. mansoni*, fecal samples were subjected to the Kato cellophane thick smear method [13]. Briefly, 1 g of fecal sample was obtained from each tube and prepared two smears using slide glasses, and allowed to clear for at least 30 minutes before examination. The presence of *S. mansoni* eggs of each child was the sum of 2 smears.

### Mass drug administration

Four medical doctors participated in the KOICA project, namely, two Sudanese and two Korean parasitology experts. All drug treatment was under control of these doctors. Also Sudanese PM and health workers had much experience participating schistosomiasis control projects in Sudan before. The Korean and Sudanese PM received full education about chemotherapy many times. The treatment team included some or all of the followings: Korean PM in Sudan, Sudanese PM or parasitology expert, health workers for assistance of health education, reception and medication, and driver. Health workers delivered the drug actually under the control of medical doctors. All health workers are White Nile MOH officials, and they were educated drug treatment and schistosomiasis control from Sudanese and Korean parasitology experts. Within each school or village, treatment was provided on the basis of the baseline prevalence of schistosomiasis in sentinel primary schools.

The strategy for controlling morbidity due to schistosomiasis was based on chemotherapy using praziquantel. The chemotherapy strategy followed the schistosomiasis control protocol of Sudanese government, which was a modified treatment strategy of WHO recommendation [14]. In this project, the major target group was the primary school students. We treated all primary school children in Al Jabalain locality at the 1st year of the project regardless to the levels of infection. At the second year, mass chemotherapy was implemented in schools with more than 10% of urogenital schistosomiasis at baseline survey. The WHO dose pole was used to determine dosage (40 mg/kg) for school children [14]. In villages, mass treatment was conducted where the prevalence of urogenital schistosomiasis was more than 30% at baseline.

As shown in time bar of mass chemotherapy, drug administration was done over the year (Fig. 4). During treatment period, health workers went to schools in the morning, and they administered praziquantel for treatment of school children directly. If children have been registered but were absent on the day of treatment, the health workers visited again and treated them as long as it was within the treatment period. If they were absent because they were sick, the health workers should have waited until they got better.

In villages, residents were treated by health workers with assistance of village leaders. Treatment could be completed in a few days, but should take no longer than 1 week for schools or villages.

### Health education and/or questionnaire survey

The total population of the pilot area was the subjects of health education (482,902), however the major target population was 78,615 primary school students. The health education team included one Korean PM in Sudan, one Sudanese PM or Parasitology experts, two health workers for assistance of health education, two health workers for questionnaire survey (not always) and one driver. Basically, Sudanese PM or parasitology expert educated teachers, students, and village residents about schistosomiasis and health improvement. However, trained health workers taught students when multiple programs of health education were held at the same time.

Korean and Sudanese PM, and Korean experts developed the materials for health education such as presentation file, leaflets, posters, and brochures to provide knowledge related to schistosomiasis and health improvement. The developed materials were used to educate school students and teachers as well as village residents. Also they developed the "Manual for Schistosomiasis Control" to standardize the schistosomiasis control activities and to improve the capacity building of Sudanese staffs participating this project (S1 Appendix).

For health education of school teachers, teachers were gathered at one school or community education center of each unit, and Sudanese PM or parasitology experts educated them about schistosomiasis and health improvements using beam project. Though this training, school teachers would be health education experts who can deliver sufficient knowledge of schistosomiasis to students. School teachers received the certificate of completeness of health education, and the education took 5 hours totally (S1 Video).

For education of school students and village residents, Sudanese PM or parasitology experts educated them in the project areas to provide sufficient knowledge on schistosomiasis and promote self-care health awareness for prevention of re-infection. Sudanese PM or parasitology experts educated the school children or village residents using education materials at the playground or classroom in the school for 1 hour, and sometimes beam projector was used for health education (S2 Video). In some cases, health education was combined with administration of praziquantel or questionnaire survey.

Every year, KOICA team planned the health education schedules for students through the attending school period. Basically, the team visited all primary schools at least one time during the project period. The schools of high prevalence (more than 30%) tried to visit 1–2 times per year for 2 years. Village residents were invited to school playground in case of more than 30% of prevalence of the sentinel school. School teachers were educated one time per year for 2 years (Fig. 4).

To evaluate the effects of health education, the semi-structured questionnaire survey was conducted at baseline and follow-up survey, which was consisted of information related to schistosomiasis. Teachers or Sudanese health workers thoroughly explained the questions to the students and obtained the consents, and then received the answer sheets.

### Construction of a drinking water supply facility

Since schistosomiasis is one of the water-transmitted helminthiases, the supply of safe and clean water is very important for prevention of infection. This project included one large scale filtered drinking water supply facility at a high endemic village in the pilot area. We suggested selection criteria to determine the construction site; high prevalence of schistosomiasis, availability of drinking water supply facility, and feasibility of construction. According to the selection criteria, one drinking water supply facility was constructed at Al Hidaib village in Al Jabalain unit using the water from the main stream of the White Nile River.

**Figure 5:**
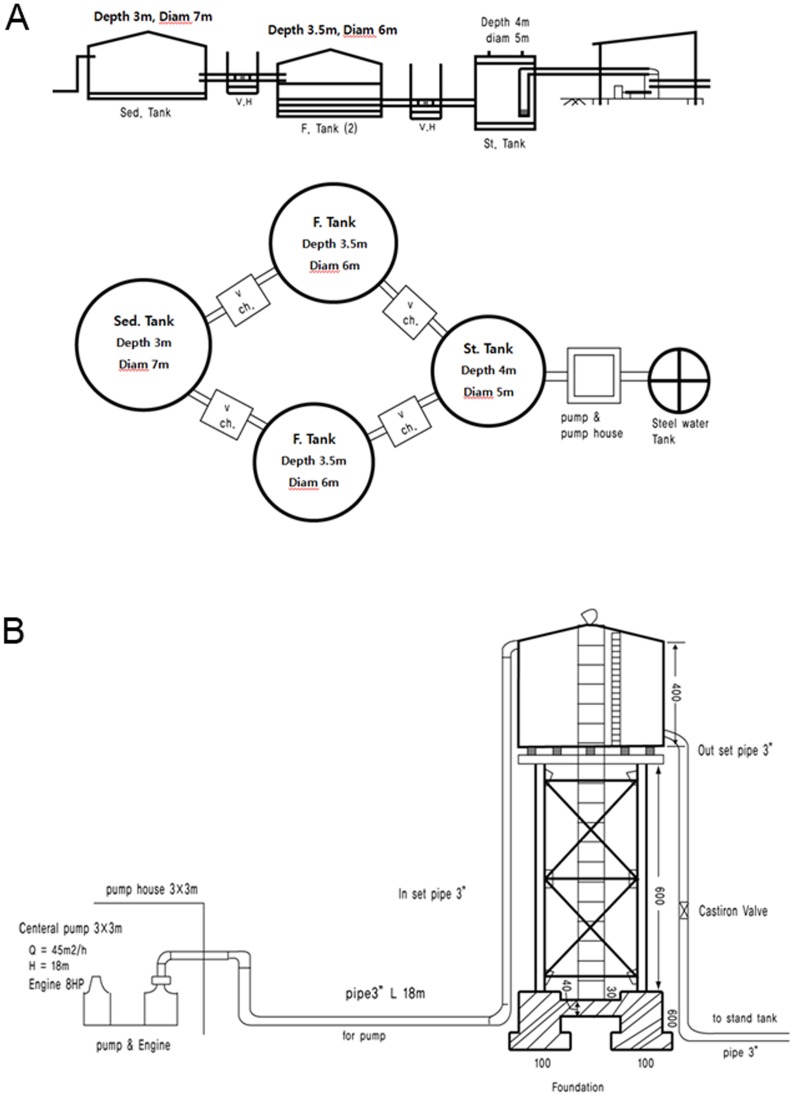
Blueprint of a filtered drinking water supply facility. Slow sand filtration system (A) and water supply system (B). A drinking water supply facility was constructed at the Al Hidaib village through this project. About 2000 persons were using the water supply facility every day. Sed. Tank, sedimentation tank; Ch. V, Check valves in the pipes; F. tank, filtration tank; St. tank, storage tank. Diam, diameter.

The facility was constructed for 6 months from March to August 2010 (Fig. 4). It installed a slow sand filtration system using pipelines, pumps, and tanks for sedimentation, filtration and storage (Fig. 5). The gravel, sand and charcoals as a filter should be cleaned about 4 times annually depending on the degree of contamination, and maintenance manual should be prepared and be kept near the facility. The water storage capacity was up to 59,290 L/day, and 3,000 residents were lived in Al Hidaib village in Al Jabalain unit.

### Follow-up evaluation

After starting the implementation of this project, the impacts and results of activities were monitored regularly by the project team itself or specific teams. During the follow-up evaluation, we conducted the parasitology examination, questionnaire survey from school students about health education, questionnaires survey from residents about the drinking water supply facility, questionnaires survey from school students about drug administration, and questionnaire survey from teachers about health education.

The evaluation team was composed of Korean and Sudanese PM or parasitology experts, Sudanese Federal and White Nile State MOH officials, and KOICA staffs. The schools for follow-up evaluation were basically selected by randomization procedure as follows. Simple random sampling was used to select primary schools per unit using the lottery method [15]. This involved the listing names of all baseline-surveyed primary schools in each unit on small slips of paper, then after a thorough mixing of the names, 1–3 schools were selected one by one. In one school, we collected the urine and stool samples around 80–250 from the 1st, 3rd and 5th graders, but not more than 300. If the sample size of one school was less than 100, all students were collected. Al Hidaib village in Al Jabalain unit was selected to evaluate the effects of drinking water supply facility from residents. The follow-up survey for evaluation of the prevalence of *S. haematobium* and *S. mansoni* infection was carried out 12 primary schools in Al Jabalain locality from September to December 2010 (Tables 3 and 4, Fig. 4).

**Table 3:** The prevalence of *S. haematobium* infection of selected primary school children before and after administration of praziquantel.

Units	Schools	Baseline survey		Follow-up survey*		OR	95% CI	*P*-value
		Exam. No	Positive No. (%)	Exam. No	Positive No. (%)			
Al Jabalain	Al Hidaib	89	41 (46.1)	161	33 (20.5)	0.302	0.171–0.531	0.000
	Khour Ajwal	77	21 (27.3)	169	77 (45.6)^†^	2.232	1.242–4.009	0.007
	Subtotal	166	62 (37.3)	330	110 (33.3)	0.839	0.568–1.237	0.375
Assalaya	Assalaya East	103	23 (22.3)	291	13 (4.5)	0.163	0.079–0.336	0.000
Jazeera Aba	Tayba boys	118	25 (21.2)	150	4 (2.7)	0.102	0.034–0.302	0.000
	Tayba girls	100	13 (13.0)	147	4 (2.7)	0.187	0.059–0.592	0.004
	Subtotal	218	38 (17.4)	297	8 (2.7)	0.131	0.060–0.287	0.000
Joda	Al Barra Ebn Malik	173	55 (31.8)	158	12 (7.6)	0.176	0.090–0.345	0.000
	Abu Baker Alsiddig	150	83 (55.3)	150	28 (18.7)	0.185	0.110–0.312	0.000
	Subtotal	323	138 (42.7)	308	40 (13.0)	0.200	0.134–0.298	0.000
Kenana	Al Farook boys	270	65 (24.1)	152	24 (15.8)	0.591	0.352–0.992	0.047
	Abd Rahman Ebnouf mixed	231	85 (36.8)	150	33 (22.0)	0.484	0.303–0.775	0.003
	Subtotal	501	150 (29.9)	302	57 (18.9)	0.544	0.385–0.769	0.000
Rabak	Abu Tileih boys	103	37 (35.9)	165	17 (10.3)	0.205	0.108–0.390	0.001
	Al Khansa girls	81	4 (4.9)	155	4 (2.6)	0.510	0.124–2.095	0.350
	Al Wifak boys	193	29 (15.0)	153	21 (13.7)	0.900	0.491–1.650	0.733
	Subtotal	377	70 (18.6)	473	42 (8.9)	0.427	0.284–0.644	0.000
Total	Boys	1,436	440 (30.6)	1,599	245 (15.3)	0.367	0.305–0.442	0.000
	Girls	252	41 (16.3)	402	25 (6.2)	0.537	0.363–0.795	0.002
	Total	1,688	481 (28.5)	2,001	270 (13.5)	0.391	0.331–0.462	0.000

* The follow-up survey was conducted at 6-9 months after the chemotherapy.

† The egg positive rate of schistosomiasis was increased from 27.3% to 45.6% at Khour Ajwal school of Al Jabalain Unit. OR, odd ratio; 95% CI, 95% confidence intervals

**Table 4:** The prevalence of *S. mansoni* infection of selected primary school children before and after administration of praziquantel.

Units	Schools	Baseline survey		Follow-up survey*		OR	95% CI	*P*-value
		Exam. No	Positive No. (%)	Exam. No	Positive No. (%)			
Al Jabalain	Al Hidaib	90	2 (2.2)	147	0 (0.0)	-	-	-
	Khour Ajwal	91	1 (1.1)	153	0 (0.0)	-	-	-
	Subtotal	181	3 (1.7)	300	0 (0.0)	-	-	-
Assalaya	Assalaya East	109	0 (0.0)	285	6 (2.1)	-	-	-
Jazeera Aba	Tayba boys	97	1 (1.0)	140	4 (2.9)	2.824	0.311–25.657	0.357
	Tayba girls	93	0 (0.0)	142	2 (1.4)	-	-	-
	Subtotal	190	1 (0.5)	282	6 (2.1)	4.109	0.491–34.404	0.192
Joda	Al Barra Ebn Malik	128	0 (0.0)	144	0 (0.0)	-	-	-
	Abu Baker Alsiddig	87	0 (0.0)	149	0 (0.0)	-	-	-
	Subtotal	215	0 (0.0)	293	0 (0.0)	-	-	-
Kenana	Al Farook boys	148	0 (0.0)	109	0 (0.0)	-	-	-
	Abd RahmanEbnouf mixed	198	0 (0.0)	152	1 (0.7)	-	-	-
	Subtotal	346	0 (0.0)	261	1 (0.4)	-	-	-
Rabak	Abu Tileih boys	99	1 (1.0)	158	3 (1.9)	1.897	0.195–18.493	0.582
	Al Khansa girls	65	0 (0.0)	150	1 (0.7)	-	-	-
	Al Wifak boys	180	0 (0.0)	138	0 (0.0)	-	-	-
	Subtotal	344	1 (0.3)	446	4 (0.9)	3.104	0.345–27.898	0.312
Total	Boys	1,195	5 (0.4)	1,575	14 (0.9)	2.110	0.660–6.745	0.208
	Girls	190	0 (0.0)	292	3 (1.0)	3.023	0.369–24.738	0.302
	Total	1,385	5 (0.4)	1,867	17 (0.9)	2.536	0.933–6.891	0.068

* The follow-up survey was conducted at 6–9 months after the chemotherapy.

They visited the selected schools and villages to measure the project indicators. The major indicators were the prevalence of *S. haematobium* infection, number of students and village residents participating in a health education activity (at least once), the percentage of participants with increased awareness of schistosomiasis after completing health education, number of village residents using the water supply facility, and its impacts.

### Statistical analysis

SPSS version 15.0 software (SPSS Inc., San Diego, CA, USA) was used to analyze the experimental data. The differences in prevalence of *S. haematobium* and *S. mansoni* infection and questionnaire survey about health education between baseline and follow-up were tested using the logistic regression analysis. Odds ratio (OR) and 95% confidence interval (CI) were calculated. Categorical variables were tested using the *χ* ^2^ test. A *P*-value of <0.05 was considered statistically significant.

## Results

### Baseline prevalence of schistosomiasis

For the baseline survey of schistosomiaiss in the Al Jabalain locality of White Nile State, we selected 61 primary schools according to the selection method (Fig. 3, Table 2). Three to twenty schools were selected each unit due to the total number of primary school students. A total of 10,671 urine samples and 9,107 stool samples were collected from students in grades 1, 3, and 5 at primary schools.

As a result of parasitological examination, the prevalence rates for *S. haematobium* and *S. mansoni* were 13.8% (1,473 of 10,671 cases) and 1.0% (92 of 9,107 cases), respectively. The prevalence of *S. haematobium* varied from 6.1% to 40.2% and *S. mansoni* 0% to 4.9% by unit of the Al Jabalain locality. The prevalence rate of *S. haematobium* was high at Joda (40.2%), Al Jabalain (21.5%), and Kenana (18.6%) units but low in Rabak (6.1%) and Jazeera Aba (7.2%) units. The *S. mansoni* prevalence rate was 0% at Joda and Kenana units, but 0.8% at Rabak unit and 4.9% at Assalaya unit.

### Post-intervention follow-up monitoring of schistosomiasis

In the 12 sentinel schools, the prevalence rate of *S. haematobium* and *S. mansoni* was 28.5% and 0.4% respectively at the baseline survey (Tables 3 and 4). Follow-up survey 6–9 months after the intervention detected 13.5% prevalence rate for *S. haematobium* and 0.9% for *S. mansoni*. The prevalence of *S. haematobium* infection after praziquantel treatment had decreased significantly in comparison to the baseline prevalence (OR  = 0.391, 95% CI  = 0.331–0.462, *P*<0.001). The *S. haematobium* infection rates in the Jazeera Aba (95% CI  = 0.060–0.287), Assalaya (95% CI  = 0.079–0.336), Joda (95% CI  = 0.134–0.298), Rabak (95% CI  = 0.284–0.644), and Kenana (95% CI  = 0.385–0.769) units were significantly reduced, but no significant change in the prevalence was observed in the Al Jabalain unit (95% CI  = 0.568–1.237).

**Figure 6:**
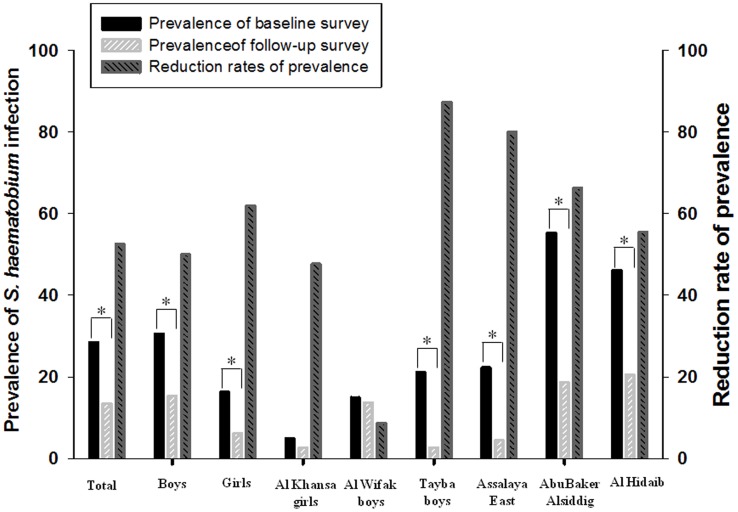
Reduction in the prevalence of *S. haematobium* infection between the baseline and follow-up surveys. The baseline survey was done at 61 primary schools in Al Jabalain locality before treatment, and the follow-up survey was conducted at 12 schools 6–9 months after the chemotherapy. The reduction rates of prevalence were calculated by [(baseline prevalence - follow-up prevalence)/baseline prevalence] ×100. The figure presented the *S. haematobium* infection rates of total population, total males and total females, and also showed the representative schools according to the reduction rates of prevalence (low, Al Khansa girls and Al Wifak boys; high, Tayba boys and Assalaya East; middle, AbuBaker Alsiddig and Al Hidaib). * *P*<0.001, significant difference in *S. haematobium* prevalence between baseline and follow-up surveys.

To characterize the reduction patterns of urinary schistosomiasis, the changes in prevalence according to school were analyzed. The prevalence was prominently reduced at 11 schools among 12 examined schools after implementation of the project, whereas the prevalence was significantly increased at Khour Ajwal unit at the follow-up survey (OR  = 2.232, 95% CI  = 1.242–4.009, *P* = 0.007). As shown in Fig. 6, reduction in the prevalence of *S. haematobium* infection was greater at schools with 20–25% baseline infection rates (Tayba boys and Assalaya East schools) than at those with <15% baseline infection rates (Al Khansa girls and Al Wifak boys schools) or >40% baseline infection rates (Abu Baker Alsiddig and Al Hidaib schools). The prevalence reduction of the other schools was between 34.4% and 79.1% than that of Khour Ajwal school. According to the sex, the reduction rate of *S. haematobium* prevalence was greater in girls (61.8%) than that in boys (50.0%).

The overall prevalence of *S. mansoni* infection was unchanged significantly (OR  = 2.536, 95% CI  = 0.933–6.891, *P = *0.068) (Table 4). The prevalence was not significantly changed by unit and sex.

### The effects of health education of school children

Korean and Sudanese PM or parasitology experts developed the materials for health education such as leaflets, billboard, brochure, poster, and power point files to provide knowledge related to schistosomiasis and health improvement. The developed materials were used to educate school students and teachers as well as village residents. Also they developed the manual for schistosomiasis control (see supporting information) to improve the capacity building of Sudanese staffs participating this project and educated them in case of necessary, from time to time, and individually or collectively, as well as standardized the schistosomiasis control activities.

Education about public health and schistosomiasis control was provided to enhance awareness and self-protection. In case of primary schools in the pilot area, health education team visited one time per year basically. The cumulative number of students who received health education for >1 hour was 121,418; thus, this number was estimated 1.5 times of all students in the pilot area, and the coverage rate of each unit during the whole project period was ranged from 131% to 175% of total number of students of each unit (Table 5).

**Table 5:** Cumulative number of participants attending health education at Al Jabalain locality of White Nile Sate, Sudan.

Unit	Primary school students			Primary school health teachers			Village residents		
	Total No.	No. of times	Cumulative No. (%)*	Total No.	No. of times	Cumulative No. (%)	Total No.	No. of times	Cumulative No. (%)
Al Jabalain	10,226	2.8	14,790 (145)	30	2	60 (200)	58,745	1.7	25,853 (44)
Assalaya	10,444	2.2	15,716 (150)	29	2	58 (200)	59,101	1.4	27,040 (46)
Jazeera Aba	6,474	2.1	10,002 (154)	17	2	34 (200)	36,639	0.5	382 (1)
Joda	4,463	1.8	5,826 (131)	10	2	20 (200)	21,703	1.1	4,679 (22)
Kenana	17,750	2.3	31,059 (175)	19	2	38 (200)	91,975	0.7	8,056 (9)
Rabak	29,258	2.5	44,025 (150)	44	2	88 (200)	136,124	0.5	9,011 (7)
Total	78,615	2.4	121,418 (154)	149	2	298 (200)	404,287	0.9	75,021 (19)

* Some students received health education more than one time.

Health education for village residents depended on the baseline prevalence of *S. haematobium* infection. In high prevalence villages (more than 30% at baseline), health education was performed one time per year at the primary schools or village center independently during the project period, however the village residents who lived in less than 30% prevalence attended health education held in the regional primary schools. Thus, the cumulative number of 75,021 village residents received health education for more than 1 hour, which represented 19% of all village residents in the Al Jabalain locality (Table 5). Health education for primary school teachers were held every year for two years, and they were educated intensively for more than 5 hours for them to work as a public health educator to students about schistosomiasis.

The impact of health education was measured by questionnaires received from 244 students at the baseline and 217 students at follow-up assessments (Table 6). The percentages of correct answers to questions about the infection mode (by contact with contaminated water, 95% CI = 1.4323–3.292) and the impacts on health (blocking children learning well, 95% CI  = 1.039–2.545; causing severe diseases if untreated for a long time, 95% CI  = 2.554–6.495) in schistosomiasis significantly increased at the follow-up compared with at the baseline survey. Also the percentage of incorrect answerers to questions about the infection mode of schistosomiasis (by eating unwashed food, 95% CI = 0.331–0.932) was significantly decreased after health education. However, the other questions about clinical manifestations, infection model, preventive methods and the effects of body related to schitosomiasis were not changed significantly after health education intervention (Table 6).

**Table 6:** The effects of health education about schistosomiasis from school students at baseline and follow-up survey by questionnaires.

Variables	Baseline survey		Follow-up survey		OR	95% CI	*P*-value
	Total. No	Answerers'No. (%)	Total No	Answerers' No. (%)			
Did you have hematuria recent 2 months?							
1) Yes	244	35 (14.3)	217	21 (9.7)	1.063	0.622–1.816	0.824
2) No	244	167 (68.4)	217	156 (71.9)	0.968	0.654–1.433	0.872
How do you get schistosome infections?							
1) By eating unwashed food	244	48 (19.7)	217	26 (12.0)	0.556	0.331–0.932	0.026
2) By walking barefoot in a contaminated area	244	59 (24.2)	217	46 (21.2)	0.843	0.544–1.307	0.446
3) By contact with contaminated water	244	154 (63.1)	217	171 (78.8)	2.173	1.432–3.295	0.000
4) By mosquito bite	244	23 (9.4)	217	16 (7.4)	0.765	0.393–1.489	0.430
Why are schistosome infections bad for your health?							
1) Because they cause blood loss	244	82 (33.6)	217	81 (37.3)	1.177	0.803–1.725	0.404
2) Because they cause anemia and malnutrition	244	50 (20.5)	217	48 (22.1)	1.102	0.705–1.722	0.670
3) Because they stop children growing well	244	79 (32.4)	217	60 (27.6)	0.798	0.535–1.191	0.270
4) Because they stop children learning well	244	43 (17.6)	217	56 (25.8)	1.626	1.039–2.545	0.034
5) Can cause severe diseases if untreated for a long time	244	31 (12.8)	217	81 (37.3)	4.073	2.554–6.495	0.000
How do you avoid getting these infections?							
1) Defecating/urinating in latrines	244	91 (37.3)	217	72 (33.2)	0.835	0.569–1.225	0.356
2) Washing hands before eating	244	60 (24.6)	217	56 (25.8)	1.067	0.700–1.625	0.764
3) Avoiding bathing in contaminated freshwater	244	152 (62.3)	217	128 (59.0)	0.870	0.599–1.266	0.468

OR, odd ratio; 95% CI, 95% confidence intervals.

We also checked the basal schistosomiasis-related knowledge of school health teachers before health education (Table 7). They answered higher correct responses about infection mode, health hazards, side effects, and preventive method of schistosomiasis in comparison to those of students. They thought that schools are good sites for deworming programs, and health education is the best method for prevention of schistosomiasis. However, teachers had some worries about the minor side effects after administration of praziquantel.

**Table 7:** The results of questionnaires about schistosomiasis from school teachers before health education.

Category	Total No.	No. of answerers (%)*
1. How do you get schistosome infection?		
1) By eating unwashed food	129	17 (13.2)
2) By walking barefoot in a contaminated soil	129	22 (17.1)
3) By contact with contaminated water	129	92 (71.3)
4) By mosquito bite	129	3 (2.3)
5) No response	129	25 (19.4)
2. Why is schistosome infection bad for your health?		
1) Because they cause blood loss.	129	50 (38.8)
2) Because they cause anemia and malnutrition.	129	51 (39.5)
3) Because they stop children growing well.	129	74 (57.4)
4) Because they stop children learning well.	129	52 (40.3)
5) If untreated for a long time can cause severe diseases.	129	56 (43.4)
6) No response	129	9 (7.0)
3. How do you avoid getting these infections?		
1)Defecating/urinating in latrines	129	73 (56.6)
2)Washing hands before eating	129	40 (31.0)
3) Avoiding bathing in contaminated freshwater	129	117 (90.1)
4) No response	129	3 (2.3)
4. Why are schools good for deworming programs?		
1) Many children are reached easily.	129	78 (60.5)
2) School-children are one of the most infected groups.	129	51 (39.6)
3) Teachers are the best educators and can easily give drugs to the children.	129	81 (62.8)
4) No response	129	2 (1.6)
5. What would you do if a child presented any of vomiting, abdominal pain, headache, body itching and dizziness after mediation of antihelminthics?		
1) Refer the child to the nearest clinic as soon as possible.	129	87 (67.4)
2) These side effects do not need treatment and will disappear spontaneously in a few hours.	129	41 (31.8)
3) No response	129	1 (0.8)
6. How can we prevent the infection of schistosome at schools?		
1) Distributing deworming drugs regularly	129	67 (51.9)
2) Giving health education	129	110 (85.3)
3) Improving the number of latrines in the village	129	58 (45.0)
4) No response	129	3 (2.3)

* Marked one or more of the correct answers.

### The coverage of drug administration of school children and village residents

Praziquantel was administered to 98,586 students and 111,795 village residents in the Al Jabalain locality (Table 8). In case of school students, whole primary school children in the pilot area received adequate amount of praziquantel tablets at the first year of the project, except absentees, ill or refusing children taking medicine. At the second year, mass chemotherapy was done at schools more than 10% prevalence of urogenital schistosomiasis at baseline survey. About 60–70% of school children attended the mass treatment each year, thus cumulative coverage rates of drug administration were higher than 100% at all units, beside Al Jabalain unit (86%, Table 8).

**Table 8:** Cumulative number of participants treated with praziquantel at Al Jabalain locality of White Nile State, Sudan.

Unit	Primary school students			Village residents		
	Total No.	No. of mass treatment	Cumulative No. (%)*	Total No.	No. of visited	Cumulative No. (%)
Al Jabalain	10,226	2	8,837 (86)	58,745	1.7	38,776 (66)
Assalaya	10,444	2	15,167 (145)	59,101	1.4	40,762 (69)
Jazeera Aba	6,474	2	10,439 (161)	36,639	0.5	576 (2)
Joda	4,463	2	6,010 (135)	21,703	1.1	5,952 (27)
Kenana	17,750	2	21,255 (120)	91,975	0.7	12,144 (13)
Rabak	29,258	2	36,878 (126)	136,124	0.5	13,585 (10)
Total	78,615	2	98,586 (125)	404,287	0.9	111,795 (28)

* Some students received drug treatment more than one time.

We also delivered the drug to village residents to control the schistosomiasis. In villages, drug administration was performed at a school base, according to the baseline prevalence of *S. haematobium* infection. Basically, drugs were administered to the village residents who attended the health education. Mass chemotherapy for all village residents was implemented in cases that the prevalence of baseline at each school (village) was more than 30%. Thus, the overall cumulative coverage rate of drug administration of village residents was 28%. However, the coverage rates of each unit varied from 2% to 69%, although the number of visiting-village was different from the baseline prevalence of *S. haematobium* infection.

Questionnaires about the effects of drug treatment were received from 202 students. Fifty-nine percent of them answered improvement in their physical condition after praziquantel therapy. The improvement included feeling more active and healthy (72.7%) and the disappearance of hematuria (18.2%). After praziquantel medication, 10.9% of students complained of adverse effects, such as abdominal pain 50.0%, vomiting 18.2%, and dizziness 13.6% (Table 9).

**Table 9:** The results of questionnaires about praziquantel treatment from school students during the Project period.

Variables	No. of total	No. of answerers (%)
Was your body condition improved after administration of drug?		
Yes	178	105 (59.0)
No	178	46 (25.8)
No idea	178	27 (15.1)
How was your body changed in detail after treatment?		
More active and healthy	77	56 (72.7)
Disappearance of hematuria	77	14 (18.2)
Disappearance of fever	77	3 (3.9)
Disappearance of abdominal pain	77	2 (2.6)
Disappearance of headache No change	77	1 (1.3)
	77	1 (1.3)
Were there any side effects or problems after treatment?		
Abdominal pain	22	11 (50.0)
Vomiting	22	4 (18.2)
Dizziness	22	3 (13.6)
Diarrhea	22	1 (4.5)
Headache	22	1 (4.5)
fever	22	1 (4.5)
Bloody urine	22	1 (4.5)

n = 202.

### The impacts of a drinking water supply facility at the Al Hidaib village

To supply safe drinking water, which is free from cercariae of *Schistosoma* spp., a system of filtered drinking water supply facility was constructed at Al Hidaib village of Al Jabalain unit according to the selection criteria. Al Hidaib was one of the highest prevalence of schistosomiasis among Al Jabalain locality, White Nile State. Also there were no clean water supply facilities in Al Jabalain unit, and it is easy to obtain the water from the Nile River.

We checked the impacts and usage patterns of a drinking water supply facility by questionnaire survey from residents of Al Hidaib village 9 months after construction. As shown in Table 10, 94.3% of answerers used the water supply facility. They obtained water almost every day (86.3% of user), and they usually collected water less than 30 liters per person in a day. Thus, the coverage rate of a drinking water facility at Al Hidaib was about 66% (2000 among 3000 residents) in case of use 30 liter per day per person. The major use of collected water was drinking, making food, or washing clothes or body. The important one was that 81.4% of answerers were felt good health, and 55.9% of them answered saving the time to collect water. However, about 25% of user of water supply facility also collected water from the White Nile River.

**Table 10:** The results of questionnaires for evaluation of common water supply facility at Al Hidaib villages of Al Jabalin unit, White Nile State, Sudan.

Category	No. of total	No. of answerers (%)
1. Have you ever used the common water supply system at Al Hidaib village?		
1) Yes (go to number 2–8)	106	100 (94.3)
2) No	106	4 (3.8)
3) No response	106	2 (1.9)
2. If yes, how frequently do you use the common water supply system at Al Hidaib village?		
1) Every day	102	88 (86.3)
2) Every 2–3 days	102	12 (11.8)
3) Every week	102	1 (1.0)
4) 1–2 times per month	102	1 (1.0)
5) No response	102	0 (0.0)
3. If yes, how much do you collect water from Al Hidaib water supply system at one time?		
1) One bottle (about 30 liter)	102	15 (14.7)
2) Less than one bottle	102	77 (75.5)
3) 2–5 bottles	102	0 (0.0)
4) More than 5 bottles	102	0 (0.0)
5) No response	102	10 (9.8)
4. If yes, what is the major usage of water from Al Hidaib water supply system in your family?*		
1) Drinking	102	100 (98.0)
2) Making meal	102	79 (77.5)
3) Washing the something for eating	102	82 (80.4)
4) Washing the clothes or body	102	81 (79.4)
5) The others	102	47 (46.1)
6) No response	102	0 (0.0)
5. If yes, how do you feel your body condition after using water from Al Hidaib water supply system?		
1) Improved	102	67 (65.7)
2) Same	102	23 (22.5)
3) Decreased	102	11 (10.8)
4) I don't know	102	1 (1.0)
5) No response	102	0 (0.0)
6. If yes, what is the major merit in using the water from Al Hidaib water supply system?*		
1) Save the money	102	20 (19.6)
2) Good for health	102	83 (81.4)
3) Drink clean water	102	12 (11.8)
4) Save the time to collect water	102	57 (55.9)
5) The others	102	19 (18.6)
6) No response	102	11 (10.8)
7. If yes, how much does the water from Al Hidaib water supply system cover in your family?		
1) 100%	102	77 (75.5)
2) more than 70	102	19 (18.6)
3) Around 50%	102	5 (4.9)
4) Around 20%	102	0 (0.0)
5) Less than 10%	102	0 (0.0)
6) No response	102	1 (1.0)
8. If not 100%, where do you collect the water beside Al Hidaib water supply system?		
1) River	25	24 (96.0)
2) Donkey	25	0 (0.0)
3) Commercially bottled water	25	0 (0.0)
4) Private water supply system	25	0 (0.0)
5) No response	25	1 (4.0)

*: Marked one or more of the correct answers (# 4 and 6).

## Discussion

The study was the partial results of comprehensive schistosomiasis control project implemented by KOICA in White Nile State, Sudan. The project reduced the prevalence of *S. haematobium* infection from 28.5% to 13.5% after 6–9 months and increased the awareness of students about the seriousness of schistosomiasis and the preventive measures by health education. This project also showed the importance of clean water supply facility for the control of schistosomiasis. Finally, approximately 200,000 students, teachers, or village residents have been benefited by providing either praziquantel medication, health education, or filtered water supply.

Mass chemotherapy with praziquantel has been employed by many national control programs for schistosomiasis [16]–[20]. Praziquantel treatment reduced the prevalence of *S. haematobium* infection by 87% [18] and reduced infection rates from 54.2% and 51.7% in high- and low- transmission seasons to 30.3% and 1.8%, respectively [19]. In this project, primary school students were the principal target group for treatment and education due to feasibility and the greater benefit of reducing the infection burden in children [21]. The overall *S. haematobium* prevalence of our data (28.5%) was similar to that reported previously for White Nile Province (21.4%) [6]. However, the *S. mansoni* prevalence (0.4%) was significantly lower than that reported previously (10.1%) [6]. It can be explained that detection of fecal *S. mansoni* by egg count may be affected by day-to-day and intra-specimen variation, resulting in the low sensitivity of single stool smear [22]. At 6–9 months post-treatment, the rate of *S. haematobium* infection had been reduced by 52.6% (from 28.5% to 13.5%), which was similar to that during the high-transmission seasons in Mozambique [19]. Surprisingly, the *S. haematobium* prevalence at Khour Ajwal, a village in the project area, increased from 27.3% to 45.6% during the post-intervention monitoring period. It may be due to low coverage of drug administration, poor environmental condition, low attendance rate of children at school and continuous reinfection. Collectively, the survey findings suggest that *S. haematobium* infection is more prevalent than *S. mansoni* infection in Sudan, and repeated exposure to schistosome. Therefore, wide-scale and sustainable chemotherapy is essential to successfully control schistosomiasis.

By analyzing the prevalence after intervention, we found a few interesting characteristics. First, the reduction rate of prevalence was higher in girls than in boys, which meant that boys were more likely to be infected and were reinfected more frequently due to more outdoor activity such as swimming [23]. Second, the highest reduction in prevalence was observed among the moderately infected group (20–25% at baseline) compared with the highly infected group (>40%). Generally, reduction rates in the >60% egg-positive groups were 69.4–87% at 3–24 weeks post-treatment [18], [20]. However, the reduction rates for the highly prevalent groups were around 50% in this study, lower than those previously reported [18], [20] and moderately infected group of this study. These data demonstrated that reinfection occurred more frequently in the highly endemic village than in the moderately endemic one. Third, praziquantel therapy produced little change in the *S. mansoni* prevalence compared with the reduction observed for *S. haematobium*. Similar findings were reported in Niger [20]. Previous reports and our data indicate that a single dose of praziquantel (40 mg/kg) is less effective in curing *S. mansoni* infection than that of *S. haematobium*. Development of resistance to praziquantel is also of concern [24] and should be studied by a well-designed investigation.

In addition to chemotherapy, encouraging self-protective health behaviors may reduce the transmission and reinfection rates of schistosomiasis [25]. Through this project, students received health education 1.8–2.8 times through the project period, thus cumulative coverage rates of health education were 131–175% at a unit level. Kenana unit showed the highest cumulative rate of health education (175%), the reduction rate of *S. haematobium* prevalence was 37.0%, which was below the mean reduction rate 52.6%. On the contrary, Joda unit revealed the lowest cumulative health education rate (13%), but the reduction rate was 69.6%. Thus, there was no correlation between health education coverage rates and reductions in prevalence, which was in agreement with a previous report [26]. In the present study, students showed a general increase in awareness of the health hazard of schistosomiasis and the preventive measures according to the questionnaire survey. However, this knowledge was not connected to prevent the infection in Khour Ajwal village in Al Jabalain unit, so continuous health education is needed at the school level. For this purpose, this project conducted intensive health education course for school health teachers. According to the questionnaire results from health teachers, they emphasized the health education and the role of schools to prevent the reinfection of schistosomiasis. Thus, the intensive program may contribute teachers to enforce the role as a health educator to students. To obtain better outcomes, health education models need to consider social representation and illness experience besides scientific knowledge in order to increase knowledge of schistosomiasis transmission and prevention [27].

The clean and safe water supply is important in reducing the rates of ascariasis, diarrhea, schistosomiasis, and trachoma [28,29]. Villages in the pilot area were settled along the White Nile River; however few facilities provided clean water and sanitation services. Therefore, village residents had no choice but to use water directly from the river. The KOICA project provided a large filtered water supply facility in Al Hidaib village in the Al Jabalain unit. The follow-up survey revealed a reduction in the prevalence at Al Hidaib, from 46.1% to 20.5% of urogenital schistosomiasis, whereas that in the neighboring village in the same unit, Khour Ajwal, increased from 27.3% to 45.6% during the same period. The natural environment and lifestyle of the two neighboring villages were very similar, and mass praziquantel therapy and health education were implemented in both. However, filtered clean water was supplied only to Al Hidaib village residents. This finding may reflect the importance of providing a supply of clean water to reduce reinfection rates as part of efforts to control schistosomiasis. According to the questionnaire survey among Al Hidaib residents, 71.2% of the village residents used only water collected from this water supply facility, and 81.4% of answerers felt better health condition after construction of a drinking water supply facility. Thus, clean drinking water supply facility in Al Hidaib village will contribute to improving the health and life quality of the residents and children by prevention of waterborne diseases including schistosomiasis.

Even though the project achieved the proposed objectives, it was also limited in several ways. First, since the project duration was not long enough to eliminate schistosomiasis, there was a potential for the resurgence of the disease. Second, molluscicides were not used for snail control due to the effects of environment, and it may be one of the factors of high reinfection. Third, this project targeted primarily students; thus, village residents and preschoolers in the villages were not covered fully. Fourth, a filtered water supply facility was provided to only one village, and this was not sufficient to affect the whole target area. Fifth, we used urine sedimentation method. This method is very good to detect the eggs of *S. haematobium* in urine, however the eggs might attach to the wall of tubes or dispensers to lower its sensitivity [30]. Due to these limitations, the results should be interpreted as reflective of the effects of mass praziquantel therapy and health education on the control of urinary schistosomiasis.

In conclusion, mass praziquantel therapy should be the core activity of efforts to control schistosomiasis in the field, which must be integrated with health education and clean water supply. The control activity should last long enough to reduce reinfection and keep low prevalence in the project area.

## Supporting Information

S1 Checklist **STROBE check.**
(PDF)

S1 Appendix **Manual for schistosomiasis control.**
(PDF)

S1 Video **Health education for teachers.**
(AVI)

S2 Video **Health education for school student.**
(AVI)
