# Supplementary material for: Reduction of Urogenital Schistosomiasis with an Integrated Control Project in Sudan
Source: PLoS Negl Trop Dis. 2015 Jan 8;9(1):e3423. doi: 10.1371/journal.pntd.0003423 (PMC4288734; doi:10.1371/journal.pntd.0003423)
Supplement: S1 Appendix — Manual for schistosomiasis control. (PDF) [file pntd.0003423.s002.pdf]

국제빈곤퇴치기여금
Air-ticket Solidarity Contribution | KOREA

# Manual for Schistosomiasis Control

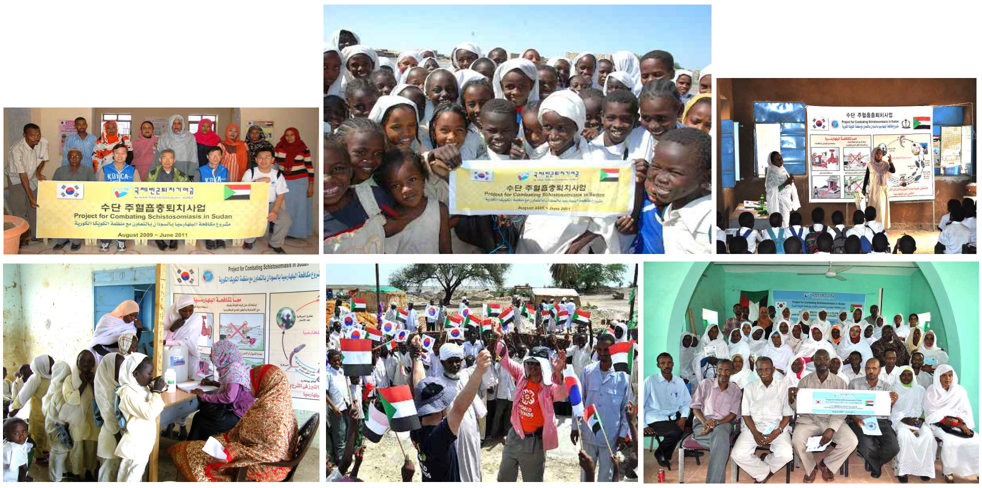


KOICA
한국국제협력단

한국건강관리협회
Korea Association of Health Promotion

Page 1 ~ 82 : “Manual for Schistosomiasis Control” was written by Korean.

# Contents

Acknowledgements ..... 83

Abbreviations ..... 84

Ⅰ. Purpose of the Manual ..... 84

Ⅱ. Overview of the Project ..... 85

1. Timetable for implementation ..... 86
2. Project sites ..... 86
3. Primary plans of the project ..... 88
4. Briefs of implementation activities ..... 88

Ⅲ. The Duties of Korean PM in Sudan and Sudanese Employees ..... 91

Ⅳ. Awareness of Schistosomiasis and Selection of Targets for Field Survey ..... 94

1. Awareness of schistosomiasis ..... 94
2. Selection of targets for field survey ..... 96

Ⅴ. How to Collect and Examine the Samples ..... 99

1. Field survey team ..... 99
2. Flow diagram for the field survey ..... 99
3. Tasks and responsibilities of laboratory technicians ..... 100
4. Collection of urine and stool samples ..... 100
5. Questionnaire and consent for sample collection from school children ..... 102
6. Cellophane thick smear method (Kato's method) ..... 103
7. Urine sedimentation method ..... 106
8. Operation and maintenance of microscope and centrifuge ..... 109
9. Microscopic examination of fecal specimens ..... 115
10. Safety in the parasitology laboratory ..... 119

Ⅵ. Chemotherapy ..... 120

1. Target schools of treatment ..... 120
2. How to calculate praziquantel dose of each person ..... 121
3. Men in charge who administer treatment in schools and villages ..... 122
4. Useful information about treatment ..... 123

Ⅶ. **Health Education for Preventing Schistosomiasis** ······ 126

1. Training for Sudanese health workers ······ 126
2. Health education training for school teachers ······ 127
3. Education for school students and village residents ······ 133

Ⅷ. **Project for Clean Drinking Water Supply** ······ 136

1. Outline of the project ······ 137
2. Implementing plan of the project ······ 141
3. Period of detailed process ······ 145
4. Evaluation and monitoring plan ······ 145
5. Self-management plan after completion of the project ······ 146

Ⅸ. **How to Monitor and Evaluate the Project Implementation** ······ 148

1. Collection of data ······ 148
2. Monitoring and evaluation questionnaire for students and teachers ······ 150
3. Analysis of data ······ 150

Ⅹ. **How to Manage Project Implementation** ······ 153

Ⅺ. **Snail Control by Molluscicide** ······ 154

1. Selection of target area ······ 154
2. Public relations ······ 154
3. Selection of molluscicide ······ 155
4. Methods of employing molluscicide ······ 157
5. Evaluation ······ 158
6. Special instructions/advices ······ 162

ⅩⅢ. **References** ······ 164

# Acknowledgments

This manual is prepared by the Korea Association of Health Promotion (KAHP), which is a Project Management Consultant (PMC) of the Project for Combating Schistosomiasis in Sudan, in collaboration with Korea International Cooperation Agency (KOICA), the Federal Ministry of Health (MOH) of the Republic of Sudan, and others. The names of Organizations which took part in providing this Manual are as below.

Ministry of Foreign Affairs and Trade, Republic of Korea

Korea International Cooperation Agency(KOICA)

Korea Association of Health Promotion(KAHP)

Federal Ministry of Health, Republic of Sudan

National Control Program for Schistosomiasis & STH, Sudan

Ministry of Health in White Nile State, Sudan

KOICA Schistosomiasis Laboratory in Rabak, White Nile State, Sudan

Embassy of the Republic of Korea in Sudan

# Abbreviations

PZQ Praziquantel [the drug used to treat schistosomiasis (bilharzia)]

PM Project Manager

KAHP Korea Association of Health Promotion, which is Project Management Consultant (PMC)

KOICA Korea International Cooperation Agency, which is Korea Governmental Organization to support ODA

MOH Ministry of Health of the Republic of Sudan

# Ⅰ. Purpose of the Manual

The purpose of this Manual is to help community officials and those who are involved in this project implementation manage the Project in White Nile State, Sudan, and to train others to educate and treat schools and communities. The guideline contains brief information about the project, duties of Korean PM and Sudanese employees, methods for survey and treatment, training and health education, management, monitoring and evaluation procedures. At the end there are several forms and materials that used in the Project.

# Ⅱ. Overview of the Project

The Project for Combating Schistosomiasis in Sudan was officially launched in Aljabalain locality, White Nile State, in August 2009. This project was managed by KAHP as a Project Management Consultant (PMC). KOICA provided grant aid within the budget limit of 1,188 million Korean Won (equivalent to 1.2 million US dollars) for this Project, and the Federal MOH of the Republic of Sudan appropriated a counterpart amount from its budget to cover the expenses required for fulfilling the successful implementation of the Project.

The primary objectives of the Project were as follows .

- Reduce the prevalence of schistosomiasis in the project areas significantly by the end of 2011.
- Improve the awareness and practice to prevent the schistosomiasis in the project community
- Provide the drugs and medical equipments for control of schistosomiasis
- Reinforce the environmental infrastructure for the prevention of schistosomiasis in the pilot areas

## 1. Timetable for implementation

The Project implementation began on 21st August 2009 and finished on 30th June 2011.

<Table 1> Work schedule of the Project implementation

| | Time Details | 2009 | | 2010 | | | | 2011 | | Remarks |
|---|---|---|---|---|---|---|---|---|---|---|
| | | 3/4 | 4/4 | 1/4 | 2/4 | 3/4 | 4/4 | 1/4 | 2/4 | |
| Management of laboratory | Selection and dispatch of Korean PM to Sudan | | | | | | | | | |
| | Employment of two Sudanese | | | | | | | | | |
| | Management of the laboratory in White Nile state | | | | | | | | | |
| Provision of materials | Provision of medical equipment, supplies and anthelmintics | | | | | | | | | |
| Monitoring | Dispatch of Korean experts team to Sudan | ① | ② | | ③ | | ④ | ★ | ★ ⑤ | ①~⑤ : scheduled dispatch ★: self-monitoring by KAHP |
| | Sampling survey (400 persons each) | | ① | | | | ② | | | |
| Field survey & treatment | Field survey targeting 10,000 students throughout the year | | ① | ② | ③ | ④ | ⑤ | ⑥ | ⑦ | Quarterly report to KAHP |
| | Treatment with PZQ | | ① | ② | ③ | ④ | ⑤ | ⑥ | ⑦ | Health education after treatment |
| Health education | Development and production of health education materials | | | | | | | | | |
| | Health education for students and residents | | | | | | | | | 50 times |
| | Health education training for teachers | | ① | ② | ③ | ④ | ⑤ | ⑥ | ⑦ | Once per quarter |
| Evaluation | Workshop & Meeting | M | W | | M | | W | | W | |
| | Evaluation & Steering committee | S | | S | | S | E | S | E | |

## 2. Project sites

The Project for Combating Schistosomiasis in Sudan 2009-2011 was scheduled to operate at Aljabalain locality, White Nile State, which was composed of 6 Units. The White Nile State is located in the center of Sudan. Aljabalain locality is located at the east bank of the White Nile River in the southern side of the White Nile State. All villages located adjacent to the White Nile River, where the houses were built with mud-bricks. The local activities of inhabitants were based on agriculture. Most villagers rely on the water of the White Nile River for drinking, domestic usage as well as for the recreational activities.

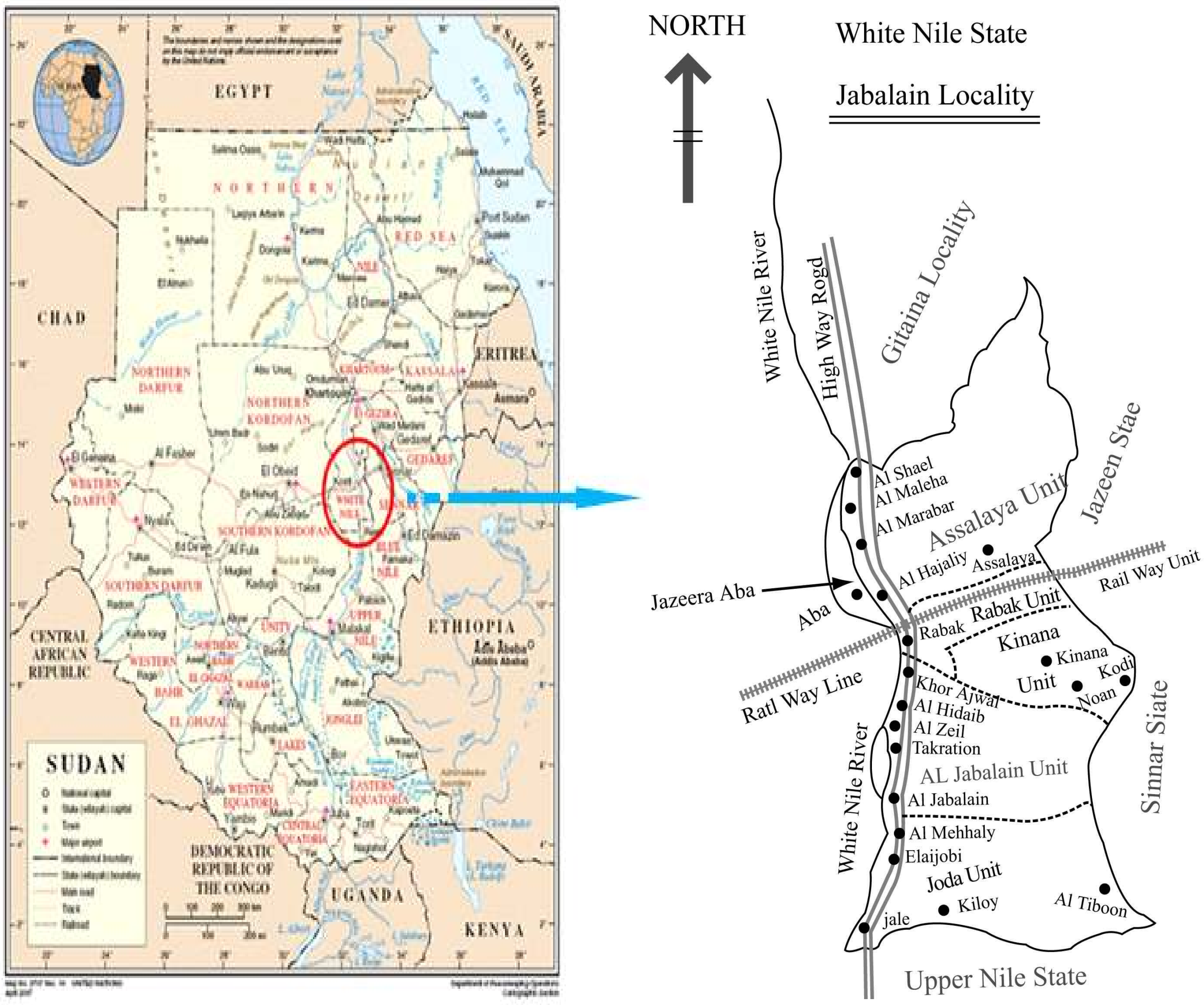


(Figure 1) Location of Aljabalain locality, where is implementation site of the Project Combating Schistosomiasis in Sudan 2009-2011.

<Table 2> Number of students and population of Aljabalain locality of White Nile State, Sudan.

| Number | Unit name | No. of students | Population by age group | | |
|---|---|---|---|---|---|
| | | | Under 15 years old | Over 15 years old | Total |
| 1 | Aljabalain | 10226 | 30347 | 38624 | 68971 |
| 2 | Assalaya | 10444 | 30600 | 38945 | 69545 |
| 3 | Jazeera Aba | 6474 | 18970 | 24143 | 43113 |
| 4 | Kinana | 17750 | 48279 | 61446 | 109725 |
| 5 | Joda | 4463 | 11513 | 14653 | 26166 |
| 6 | Rabak | 29258 | 72768 | 92614 | 165382 |
| | Total | 78615 | 212477 | 270425 | 482902 |

## 3. Primary plans of the project

In Project for Combating Schistosomiasis in Sudan, KOICA took charge of provision of medical equipments, supplies and anthelmintics and KAHP took charge of technical support and local project implementation.

<Table 3> The list of PMC activities of the project

| Items | Details |
|---|---|
| Dispatch of Korean PM and Lab Management | • One Korean PM stay in Sudan<br>• Establishment of laboratory in Sudan<br>• Provide payment for two Sudanese employees |
| Management of Drugs and Equipments | • PZQ for treatment of schistosomiasis (450,000 persons)<br>• Two vehicles, and equipments for parasitological examinations<br>• Transportation fee |
| Field Survey and Chemotherapy | • For management of schistosomiasis, field survey for diagnosis and evaluation of schistosomiasis<br>• Implementing chemotherapy in cases of positive cases<br>• Cost for transport and other miscellaneous items |
| Education about Schistosomiasis for School Children and Residents | • Development of materials for education about schistosomiasis<br>• Education to school children and residents in the project areas<br>• Workshop for teachers<br>• Lecture fee for speakers<br>• Printing informative leaflets, posters, billboard etc. |
| Supply of drinking water | • To supply safe water permanently through ground water development in pilot areas |
| Dispatch Korean Experts | • Dispatch of Korean experts |
| Management and evaluation | • Meetings for evaluation and management between Korean and Sudanese experts |

## 4. Briefs of implementation activities

| Tasks | Objectives | Contents | Responsibility |
|---|---|---|---|
| **1. Dispatch of Korean experts to Sudan** | - To manage whole project and to transfer Korean advanced technologies on parasite control through total of 5 times of Korean experts/staffs' dispatch to Sudan | ○ **Dispatch schedule of Korean experts**<br>: Total 5 times for 7-14 days each at September and December 2009, June and December 2010, June 2011<br>○ **Duty of Korean PM in Sudan and Sudanese employees**<br>- Management of the Project under the coordination of MOH, Sudan | Korean PM, Korean experts/ Staffs |

| Tasks | Objectives | Contents | Responsibility |
|---|---|---|---|
| | - To increase the efficiency and effectiveness of the project through one-year dispatch of Korean PM to Sudan and employment of Sudanese staffs at the laboratory in the project site. | - Management of laboratory under the participation of two Sudanese employees for the project<br>- Field survey and drug delivery for schistosomiasis in the project areas<br>- Development of guidelines for schistosomiasis control in Sudan<br>- Provision of education about schistosomiasis<br>- Development and supply of health education materials<br>- Development of drinking water in the pilot areas | |
| **2. Field survey and Chemotherapy** | - To select treatment group based on the results of field survey on the prevalence of schistosomiasis among 1st, 3rd and 5th grade children of primary school in the project areas<br>- To treat residents with PZQ in Aljabalain and to control on a regular basis through selection of intensive control areas | ○ **Field survey**<br>- Target : total of 10,000 students<br>- Period : December 2009 ~ December 2010<br>- Method of examination : Cellophane thick smear (or Kato method) method for *Schistosoma mansoni* (stool sample) and urine sedimentation method (or urine filter holder method) for *Schistosoma haematobium* | Korean and Sudanese PM, Sudanese health workers |
| **3. Health education for students and residents** | - To provide sufficient knowledge on schistosomiasis to students and residents<br>- To promote self-care health awareness of students and residents for prevention of re-infection | ○ **Target** : Students, 50,000; Residents, 50,000<br>○ **Place** : playground/classroom in the school of project areas<br>○ **Instructor** : two or three experts in parasitology or public health<br>○ **Time** : total of 50 hours (1 hour/time) | Korean and Sudanese PM, Sudanese experts |
| **4. Health education training for teachers** | - To train teachers to be health education experts who can deliver sufficient knowledge of schistosomiasis to students | ○ **Training organization**<br>: National Control Programe of Schistosomiasis & STH<br>○ **Target** : 200 teachers (1~2 per school)<br>○ **Instructor** : two or three experts in parasitology or public health<br>○ **Time** : total of 35 hours (5 hour / time) | Korean and Sudanese PM, Sudanese experts |
| **5. Development of health education materials** | - To prevent re-infection after treatment through health education by using various materials | ○ **Developer** : National Control Program for elimination of schistosomiasis in Sudan/Korean PM<br>○ **Contents** : schistosomiasis transmission, clinical sign & symptom, prevention method, etc. | Korean and Sudanese PM, Sudanese experts |

| Tasks | Objectives | Contents | Responsibility |
|---|---|---|---|
| 6. Establishment of billboard | - To prevent schistosome infection through a large outdoor structure in the main road or primary school. | ○ **Developer** : National Control Programe of Schistosomiasis & STH/Korean PM<br>○ **Contents** : Schistosomiasis transmission, clinical sign & symptom, prevention method, etc.<br>○ **Place** : Primary/secondary schools in project areas<br>○ **Size and Quantity** : 75cm×110, 10 ea | Korean and Sudanese PM, Sudanese experts |
| 7. Development of guideline for schistosomiasis control | - To establish institutional capacity to control schistosomiasis by applying developed guideline to other areas in Sudan | ○ **Contents**<br>- Sampling method for field survey and result analysis<br>- Management of laboratory and provided materials<br>- Examination method, treatment, health education, questionnaire survey, etc.<br>○ **Participants**<br>- Korean PM, Sudanese senior researcher & technician<br>- National Control Programe of Schistosomiasis & STH<br>- Two Korean experts to manage the project in Korea<br>○ **Utilization plan** : Provision of guideline to heavy endemic areas | Korean and Sudanese PM, Korean and Sudanese experts |
| 8. Supply of drinking water | - To supply safe drinking water, which is free from cercariae of *Schistosoma* spp. | ○ Selection of the area where to construct a system of filtered drinking water supply by Korean PM<br>○ Establishment of billboard in front of water tank<br>○ Quantity : 1~5 in the project areas | Korean and Sudanese PM, Korean and Sudanese experts |
| 9. Monitoring and consultation | - To analyze the situation in the community and its project and determine whether the inputs in the project are well utilized<br>- To identify problems facing the community or project and finding solutions<br>- To use lessons from one project experience on to another | ○ Method : On-site monitoring by quarterly dispatch of Korean experts to Sudan | Korean and Sudanese PM, Korean and Sudanese experts |
| 10. Workshop | - To strengthen effectiveness of the project through joint workshop with implementing agencies, Sudan government officials, etc.<br>- To transfer Korean advanced technologies to Sudanese staffs | ○ **Time**<br>- 1st workshop in the 2nd dispatch of Korean expert to Sudan (Dec 2009)<br>- 2nd workshop in the 4th dispatch of Korean experts to Sudan (Dec 2010)<br>- 3rd workshop in the 5th dispatch of Korean experts to Sudan (Jun 2011) | Korean and Sudanese PM, Korean and Sudanese experts |

# Ⅲ. The Duties of Korean PM in Sudan and Sudanese Employees

Woo-Hyun Kong, Section Chief of KAHP had resided in the KOICA Schistosomiasis Laboratory at Rabak, White Nile State, Sudan from October 25, 2009 to October 24, 2010, to implement project activities like management of equipments and supplies, establishment of public health infrastructure, technical support on parasite exam and control, etc.

**<Table 4> List of activities for project management by Korean PM in Sudan**

| Division | Contents |
|---|---|
| Field survey and treatment | • **Field survey** : total of 10,000 students among 1st, 3rd and 5th grade children of primary schools in Aljabalain, White Nile State of Sudan<br>• **Coordinated plan with treatment and health education** : health education for students and residents should be conducted after treatment (more than 1 time every quarter mainly in high prevalence area)<br>• Use WHO recommended strategy for PZQ administration. |
| Maintenance of laboratory | • Place : Rabak in White Nile State, provided by Sudanese MOH<br>• Management of laboratory under the participation of two Sudanese employees (senior researcher, technician)<br>• Management of medical equipments and drugs<br>• Register management for equipments and expendables by Sudnaese employees<br>• Management of anthelminthics (Distribution and treatment plan) by Korean and Sudanese PM |
| Development of health education materials & health education training for teachers | • Development of health education materials<br>: poster, plastic sheet, billboard, booklet, PPT file, etc.<br>• Health education training for 200 school teachers<br>- Development of PPT file to provide school teachers with health education training<br>- Issuance of certificate of completion for trained teachers |
| Health education for students and residents | • To provide 50,000 students and 50,000 residents with health education for prevention of schistosomiasis |
| Development of guideline (manual) | • Selection of survey population and result analysis<br>• Management of laboratory and provided materials<br>• Examination method, treatment, health education, questionnaire survey, etc. |
| Supply of drinking water | • Selection of the area for supply of filtered drinking water<br>• Establishment of billboard in front of water tank |
| Support of project evaluation materials | • **Indicator**<br>- Number of participating schools, number of trained teachers, number of health education activity and awareness questionnaire for health education<br>- Distribution of anthelmintics, treatment coverage<br>- Contents of health education activity, condition of anthelmintics<br>• **Evaluation method**<br>- Prevalence change of schistosomiasis<br>- Technique transfer and capacity building : Schistosomiasis control manual<br>- Health awareness of students and residents : result analysis of questionnaire |

| Division | Contents |
|---|---|
| Support of Korean experts' activity in Sudan (short-term dispatch) | • **Project monitoring by Korean experts**<br>- Identification of health awareness through sampling survey and questionnaire<br>- Number of participating schools, number of trained teachers, number and type of health education activity<br>- Distribution of anthelminthics, treatment coverage<br>- Contents of health education activity, condition of anthelminthics<br>• **Support of guideline(manual) development**<br>- Support of guideline development of Sudanese MOH and National Control Program of Schistosomiasis & STH |
| Others | • Development of global partnership with project implementing organization |

### <Table 5> Work schedule of Korean PM in Sudan

| Division | Year Details | 2009 | | 2010 | | | Remarks |
|---|---|---|---|---|---|---|---|
| | | 3/4 | 4/4 | 1/4 | 2/4 | 3/4 | |
| Dispatch of PM to Sudan | Residence of PM in Sudan | | | | | | |
| Management of laboratory | Participation of two Sudanese employees | | | | | | |
| | Management of laboratory in White Nile State | | | | | | |
| Provision of laboratory equipment | Equipments, supplies and drugs | | | | | | |
| Monitoring | Dispatch of Korean experts to Sudan | ① | ② | | ③ | | |
| | Sampling survey (400 persons) | | ① | | | | |
| Field survey and treatment | Field survey targeting 10,000 students throughout the year | | ① | ② | ③ | ④ | |
| | Treatment with PZQ | | ① | ② | ③ | ④ | health education after treatment |
| Health education | Development and production of health education materials | | | | | | |
| | Health education to students and residents | | | | | | 50 times |
| | Health education training to school teachers | | ① | ② | ③ | ④ | quarterly implementation |
| Evaluation | Workshop (in Khartoum or White Nile State) | | | | | | |
| | Meeting (in White Nile State) | | ① | ② | ③ | ④ | |

<Table 6> List of Project reports

| Division | Deadline | Details | Remarks |
|---|---|---|---|
| Progress report | Quarterly | ◦ Activity plan and achievement | |
| Laboratory management | Nov. 2009 | ◦ Plan of laboratory management<br>◦ Lab. log, etc. | |
| Health education | 〃 | ◦ Development plan of health education materials<br>◦ Establishment of health education plan | |
| Field survey | 〃 | ◦ Field survey and treatment plan<br>◦ Guideline of treatment strategy | |
| Equipments and supplies | 〃 | ◦ Utilization plan of provided equipments and supplies<br>◦ Management of equipments and supplies | |
| Anthelminthics | Dec. 2009 | ◦ Establishment of post-treatment plan<br>◦ Management of anthelmintics | |
| Dispatch of PM to Sudan | Nov. 2010 | ◦ Result of PM's activity in Sudan<br>◦ Development of guideline for schistosomiasis control | |

# Ⅳ. Awareness of Schistosomiasis and Selection of Targets for Field Survey

## 1. Awareness of Schistosomiasis

### A. What is schistosomiasis (also known as bilharzia, bilharziosis or snail fever)?

Schistosomiasis is a parasitic disease caused by several species of the genus *Schistosoma* worms. These worms live in the veins around the intestines or bladder. The female worms lay eggs that are released in the stool or urine and hatch in water.

The larvae, miracidia, hatch from these eggs and then infect a fresh-water snails. They develop and multiply in the snail and after about a month, new larvae, cercariae, emerge from the snail into the water. The cercariae actively seek out a person in the water. When these larvae meet a person, they penetrate the person's skin to begin a new infection. Schistosomiasis is particularly common among people who are often exposed to contaminated water, such as children, fishermen and farmers in irrigation channels. The worms cause blood loss, anemia, enlargement of the liver and spleen and growth retardation. Children who are infected may be too tired to go to school, or too tired to work.

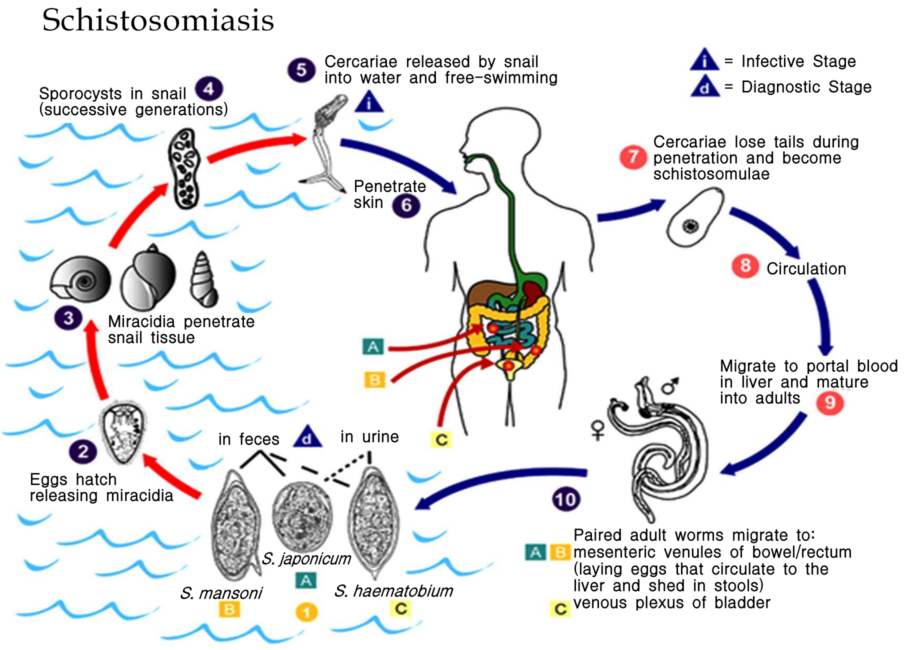


(Figure 2) Life cycle of *Schistosoma* spp. (Source : CDC)

### B. How does schistosomiasis take place?

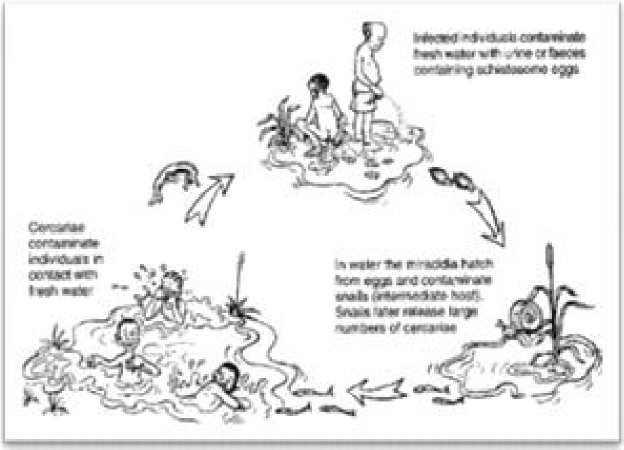


People get infected while in contact with contaminated water infested with schistosomes. The water is contaminated if people have urinated or defecated in or near it. Contaminated water contains cercaria (larval form of schistosome) worms, which are too small to be seen. These worms can penetrate the human skin. Infection takes place when people stand, bathe, play or swim in contaminated water. Infection through drinking contaminated water is possible but much less than through the skin.

### C. How does schistosomiais diagnose?

Microscopic identification of eggs in stool or urine is the most practical method for diagnosis. Eggs can be present in the stool in infections with *S. mansoni* or *S. japonicum*, and their detection will be enhanced by repeated examinations.

Eggs can be found in the urine in infections with *S. haematobium*. Detection of *S. haematobium* eggs will be enhanced by centrifugation and examination of the sediment. Investigation of *S. haematobium* should also include pelvic x-ray or bladder ultrasonography, which is highly characteristic of chronic infection.

Tissue biopsy may demonstrate eggs when stool or urine examinations are negative. The eggs of *S. haematobium* are ellipsoidal with a terminal spine, *S. mansoni* eggs are also ellipsoidal but with a lateral spine, *S. japonicum* eggs are spheroidal with a small knob. Antibody detection can be useful in both clinical management and for epidemiologic surveys.

### D. How can you prevent schistosomiasis?

People should use latrines instead of urinating or defecating in or near the water. Avoid swimming or wading in freshwater when you are in countries in which schistosomiasis occurs. Swimming in the ocean and in chlorinated swimming pools is safe. If water is left to stand for 48 hours before use at home the cercariae of *Schistosoma* spp. will die. People who live in schistosomiasis endemic areas should take PZQ at least once a year.

**E. What are the signs and symptoms of schistosomiasis?**

Most people with schistosomiasis remain without symptoms. However, the severity of disease is related to the number of worms being harbored by the infected person. Each female worm produces 300-3000 eggs per day and lives for about 3.5 years. Only 50% of the eggs come out of the body with faeces or urine. The rest are trapped in the victim's body and it is these eggs that cause the symptoms of schistosomiasis. The symptoms or signs of schistosomiasis include diarrhea, bloody stool or urine, anemia, abdominal pain, nausea, fatigue, drowsiness, liver damage and organ enlargement. School age children generally have many worms inside them. This is called a heavy infection. Children with very heavy infection can suffer impaired mental development and can also experience extensive liver damage, bleeding and death. The damage caused by schistosomiasis occurs gradually, and the longer the person has been infected, the worse the damage may be. Treatment of children at a young age can provide a long-lasting reduction in the number of eggs trapped in the body and therefore prevent severe liver damage through their lives.

F. How does treat schistosomiasis?

PZQ is the drug of choice for treatment of all schistosome infections. For infection with *S. mansoni* and *S. haematobium*, a single dose of 40 mg/kg body weight produces cure rates of 63% to 90%, while three doses of 20 mg/kg body weight in a single day give rates that approach >95%. Even if the drugs do not cure the infection, they will reduce the total number of eggs produced. This greatly reduces the risk of developing serious symptoms by complications. Administration of the drug while the worms are in the schistosomula or immature stages seems to have little effect and may actually worsen acute-stage symptoms. Both periportal fibrosis and bladder wall thickening and polyps respond to PZQ therapy as evaluated by ultrasonography. Annual retreatment may be necessary in advanced cases. PZQ has been used in mass distribution programs and is shown to be safe to use during pregnancy.

## 2. Selection of targets for field survey

The survey provides information concerning the burden of schistosomiasis in a community, and enables judgement to be made as to the need for intervention. Data collected from

children attending schools are generally representative of the situation in the community. When a survey is organized to assess the need for control measures, 200-250 individuals should be an adequate sample for each ecologically homogeneous area in order to evaluate prevalence and intensity.

**The target population for the survey could be primary school children because:**

- schools are accessible easily
- the peak of prevalence of schistosomiasis is to be found in primary school age group
- this age group suffers from nutritional deficiencies because children are undergoing a period of intense physical and mental development
- experience shows there is generally good compliance from children and parents.

The Project was programmed to operate at Aljabalain locality, White Nile State, which was composed of 6 Units. The number of total population of Aljabain locality was 482,902. Among them, 78,615 persons were primary school children. The target numbers for parasitological examination are approximately 10,000 of them.

We selected the target population (approximately 10,000) from total primary school children (78,615) in Aljabain locality as follows.

1) Mean number of each school was calculated by dividing student number (column B in Table 7) by school number (column A in Table 7). Each school was composed of 6$^{th}$ grade. So Mean numbers of 1$^{st}$, 3$^{rd}$ and 5$^{th}$ graders of each school (column C in Table 7) were half of mean number of each school.

2) The numbers of total students (column B, 78,615 persons) were approximately 7.8 times (conversion factor) more than target number (approximately 10,000). Converted numbers of students of each Unit (column D in Table 7) were calculated by dividing of total student numbers of each Unit (column B in Table 7) by 7.8.

3) Numbers of surveyed schools (column E in Table 7) were calculated by dividing converted number (column D in Table 7) by mean numbers of 1$^{st}$, 3$^{rd}$ and 5$^{th}$ graders of each school (column C in Table 7). This number meant the average number of schools at each Unit to examine approximately 10,000 students at Aljabalain locality.

Since there were some differences of students' number of each school, we substantially determined the number of schools to collect samples at each Unit by a number of averages ± 20%.

4) The target schools where we collected the samples were finally selected according to the number of surveyed schools considering the location, total number of students, accessibility to school, sex of students, environmental condition of school, etc. During determining the target schools, Korean and Sudanese PM closely discussed which were the representative schools to check the prevalence of schistosomiasis. In one school, we collected the samples around 80-250, but not more than 300. If the sample size of one school is less than 100, all students should be collected.

<Table 7> The serial steps to select the target number (approximately 10,000) from primary school children of Aljabalain locality (78,615), White Nile State

| | | Column A | Column B | Column C | Column D | Column E |
|---|---|---|---|---|---|---|
| No. | Unit name | No. of primary schools | No. of students | Mean No. of 1st, 3rd and 5th graders of each schools | Converted number | No. of surveyed schools (range, ±20%) |
| **1** | Aljabalain | 30 | 10,226 | 170 | 1,311 | **8 (6-10)** |
| **2** | Assalaya | 29 | 10,444 | 180 | 1,339 | **8 (6-10)** |
| **3** | Jazeera Aba | 18 | 6,474 | 180 | 830 | **5 (4 - 6)** |
| **4** | Kinana | 34 | 17,750 | 261 | 2,276 | **9 (8-10)** |
| **5** | Joda | 10 | 4,463 | 223 | 573 | **3 (2 - 4)** |
| **6** | Rabak | 48 | 29,258 | 305 | 3,751 | **13 (10-16)** |
| | **Total** | **169** | **78,615** | **233** | **10,080** | **46 (36-56)** |

# V. How to Collect and Examine the Samples

## 1. Field survey team

A team composed of 1 Korean PM in Sudan (team leader), 1-2 Sudanese PM, 4 Sudanese laboratory technicians, 4 Sudanese health workers and 1 Sudanese driver undertook field survey and drug distribution.

<Table 8> Organization of team for field survey and drug distribution

| Position | Duty | No. of person |
|---|---|---|
| Korean PM | • general management of the Project in Sudan | 1 |
| Sudanese PM | • assistance of Korean PM, Health education | 1-2 |
| Laboratory technicians | • preparing and reading the slides<br>• recording the results<br>• cleaning or safe disposal of contaminated material<br>• labeling the stool/urine containers<br>• collecting the samples | 4 |
| Health workers | • labeling the stool/urine containers<br>• collecting the samples<br>• questionnaire collection<br>• reception<br>• assistance of health education and drug delivery | 4-6 |
| Driver | • drive | 1 |

## 2. Flow diagram for the field survey

| Time | Activity |
| --- | --- |
| The day before start | Preparation of materials for sample collection, health education and questionnaire |
| 1st day early morning | Health education and registration ⇩ Delivery of questionnaire and sample containers |
| 1st day after meal | Receive the questionnaire and consent ⇩ Collection of urine and stool samples |
| 1st day afternoon | Examination of samples at Center in Rabak ⇩ Keep the unexamined samples at 4℃ refrigerator |
| 2nd day | Record the results and report them to PM |

## 3. Tasks and responsibilities of laboratory technicians

- preparing and reading the slides fo feaces and urine
- recording the results
- cleaning or safe disposing of contaminated materials
- labeling the stool/urine containers
- collecting the samples

## 4. Collection of urine and stool samples

### A. Preparation of materials for sample collection (one day before leaving)

Materials for collection of specimens should be packed at the main laboratory on the day before leaving field survey. The lists of materials for collection of the urine and stool samples are as follows :

1) Stool container : Clean, wide-mouth plastic container with a tight-fitting lid. Approximate 50 ml. The cap is provided with a sample taking spoon that proves helpful in lifting adequate scoop of samples.

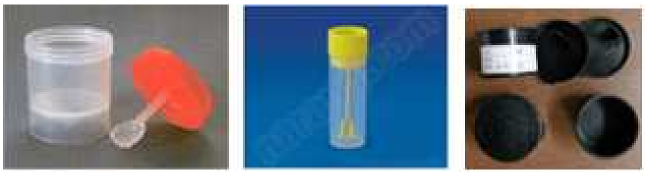

(Figure 3) Various types of stool containers.

2) Urine container : Clean, plastic containers, has cap that work in a press and fit manner and make these containers leakproof. Approximately 15-50 ml.

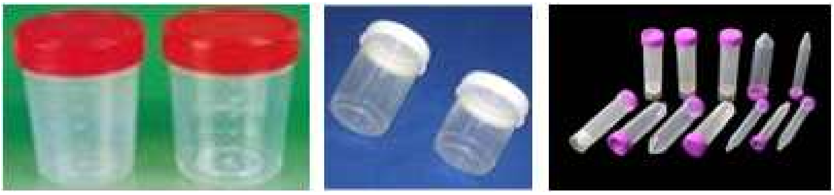

(Figure 4) Various types of urine containers

3) Tube stands : They need to prevent the spilling the urine samples when conical tubes are used for collection.
4) Big container for transportation : approximately 20-30 liters
5) Disposable gloves : Medical examination grade, latex gloves, textured, powder-free, non-sterilized
6) Oil markers and pencils
7) Label paper : It needs to mark the urine containers in cases of reuse.
8) Notebooks for recording
9) Questionnaire (if necessary)

**B. Collection of samples, and sometimes health education and/or questionnaire survey**

1) The collection team was composed of some or all of the followings : one Korean PM in Sudan, one Sudanese PM for health education, six health workers (officials of White Nile State MOH), and one driver.
2) They received written informed consent at the questionnaire sheet or oral informed consent from each school child.

3) Stool and urine samples were collected to examine the parasite infection, especially *Schistosoma mansoni* and *Schistosoma haematobium* infection, from $1^{st}$, $3^{rd}$ and $5^{th}$ graders of primary schools at the sentinel schools of project area.
4) Containers for fecal and urine specimens were delivered in the morning, and then stool and urine samples were collected at same day (usually within 6 hours, or around 2-3pm before leaving school), where the teachers influentially facilitated the collection process.
5) Beside collection of samples, they sometimes educated the students about schistosomiasis and health promotion, and received the questionnaire to obtain the information about health condition, schistosomiasis and water-contact patterns of school children.

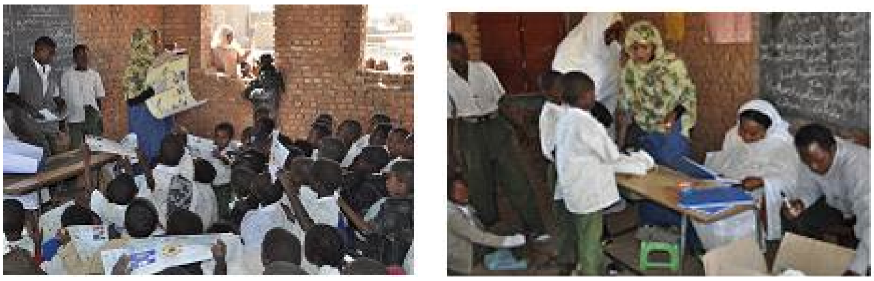

(Figure 5) Education of students about schistosomiasis and health promotion as well as answering the questionnaire survey before collection of samples.

C. Precaution and transportation

1) All specimens should be handled carefully, since each specimen represented a potential source of infectious organisms, including bacteria, viruses, fungi, and parasites. Every specimen was identified with the following information; student's identification number, school name, and the date the specimen was collected.
2) The collected samples were immediately transferred to the main laboratory and were processed according to cellophane thick smear (or Kato-Katz) method for *Schistosoma mansoni* (stool sample) and urine sedimentation method (or urine filter holder method) for *Schistosoma haematobium.*

## 5. Questionnaire and consent for sample collection from school children

To check the personal and socio-behavioral factors in schistosome species infection, we received questionnaire from primary school students while we collected stool and/or urine

samples. The questionnaires were composed of two parts, namely one was for information about health condition and schistosomiasis, and the other part was for water-contact patterns.

Teachers or Sudanese health workers thoroughly explained to the students until they well understood. After oral informed consent was obtained, they were interviewed with all school children about questions individually, and their verbal responses were written.

## 6. Cellophane thick smear method (Kato's method)

### A. Materials

1) Microscope
2) Soaked cellophane paper in glycerine-malachite green solution : see below
3) Microscope slide glasses : 75×25×1mm, twin frosted end.
4) Wooden applicator (or stirring rod) : 15cm×diameter 2mm
5) Tweezers (Pincettes) : 10-20cm length
6) Rubber stopper : 2-3cm diameter
7) Pencils

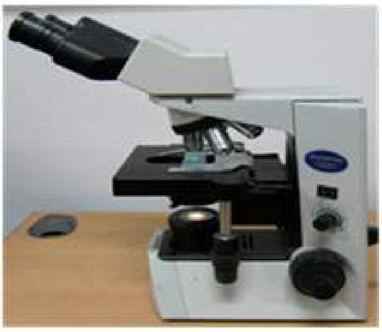

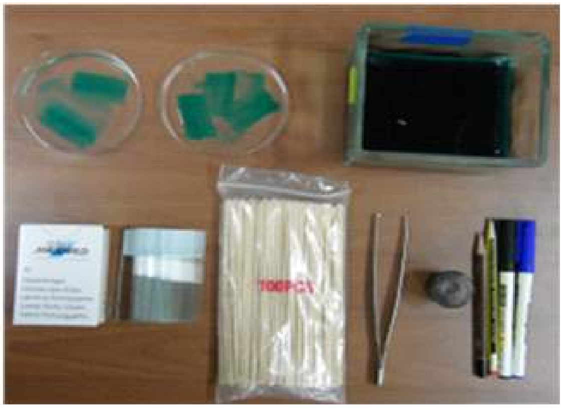

### B. Procedures

1) With a wood applicator, around 50-100 mg of the stool sample are transferred to ordinary microscopic slide glass (Figure 6, ①)
2) Covered with a soaked cellophane (Figure 6, ②)
3) Pressed with rubber stopper to distribute the smear evenly (Figure 6, ③)
4) The slides were dried in room temperature for 20-30minutes and then examined with microscope (Figure 6, ④)
5) The entire 25×35mm cellophane smear was systematically examined with the low-power objective (10×) of microscope, and any suspicious objects was examined

with the high-power objective (40×). Count the whole numbers of eggs at each slide glass. Microscopy was done with 15 w illuminator under 10-40× magnification for 2 to 3 minutes. One smear was examined for each fecal specimen (Fig. 6, ⑤)

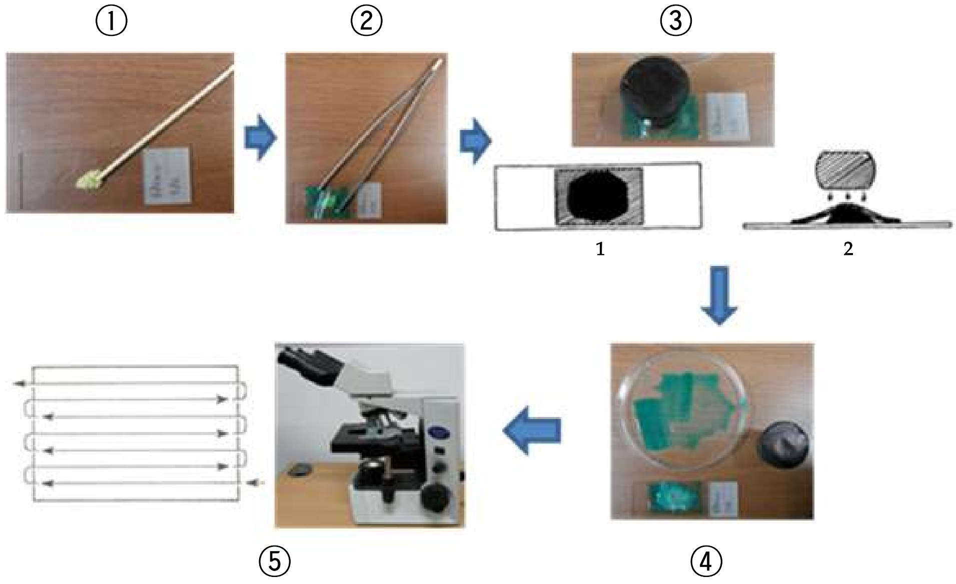


(Figure 6) Serial steps for preparation of cellophane thick smear method

*S. mansoni* eggs were yellowish brown and measured 114-180 µm long by 45-73µm wide. The eggs were elongate and ovoid, and had a large lateral spine projecting near one end. The Kato's method was a simple and sensitive quantitation technique in the field. Occasionally, *S. mansoni* eggs were detected in the urine.

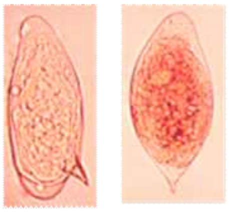

Egg of *Schistosoma mansoni*(left), and *Schistosoma haematobium*(right)

**Key points for laboratory diagnosis in *Schistosoma mansoni* infection**

1. Eggs cannot be detected in stool until the worms mature (may take 4 to 7 weeks after initial infection).
2. In very light or chronic infections, detection of eggs in stool is very difficult; therefore, repeated examination of multiple stool samples is recommended. Biopsy and/or immunologic tests for antigen or antibody may be helpful in diagnosis of these patients.
3. The zinc sulfate floatation concentration method should not be used because the eggs do not float. The sedimentation method is recommended.
4. It is necessary to tap the coverslip to move eggs lateral; the lateral spine may not be visible if the egg is turned on its side.
5. Occasionally, eggs of *S. mansoni* are detected in the urine (crossover phenomenon).
6. Patients who have been treated should be followed-up by ova examinations for up to 1 year to evaluate treatment.
7. In active infections, the eggs should contain live or mature miracidia. Examination for the confirmation of flame cell activity must be performed with fresh specimens, using the microscopic wet mount test or the hatching test; no preservatives can be used prior to these two tests.
8. During the hatching test, the light must not come too close to the surface of the water, since excess heat can kill the liberated larvae. Also, the water should be examined about every 30 min for up to 4 h.

**Soaking (glycerine-malachite green) solution for cellophane paper**

1. Materials

① Cellophane paper : medium thickness (No. 600, 40～50μm), hydrophilic, transparent, not moisture-resistant, measuring off 25×35mm.

② 6ml of 3% malachite green solution

- Sigma Catalog number M9015, biological stain substance, toxic (poisonous) and infectious, storage at room temperature.

③ 500ml of pure glycerin

- Glycerol, Sigma Catalog number G9012, storage at room temperature.

④ 500ml of distilled water (or 6% phenol solution)

⑤ Phenol

- Sigma Catalog number P4557, toxic (poisonous) and infectious substance, storage at 0~5'C

2. Methods

① Prepare the 3% malachite green solution by adding 0.3g malachite green powder to 10ml distilled water.

② Take 6ml of 3% malachite green solution, and add 500 ml distilled water or 6% phenol solution (prepared 6% phenol solution by adding 30ml of phenol to 470ml of distilled water).

③ Add 500ml of pure glycerin slowly with stirring.

④ Store in a glass or plastic bottle at room temperature.

⑤ Cellophane paper covers measuring 25×35 mm is soaked in glycerin-malachite green solution at least for 24 hours.

## 7. Urine sedimentation method

### A. Materials and devices required

1) Microscope :
2) Centrifuge : Max RPM 3,600rpm, Max RCF 1,750g, Max capacity 15ml×6places, Angle type

3) Vortex mixer
4) Tube stand : Have 10-20 holes for 15-50ml centrifuge tubes
5) Disposable transfer pipette : 1-5ml
6) Microscope slide glasses : 75×25×1mm, twin frosted end
7) Microscope cover glasses : 22×22mm
8) Urine collection conical tube : Polystyrene, 15ml, conical shape
9) Oil markers and pencils

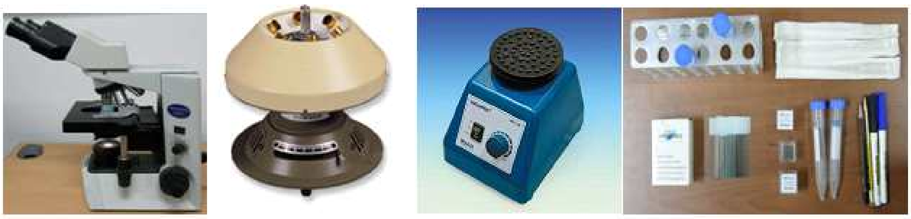

## B. Procedures

1) First, observe whether the urine samples are gross or occult hematuria, or not (Figure 7, ①).
2) Urine is collected at a clean 15ml centrifuge tube, just adjust 10ml to examine *S. haematobium* eggs. If urines are not collected at 15ml conical tube, they should be transferred into clean 15ml centrifuge tubes. If collected urines are not enough for 10ml, the real volume of urine is marked in the tube (Figure 7, ②).
3) Individual tubes, strip tubes and/or adapters must always be loaded symmetrically to ensure proper balance (Figure 7, ③).
4) The tube containing specimen is placed in a centrifuge funnel fitted with 15ml conical tube, and centrifuged 1,500rpm for 5min (Figure 7, ④).
5) After centrifugation, the supernatant fluid is discarded, and the sediment is mixed with vortex for 10second, and then examined all of the pellet under the microscope (Figure 7, ⑤).
6) To examine the urine sediment, 3 drops of mixing urine sediment with 1 ml disposable transfer pipette is placed in on a microscopic slide glass and then placed cover glass on top of sediment (Figure 7, ⑥).
7) The entire 22×22 mm cover glass is systematically examined with the low-power objective (10×), and any suspicious objects may then be examined with the high-power objective (40v). Count the whole numbers of eggs at each slide glass. Microscopy

is done with 15 w illuminator under 10-40× magnification for 2 to 3minutes. One smear is examined for each urine specimen (Figure 7, ⑦).

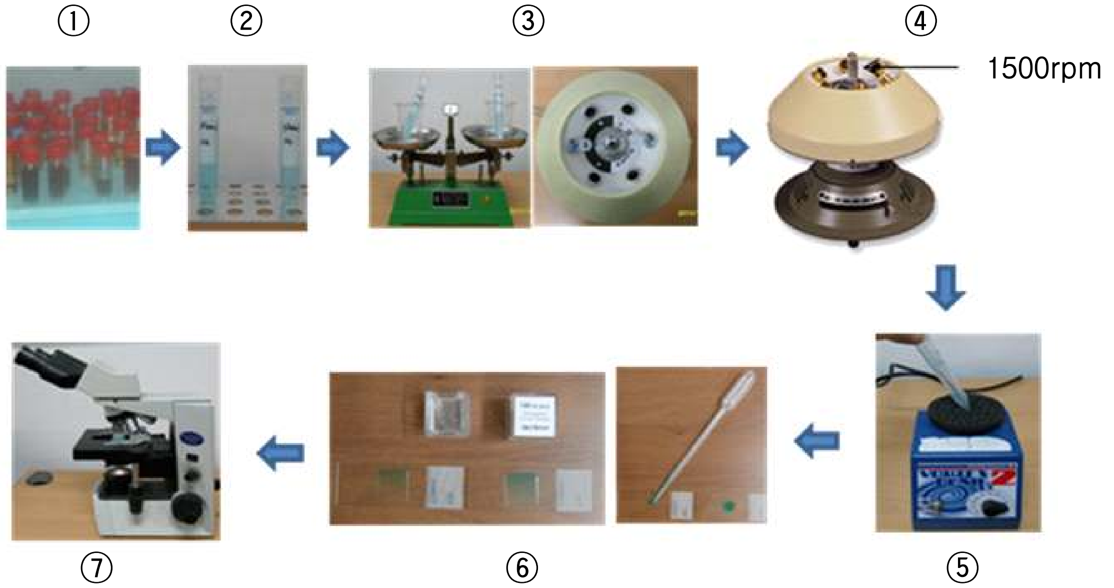


(Figure 7) Serial steps for preparation of cellophane thick smear method

*S. haematobium* eggs are usually detected in the urine, although in heavy infections they may also be found in the stools. Fully embryonated eggs without an operculum (112 to 170μm by 40 70μm) escape from the body in the urine. The eggs are light yellowish brown and contain a conspicuous terminal spine.

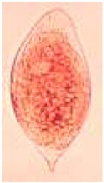

**Key points for laboratory diagnosis in *Schistosoma haematobium* infection**

1. Eggs cannot be detected in urine until the worms mature (may take up to 3 months after initial infection).
2. In very light or chronic infections, egg detection may be very difficult; therefore, multiple urine examinations may be required. Biopsy and/or immunologic tests may be helpful in diagnosis of these patients.
3. Both 24 hours and spot urine samples should be examined as wet mounts (after concentration using no preservatives [use saline so hatching will not occur]); the urine specimens should be collected with no preservatives. These eggs are also

occasionally recovered in stool, and so both urine and stool specimens should be examined.

4. The membrane filtration technique using Nucleopore filters can be very helpful in diagnosing infection with *S. haematbium.*
5. Patients who have been treated should be followed-up by ova and parasite examinations for up to 1 year to evaluate treatment.
6. In active infections, the eggs should contain live or mature miracidia. Examination for the confirmation of flame cell activity must be performed on fresh specimens using the wet mount or hatching test; no preservatives can be used prior to the wet mount or the hatching test.
7. During the hatching test, the light must not come too close to the surface of the water since excess heat can kill the liberated larvae. Also, the water should be examined about every 30min for up to 4 h.
8. It is important to remember that the small and less commonly seen miracidium larva of *S. haematobium* may be present in the urine; motility in unpreserved specimens could confirm this diagnosis.
9. It is also possible to see *S. haematobium* eggs in semen specimens, even when repeated urinary and fecal examinations and serologic tests are negative.

## 8. Operation and maintenance of microscope and centrifuge

### A. Microscope

Good, clean microscopes and light sources are mandatory for the examination of specimens for parasites. Organism identification depends on morphologic differences, most of which must be seen under regular microscopes at low (10×) and high (40×) power magnifications.

## 1) Nomenclature of biological microscope CX31

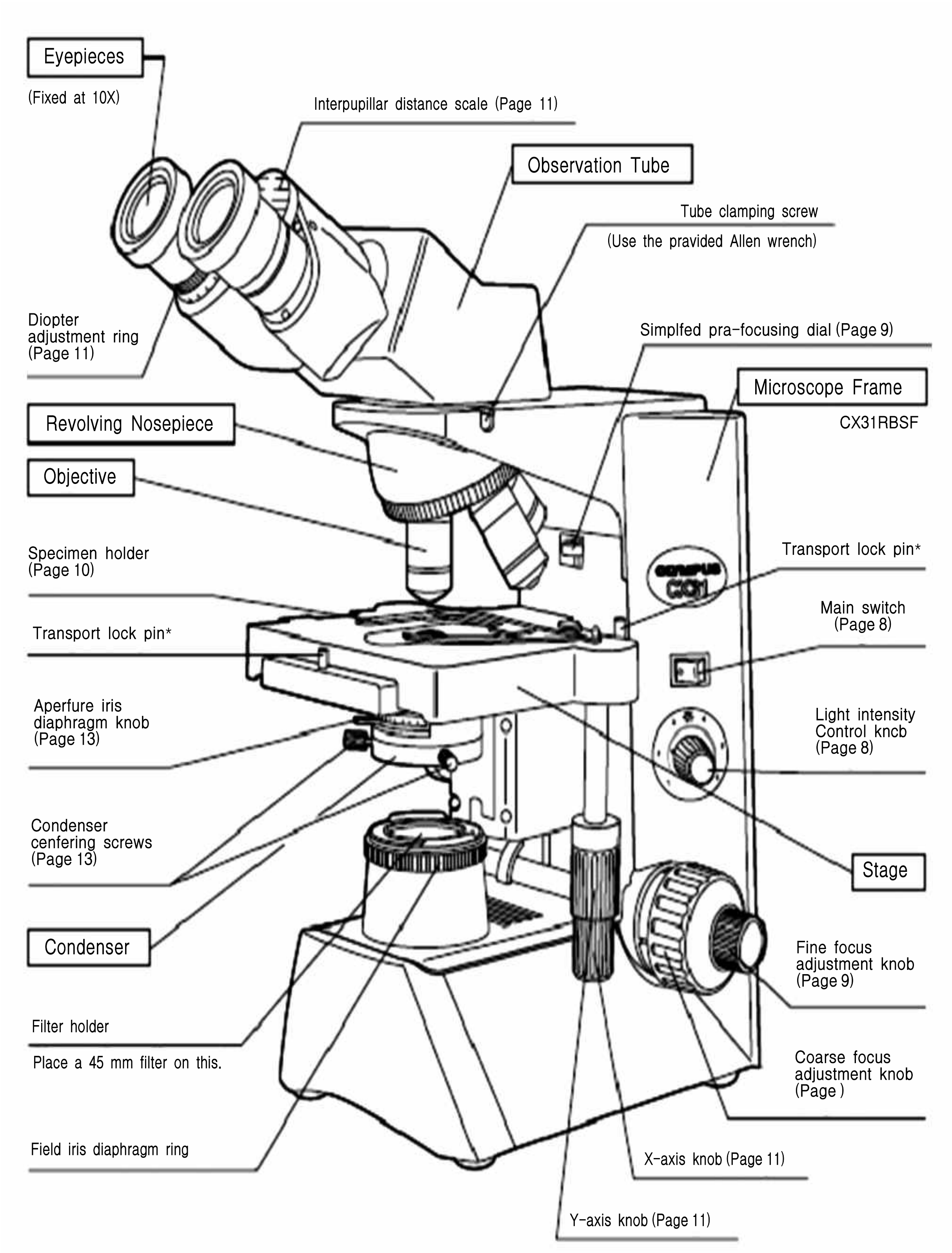

## 2) Summary of adjustment procedure of biological microscope

1

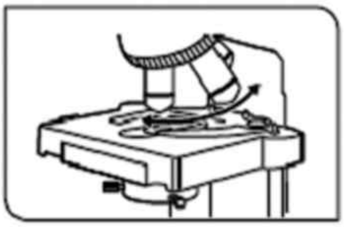

- Turn the revolving noseplece to engage the 10X objecfiva

★ Make Sure that the revolving nosepiece stops with an audible dick

2

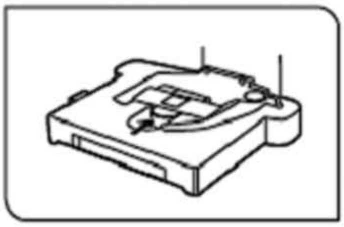

- Place a specimen on the stage. (Page 10)

3

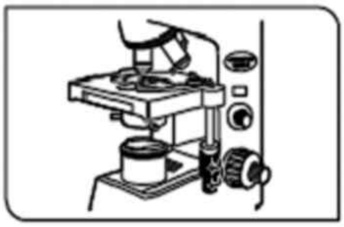

- Turn the X-axis knob and Y-axis knob to move the specimen into the light path (Page 11)

4

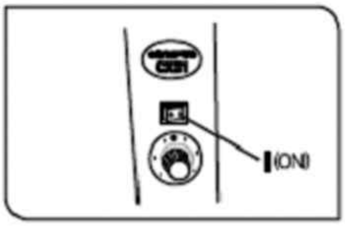


- Set the main swithch to 'I' (ON) and adjust the brightness with the light intensity knob. (Page 8)

5

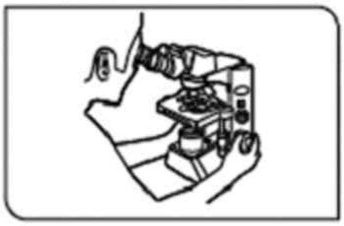

- Turn the coarse and fine adjustment knobs to bring the specimen into focus.

6

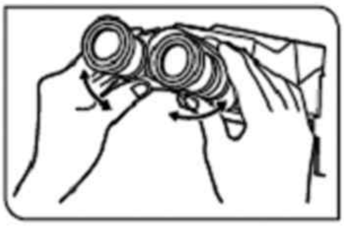

- Adjust the interpupillary distance. (Page 11)

7

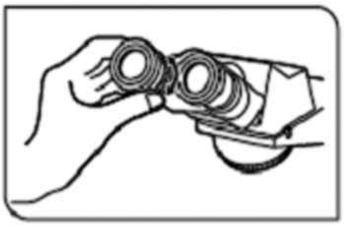

- Adjust the diopter. (Page 11)

8

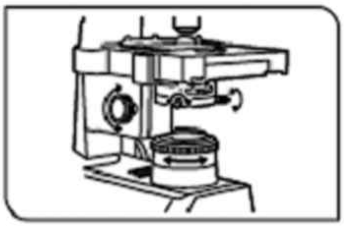

- Center the field iris diaphragm. (Page 13)

9

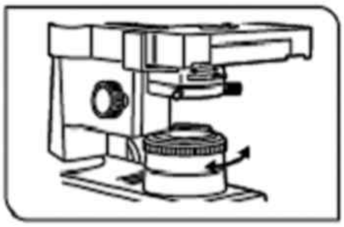

- Adjust the interpupillary distance. and field iris diaphragm. (Page 8)

10 Engage the objective to be used for ovservation in the light path, then readjust the focus.

11 Place the required filter on the filter holder.

12 Re-adjust the aperture iris diaphragm, field iris diaphragm and brightness and start observation.

### 3) Maintenance and storage

- ○ Use a camel hair brush to remove dust from all optical surfaces; remove oil and finger marks immediately from the lenses with several thicknesses of lens tissue. Single-thickness lens tissue may permit : corrosive acids from the fingers to damage the lens. Do not use any type of tissue other than lens tissue, otherwise you may scratch the lens. Use very little' pressure to prevent removal of the coatings on external surfaces of the lenses.
- ○ Clean all glass components by wiping gently with gauze. To remove fingerprints or oil smudges, wipe with gauze slightly moistened with a mixture of ether (70%) and alcohol (30%). Since solvents such as ether and alcohol are highly flammable, they must be handled carefully. Be sure to keep these chemicals away from open flames or potential sources of electrical sparks − for example, electrical equipment that is being switched on or off. Also remember to always use these chemicals only in a well-ventilated room.
- ○ Do not attempt to use organic solvents to clean the non-optical components of the microscope. To clean them, use a lint-free, soft cloth lightly moistened with a diluted neutral detergent.
- ○ Do not disassemble any part of the microscope as malfunction or damage may occur.
- ○ When storing the microscope, put it in a locker or wooden box or keep it covered with the provided dust cover
- ○ When carrying the microscope, hold put it in a locker or wooden box or keep it covered with the provided dust cover
- ○ When moving the microscope, carefully carry it with one hand under the base ① and the other hand holding at the recessed handle on the rear of the arm ② as shown in the illustration on the left.

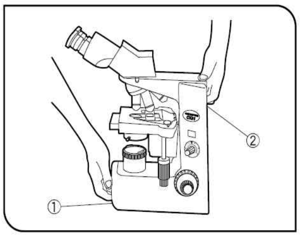

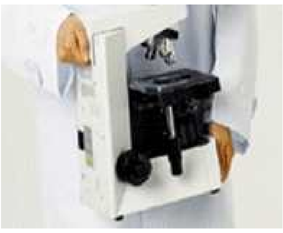
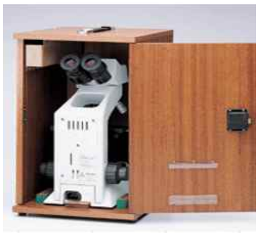
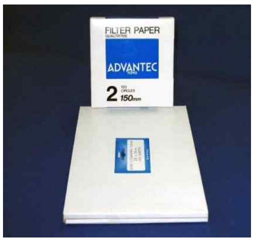

## A. Centrifuge

### 1) General Information

The Hanil HA-12 centrifuge is designed to centrifuge up to 6 individual 15 ml centrifuge tubes (adapters). It is portable and easy operation, also it has a easy speed control system.

Specifications :

Max. RPM .................................... 3,600 rpm

Max. RCF .................................... 1,811×g

Power supply ................................ 220V, 60Hz, 0.3KW

Max. capacity ................................ 6 x 15ml (max 90ml)

### 2) Precautions and hazards

- ○ Never use the centrifuge in any manner not specified in these instructions.
- ○ Never operate the centrifuge without a rotor properly attached to the shaft.
- ○ Never fill tubes while they are in the rotor. Liquid spillage may harm unit.
- ○ Never put hands in the rotor area unless the rotor is completely stopped.
- ○ Never move the centrifuge while the rotor is spinning.
- ○ Never use solvents or flammables near this or other electrical equipment.
- ○ Never centrifuge flammable, explosive or corrosive materials.
- ○ Never stay in the safety zone of 30cm around the centrifuge or deposit dangerous materials inside this zone during centrifugation.
- ○ Always load the rotor symmetrically. Each tube should be counterbalanced by another tube.
- ○ Always locate the centrifuge within easy access to an electrical outlet.
- ○ Always use only centrifuge tubes made from plastic and designed to withstand centrifugal forces of at least 2,000×g.

### 3) Operation

- ○ Operation of the centrifuge begins when the speed lever of the bottom part is moved to left (forward) side.
- ○ Individual tubes, strip tubes and/or adapters must always be loaded symmetrically to ensure proper balance. Never run the centrifuge with only one strip tube in place.
- ○ Speed (rpm) can be selected by the water level of the central steel column

from 1000 to 3000 rpm. The control of rpm corresponds to the forward/backward movement of the speed lever positioned at the bottom showing the water level of the centrifuge steel column.

○ To stop rotation, move the speed lever to zero (backward) on the bottom of the centrifuge. This will slow gradually to a complete stop. Do not attempt to remove samples until the centrifuge has come to a complete stop.

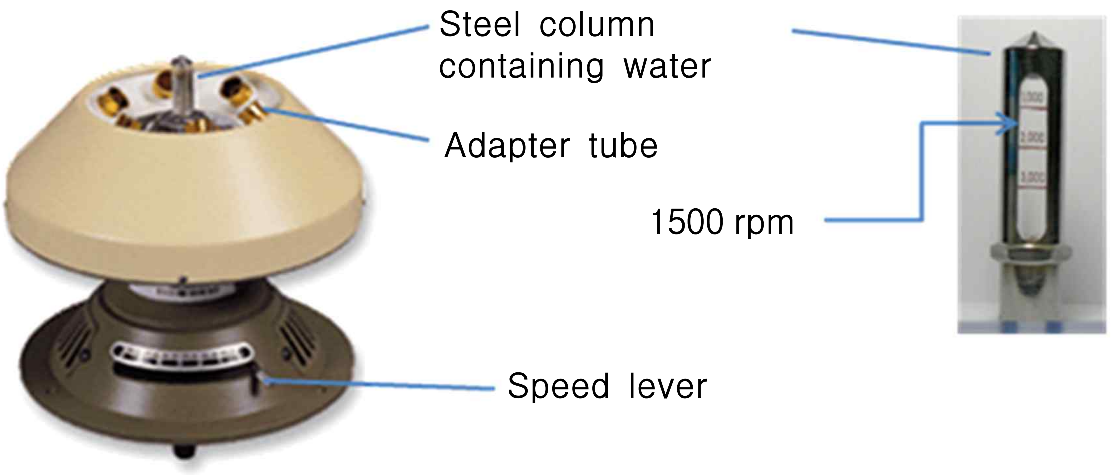


**4) Maintenance and cleaning**

○ Before each run, visually check the carrier cups, trunnions, and rotor for corrosion and cracks. If anything is found to be defective, replace it immediately or remove the equipment from service.

○ Following a breakage or spill and at least monthly, disinfect the centrifuge bowl, buckets, trunnions, and rotor with 10% household bleach or phenolic solution. Following disinfection, rinse the parts with warm water and perform a final rinse with distilled water. Thoroughly dry the parts with a clean absorbent towel to prevent corrosion. At least quarterly, brush the inside of the cups with mild warm soapy water and use fine steel wool to remove deposits; the cups should then be rinsed in distilled water and thoroughly dried.

○ To clean the centrifuge, use a damp cloth and a mild, noncorrosive detergent (pH <8). After cleaning, ensure all parts are dried thoroughly before attempting to operate the unit. Do not immerse the centrifuge in liquid or pour liquids over it.

## 9. Microscopic examination of fecal specimens

### A. The microscopic examination of the fecal material may reveal the following.

- ○ Trophozoites and cysts of intestinal protozoa
- ○ Oocysts of coccidia and spores of microsporidia
- ○ Helminth eggs and larvae
- ○ Red blood cells (RBCs), which may indicate ulceration or other hemorrhagic problems
- ○ White blood cells (WBCs), specifically polymorphonuclear leukocytes (PMNs), which may indicate inflammation
- ○ Eosinophils, which usually indicate the presence of an immune response (which may or may not be related to a parasitic infection)
- ○ Macrophages, which may be present in bacterial or parasitic infections
- ○ Charcot-Leyden crystals, which may be found when disintegrating eosinophils are present (and mayor may not be related to a parasitic infection)
- ○ Fungi *(Candida* spp.) and other yeasts and yeast-like fungi
- ○ Plant cells, pollen grains, or fungal spores, which may simulate some helminth eggs, protozoan cysts, coccidian oocysts, or microsporidial spores
- ○ Plant fibers or root or animal hairs, which may simulate helminth larvae

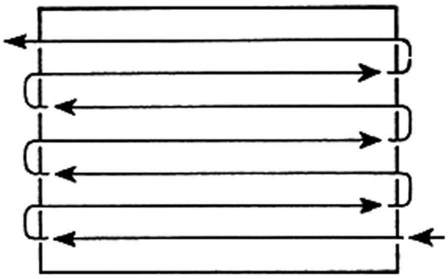

(Figure 8) Method of scanning fecal preparation with a 10x objective. Note that the entire coverslip preparation should be systematically examined with the low-power objective (10x) and low light intensity before indicating the examination is negative.[1)]

1) Source : Garcia LS. Diagnostic Medical Parasitology, 5th Ed., ASM Press, Washing DC, 2007

(Figure 9) Cysts of intestinal protozoa[2)]

1, 3. *Entamoeba histolytica*
2. *Entamoeba hartmanni*
4. *Entamoeba coli*
5. *E. coli with glycogen mass obscuring nuclei*
6. *Iodamoeba buetschlii*
7. *Endolimax nana*
8. *Blastocystis hominis*
9. *Giardia lamblia*
10. *Chilomastix mesnili*

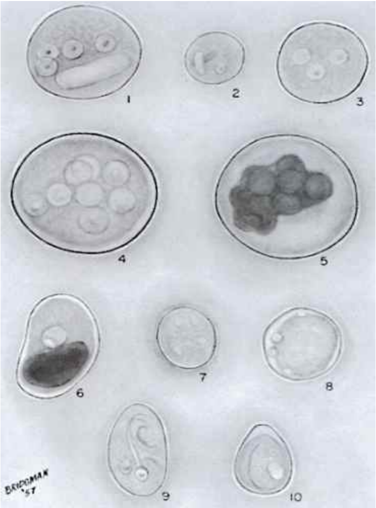

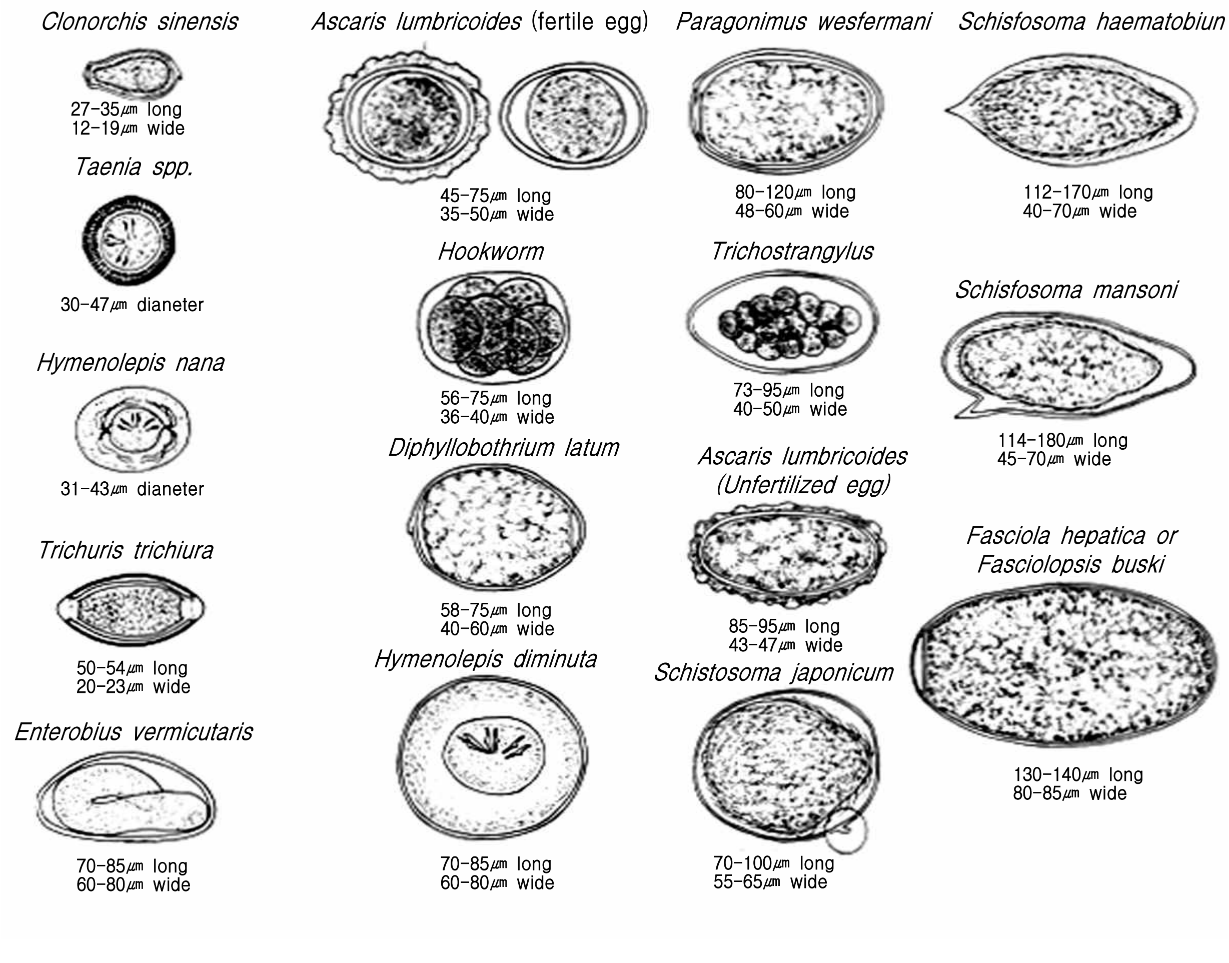


(Figure 10) Ova of various helminths[3)]

2) Source : John DT, Petri Jr WA. Markell and Voge's Medical Parasitology, 9th ed.,Sunders Elsevier, St. Louis, 2006

3) Source : Garcia LS. Diagnostic Medical Parasitology, 5th Ed., ASM Press, Washing DC, 2007

## B. Artifacts :

Although many body sites and specimens can be examined for the presence of parasites, the most difficult specimen in which to differentiate parasites from artifacts is usually fecal material. Feces consist of a number of components, including (i) undigested food residue; (ii) digestive by-products; (iii) epithelial cells, mucus, and other secretions from the digestive tract; and (iv) many types of microorganisms such as bacteria and yeasts.

Considering the ratio between fecal debris and parasites, it is not surprising that many artifacts are responsible for incorrect identifications of protozoan trophozoites and cysts and of helminth eggs and larvae. Often many yeast cells and other artifacts are confused with coccidian oocysts or microsporidial spores. Appropriate training, adherence to protocols use of quality control measures, and availability of reference materials and consultants should help minimize identification errors.

**<Table 9> Artifacts that resemble parasites**

| Artifacts in stool | Resemblance |
|---|---|
| Free-living amebae, flagellates, ciliates | Parasitic amebae, flagellates, ciliates |
| Free-living helminths, helminth eggs, or mite eggs | Helminth eggs, larvae, or adult worms |
| Yeast cells | *Cryptosporidium* spp., *Cyclospora* spp., microsporidia, helminth eggs, or protozoan cysts |
| Bacteria | Microsporidian spores |
| Fungi | Helminth egg |
| Plant material | |
| Cells | Protozoan cysts, helminth eggs |
| Root hairs | Nematode larvae |
| Pollen grains | Helminth eggs (*Ascaris* or *Taenia* spp.) |
| Pineapple juice crystals | Charcot-Leyden crystals |

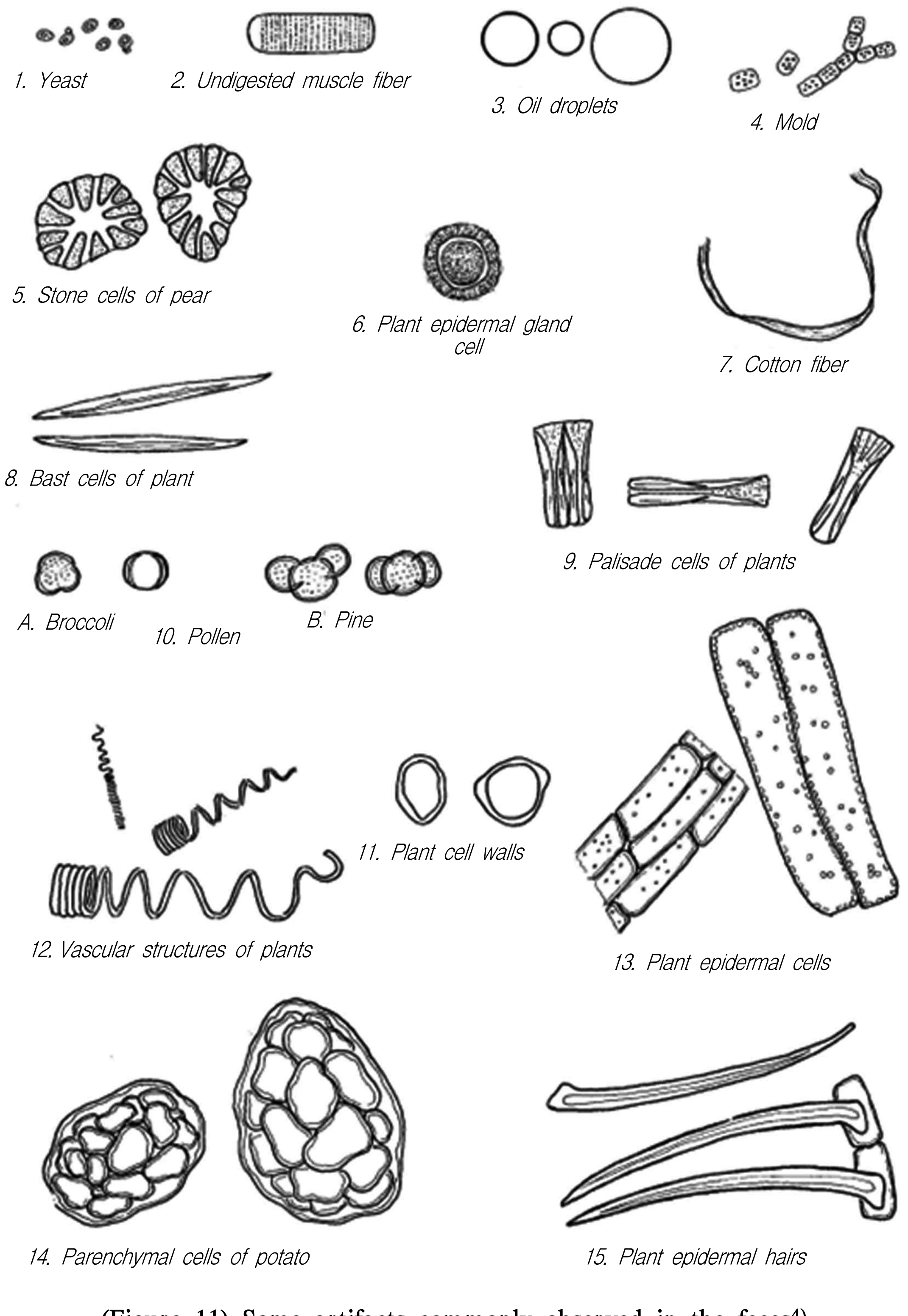


(Figure 11) Some artifacts commonly observed in the feces[4)]

4) Source : Cable RM, An illustrated laboratory Manual of Parasitology, 4th ed., Burgess Publishing Company, Minneapolis, Minn., 1958

## 10. Safety in the parasitology laboratory

A. Be careful! All materials to be received by or discarded from the laboratory must be considered potentially pathogenic.

B. Smoking, eating, or drinking in the laboratory is not permitted.

C. Do not work with uncovered open cuts or broken skin. Cover them with a Band-Aid, finger cot, or other suitable means, such as rubber or plastic gloves.

D. Develop the habit of keeping your hands away from your mouth, nose, and eyes. Wash hands well with soap before leaving the laboratory.

# Ⅵ. Chemotherapy

The strategy for controlling morbidity due to schistosomiasis is based on chemotherapy using PZQ as defined in Table 10. WHO has generated, after a long process of consensus building, a strategy whereby most adults and children infected or at risk of developing morbidity will receive the treatment they need, and a sustainable program of routine treatment if school-aged children will be introduced.

## 1. Target schools of treatment

Within each school or community, treatment will be provided on the basis of the prevalence of schistosomiasis in sentinel primary schools. In some communities, National Schistosomiasis Program in White Nile State already has this data, but in others, new surveys will be necessary to determine the prevalence of schistosome infection before chemotherapy will start. A school based treatment strategy for the control of schistosomiasis should be used in all situations, with the frequency of treatment based on the prevalence. This should be complimented by intervention in the communities, also linked to the prevalence level.

**<Table 10> WHO recommended treatment strategy for control of schistosomiasis**

<table>
<tr><th>Category</th><th>Prevalence</th><th>Action in schools*</th><th>Action in the community</th></tr>
<tr><td>Ⅰ: High</td><td>≥ 30% urinay schistosomiasis or<br>≥ 50% intestinal schistosomiasis</td><td>Treat school-age children once a year.</td><td>PZQ should be available in dispensaries and clinics for treatment of suspected cases. High-risk groups should be treated.</td></tr>
<tr><td>Ⅱ: Moderate</td><td>≥10 - < 30% urinary chistosomiasis<br>or<br>≥10 - <50% intestinal schistosomiasis</td><td>Treat school-age children once every 2 years</td><td rowspan="2">PZQ should be available in dispensaries and clinics for treatment of suspected cases.</td></tr>
<tr><td>Ⅲ: Low</td><td>< 10% infected with urinary or intestinal schistosomiasis</td><td>Treat school-age children twice during their primary schooling, for example once on entry and once on exit</td></tr>
</table>

* Enrolled and non-enrolled school-age children.

## 2. How to calculate PZQ dose of each person

The number of PZQ tablets needed to treat any individual is dependent on the weight of the patient; the recommended dose is 40 mg/kg body weight. However, weighting scales are notoriously ineffective in Africa under conditions of heat, dust and uneven ground. Therefore an alternative measure has been developed which is the dose pole (or tablet pole) : a wooden or even paper pole which is marked off in sections, four tablets.

PZQ will be given by height using a WHO PZQ dose pole. A dose pole is a straight pole or paper strip of more than 200 cm long. The door frame or wall can then be used as a dose pole. PZQ is usually a tablet of 600 mg. Generally, the tablets have a groove half way down on one surface to allow the tablet to be broken in half. The ability to divide the tablet is an important aspect of giving treatment with PZQ according to height, as described below.

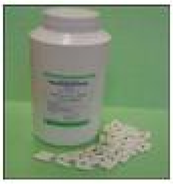

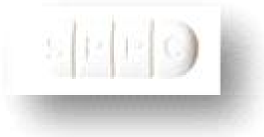

※ WHO Recommended dosages for large scale administration of PZQ

| Drug | Doses by age | |
|---|---|---|
| PZQ (600mg tablets) | 1-5 years | 6 years upward |
| | Unlikely to be infected in most situations, but it is safe to treat this age group | Use scales or the WHO PZQ dose pole |

**※ How to use a dose pole**

Each child stands next to the dose pole, with their heels against the base and standing up straight. A ruler is held on top of the child's head so that it is horizontal to the ground and one end of the ruler touches the pole making a right angle. The number of tablets is read from the pole (Figure 12).

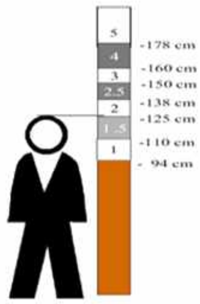


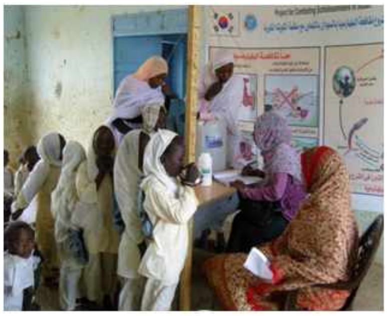

| Height of dose pole | PZQ dose |
|---|---|
| 94-109cm (37-43 inches) | 1 tablet (600mg) |
| 110-124cm (43-49 inches) | 1½ tablet(900mg) |
| 125-137cm (49-54 inches) | 2 tablet (1200mg) |
| 138-149cm (54-59 inches) | 2½ tablet (1500mg) |
| 150-159cm (59-62 inches) | 3 tablet (1800mg) |
| 160-177cm (63-69 inches) | 4 tablet (2400mg) |
| ≥178cm (≥70 inches) | 5 tablet (3000mg) |

**(Figure 12) WHO PZQ dose pole and conversion table**

### 3. Men/Women in charge who administer treatment in schools anPersd villages

The treatment team included some or all of the following : one Korean PM in Sudan, two Sudanese PM, one health worker for assistance of health education, one health worker for questionnaire survey (not always), one health worker for reception, six health workers for treatment, and one driver.

The following materials were assembled into a school or community during field survey and drug treatment.

① 1 register
② 1 wooden dose pole
③ 5 copies of each of the posters
④ 1 copy of each of the booklets
⑤ Notebook as present as many as enrolled students of each school

When you knew the target population, drugs required by each school or community could be packaged at the main Center in Rabak the day before leaving to school or village. In schools, this target population was the total enrolment of the school. In villages, this target population was the whole population of the community, and registration was made by household, including all the adults and children who lived in that household.

Both the person who delivered the drug and the recipient signed a delivery note. A copy of the delivery notes for each school and community was reported to KAHP in Korea. Drugs should be stored in a cool, dry, safe place at all times. The Korean or Sudanese PM will compile a report about the treatment coverage in each school or village as a whole. The report will include problems experienced and possible solutions.

During treatment period, all staff at the main lab in Rabak of the Project went to schools in the morning, and they administered PZQ for treatment of school children directly. They brought PZQ from the main Center in Rabak every time when they go to school for treatment. In villages, residents were treated health workers, with the help of village leaders.

Treatment could be completed in a few days, but should take no longer than 1 week for schools or villages. Korean or Sudanese PM collected unused drugs immediately after

the treatment period. If children have been registered, but were absent on the day of treatment, simply treated them as long as it was within the treatment period. If they were absent because they were sick, they should have waited until they got better to receive treatment. Staff did not give their drugs to someone else for medication of their parents or siblings.

## 4. Useful information about treatment

### A. What is the benefit of treatment for worms?

Treatment will kill most of the worms, but treated people can get re-infected if they come into contact with contaminated water after treatment. However, even if they are re-infected, the infection will be less severe. This means that, over many years, treatment may reduce the damage caused by schistosomiasis.

### B. Why is repeated treatment necessary?

Although treatment will kill most of the worms in an infected person, the environment remains contaminated. Until the sanitation in a community is improved, people in that community may continuously become re-infected. The important thing is that by repeated treatment, the number of worms in each person is kept in check and therefore serious disease may not develop. Reducing the number of worms in each person also means that fewer eggs will be released into the environment and less people will become re-infected. In time, this will reduce the number of people who require repeated treatment.

### C. Side effects of drug and their management

PZQ is a very safe drug, and it is preferably taken during a meal particularly one with a high carbohydrate content. After oral administration, over 80% of the drug is absorbed. In general, the drug is well tolerated; side effects are usually mild and transient, lasting less than 6 hours. Reactions include malaise, headaches, dizziness, abdominal discomfort, nausea, vomiting, fever, and rarely urticaria. Side effects are more likely to occur if the drug is taken on an empty stomach. If treatment is given after a meal, the side effects will be reduced. Therefore on the day of treatment you should ask parents to make sure their child is given some food. If the drugs are taken with enough water, the risks of headache are reduced.

### D. All children and adults who are to receive treatment should be told to have a heavy breakfast that day.

Treatment should be given after breakfast in the school day as possible so that children can be reduced the side effects and observed for side effects for at least 2 hours. Teachers or staffs of treatment team should check inside the mouth of each child and under the tongue to make sure the child has swallowed the tablets as some children can hide the tablets inside their mouth.

Children who feel unwell after treatment should be made to lie down in the shade and should drink plenty of clean water. If signs become serious and prolonged, you may invite a nurse or take the child to the nearest dispensary. At the dispensary, the child may be told to lie down and rest or if there is a skin rash, some antihistamines may be given. It is also important to combat any rumors that may start very quickly. The drugs are safe and only kill worms. Side effects are due to the discomfort of worms being expelled by the body.

### E. People who should not receive treatment (Exclusion criteria)

Sick children or adults should not be treated until they have fully recovered. This is done to prevent the deworming tablets being blamed for a child’s unrelated illness and thus creating misconceptions among parents and the community. Staff or teachers should not treat children with praziquantel who are less than 94 cm in height. In a school based setting, where most children start school at 6 years of age, even if a child is malnourished he/she is unlikely to be this small. Children who experience fits should not be treated in the school or community but under medical supervision in a health centre or hospital. The children and the parents should always consent to treatment. Drugs should never be given to take later.

### F. Recording the treatments given

It is vitally important to record the treatment given to each person (school children or adults) during mass treatment. This recording is essential because it allows us to monitor the program, and calculate the coverage that has been achieved. The school register could be taken from an existing enrolment register as long as it is recent. If no recent enrolment register exists, the children should be registered class by class. Similarly, for communities it is also important to register all individuals who live in a household, whether they are

present or absent on the day of registration. Drugs are calculated based on the number of adults and children registered.

# Ⅶ. Health Education for Preventing Schistosomiasis

Korean and Sudanese PM or Korean experts provided training to health workers of the Project implementation activities. Also they educated school teachers, school students and village residents for schistosomiasis and health improvement.

## 1. Training Sudanese health workers

Health workers learns a microscope manipulation method, sample collection method, stool cellophane thick smear method (Kato’s method), urine sedimentation method, urine analysis method, how to answer and collect a questionnaire, how to use a dose pole, what was the Project implementation activities, how to control schistosomiasis, how to use equipments related with the Project, and so on.

Following equipments and materials are required for training of health workers :

① space for meeting equipped with electricity
② Notebook and pens
③ Receipts for per diems, activity report and register
④ Posters and booklets related to schistosomiasis
⑤ Questionnaire for students
⑥ Evaluation and monitoring questionnaire for students and teachers
⑦ PZQ dose pole
⑧ Beam projector and screen
⑨ Centrifuge
⑩ Microscopes
⑪ Materials for cellophane thick smear method
⑫ Materials for urine sedimentation method
⑬ Urine analysis kit
⑭ Urine and stool containers
⑮ Vortex mixer

Korean and Sudanese PM or Korean experts trained health workers in case of necessary, from time to time, and individually or collectively. After the training session, there was

a free questions session with Korean and Sudanese PM or Korean experts. This training was very important to find out the exact information of schistosomiasis in the Project areas such as prevalence, infection routes, socio-behavioral patterns of school children, major target areas, etc.

## 2. Health education training for school teachers

Teachers from primary schools are gathered at one school or community education center of each Unit, and Sudanese PM or experts in field of parasitology or public health educated them about schistosomiasis and health improvements. Though this training, school teachers would be health education experts who can deliver sufficient knowledge of schistosomiasis to students. The education took 5 hours totally, and the schedule of training is summarized at Table 11. However, if there were too many teachers in one Unit to train in one day, a half of them were trained on one day and the other half on the other day.

**<Table 11> Schedule for training primary school teachers**

| Activities | Duration | Responsibility |
|---|---|---|
| 1. Take register of those present for per diems and hand out education materials | 30 minutes | Health worker |
| 2. Lecture about schistosomiasis and health improvements | 2 hours | Sudanese PM or experts |
| 3. Break, drink tea and cookie | 30 minutes | |
| 4. Watch the videos, slides, ppt files about schistosomiasis and health improvements. | 1 hour | Sudanese PM or experts |
| 5. Free questions | 40 minutes | Sudanese PM or experts |
| 6. Issuance of certificate of completion for trained teachers | 20 minutes | Issued by KAHP |

After the education session, there was a free question session with the teachers. This was designed to clear up any problems they encountered, and gave them an opportunity to raise any local issues. These issues may be important and are passed back to Korean or Sudanese PM or experts by oral or written reports.

We needed the following materials for education of school teachers :

① A space for meeting equipped with electricity

② Notebook and pens
③ Receipts for per diems and register
④ Posters and booklet related to schistosomiasis
⑤ Evaluation and monitoring questionnaire for students and teachers
⑥ Beam projector and screen
⑦ Certificate of completion of education for trained teachers
⑧ Snack and water

The teachers who completed the training are provided with the certificate for completion.

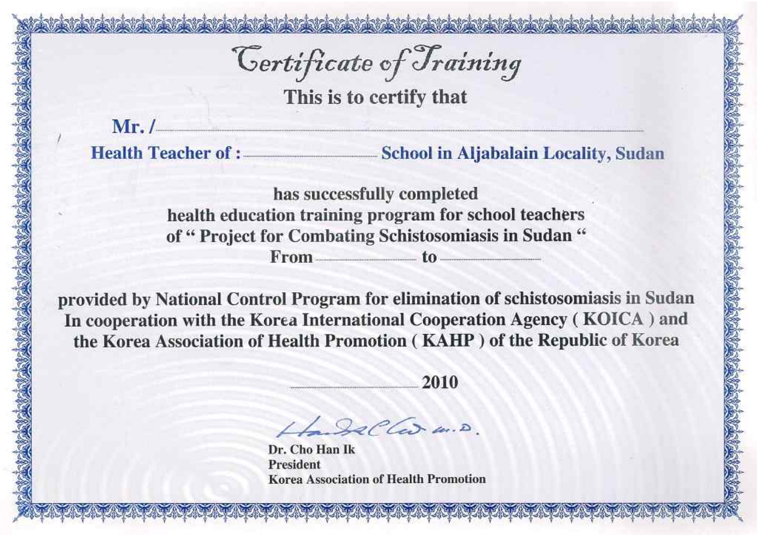
Certificate of Training
This is to certify that
Mr. /
Health Teacher of : School in Aljabalain Locality, Sudan

has successfully completed
health education training program for school teachers
of " Project for Combating Schistosomiasis in Sudan "
From to

provided by National Control Program for elimination of schistosomiasis in Sudan
In cooperation with the Korea International Cooperation Agency ( KOICA ) and
the Korea Association of Health Promotion ( KAHP ) of the Republic of Korea

2010

Dr. Cho Han Ik
President
Korea Association of Health Promotion

(Figure 13) Certificate for complementation of education

○ **Details of health education training** [5]

This training session is to provide a series of the basic knowledge like the determination of praziquantel dose following the guideline of WHO, the distribution of anthelmintics, mass chemotherapy and the treatment after the chemotherapy, with the school teachers and the health officials for the extermination of schistosomiasis, the serious waterborne disease which affects negatively to the provincial and national economics.

① **The symptoms of schistosomiasis**

The symptoms of schistosomiasis are reducing iron of the body which results in anemia, hypoalimentation which causes short stature, overall poor health,

5) Dr. Abrahim Ahmed Ali / Prof. Huaida Abidin Osman

underdevelopment and fatal complications like abnormal expansion of liver and spleen, esophagus disorder and cancer, which can result in the decrease of national productivity.

**② How to exterminate schistosomiasis and soil-transmitted parasite diseases**

To exterminate schistosomiasis and soil transmitted diseases, rapid prescription, treatment, enlightenment of health awareness and encouragement of participation in a social level, snail control, and the improvement of Health status and environment are needed.

**③ The policy of chemotherapy for schistosomiasis**

- Mass chemotherapy : When the prevalence is 40% or more, distribute anthelmintics to all residents
- Treatment by class : When the prevalence is in between 20 and 39%, distrubute anthelmintics to schoolchildren and the groups which are most exposed to the contaminated water.
- Chemotherapy for whole students : When the prevalence is in between 10 and 19%, distribute anthelmintics to whole students of the school.
- Chemotherapy for infectees : When the prevalence is under 10%, distribute anthelmintics to only infectees.

**④ The policy of chemotherapy of other parasites**

- Chemotherapy for whole students : When the prevalence is in between 50 and 70%, distribute anthelminthics to whole students of the school.
- Chemotherapy for infectees : When the prevalence is under 50%, distribute anthelminthics to only infectees.

**⑤ The anthelmintics for treating schistosomiasis**

The primary anthelminthics used in the treatment of schistosomiasis is praziquantel. It is used in tablets and 600 mg/tablet. For one person of schistosomiasis, one dose of 40 mg/kg is recommended. The praziquantel treatment effect on schistosomiasis reaches 85% and it can reduce about 95% of the eggs in the infected patients.

**⑥ The method of soil-transmitted parasites treatment**

The effective, cheap and simple treatment is albendazole or mebendazole chemotherapy and just one dosage is enough for practical treatment.

**⑦ Determination of the dosage of anthelmintics for schistosomiasis treatment**

One praziquantel tablet contains 600 mg and is applied per 15 kg. Thus, tablets were determined by dividing the body weight of the infected subjets by 15.

ex)

**Q : What is the dosage of a infected children who weighs 30 kg?**

**A : Dosage = Weight / 15 = 30 / 15 = 2 praziquantel tablets**

Dosage of praziquantel can be determined by the height of the subjected children. This method can be easily applied in the mass treatment.

<Table 12> Dosage of praziquantel according to the weight and height

| Weight(Kg) | Dosage of Praziquantel | Height(cm) |
|---|---|---|
| 15-22,5 | 1.5 | 110-125 |
| 22.5-30 | 2 | 125-138 |
| 30-37.5 | 2.5 | 138-150 |
| 37.5-45 | 3 | 150-160 |
| 45-60 | 4 | 160-178 |

**⑧ Side effects caused by the chemotherapy**

- This method is safe and has little side effects. However, very few people show symptoms like headache, light fever, stomachache, diarrhea and nausea for a short period.
- Mostly, heavily infected people tend to have more side effects than mildly infected ones.
- If the symptom is serious or lasts long, visit the near hospitals or medical centers.
- Preventing children from being in danger, make children have a meal enough before chemotherapy.

**⑨ The one who must avoid chemotherapy**

- Those who suffer from diseases like fever, diarrhea, etc. except schostosomiasis or other parasite diseases
- Women 3 months into pregnancy or under.
- Children who have got infected with pernicious malaria or anemia
- Children under 1 year of age

**⑩ The advantages of treatment for individuals**

This treatment generally promotes the health of children, protects their health from being deteriorated, stimulates their appetite and prevents many kinds of symptoms from schistosomiasis. In addition, it guarantees the normal and healthy growth of children, increases school attendance rate and helps the students focus on the class more

⑪ **The advantages of mass treatment**

Treatment for schoolchlildren is not only for themselves but also for residents because schoolchildren are most frequently exposed to the source of infection and they are influential source of the parasite infection. For this reason, decreasing the infection of schoolchildren leads to the enhancement of public health and the increase of productivity of communities.

⑫ **The required preparations before chemotherapy**

To distribute anthelminthics to schoolchildren and residents, big space like schools health centers or squares which are enough for the people to gather together in should be prepared in the center of the village(shady places, desks and chairs should be available). Then, the place should be equipped with the supplies below.

- anthelminthics (praziquantel, albendazole, mebendazole)
- drinking water (with jars, bottles and cups)
- registration note
- anthropometer or weighing scale

⑬ **The steps which should be carried out during chemotherapy**

- 1st step : Make schoolchildren and residents line stand in a queue or line up evenly
- 2nd step : Check on the required personal information like name, age, gender, etc. and write down them on the register
- 3rd step : Measure the infectee's height and weight and determine praziquantel dose.
- 4th step : Distribute praziquantel to schoolchildren and residents and check on them taking praziquantel.

⑭ **Implementation steps during or after the campaign:**

- 1st step : Inspire the health awareness of the infectees during the chemotherapy

- 2nd step : Make pieces of daily recording paper and summarize the main points of them at the end of the campaign.
- 3rd step : Deliver reports, anthelmintics and related supplies to the health centers. These materials will be delivered to the management department of the extermination program of schistosomiasis and soil-transmitted parasites.

⑮ **Main directions for the distribution of anthelminthics**

- Are you aware of the advantages of schistosomiasis treatment? That improves the nutritive condition and health of children by protecting them from the harmful effects which schistosomiasis brings about for a long period. You should question if you are not sure of the advantages.
- Explain the advantages stated above to the other teachers and parents. Make them know that extermination of schistosomiasis is important at a social level and that is for the health promotion of the children.
- For the actual extermination of parasites, the regular treatment is needed in 6-month or 1-year intervals
- Treatment of schistosomiasis and soil-transmitted diseases can be safely carried out simultaneously.
- Anthropometer is absoulutely needed because weighing scale is likely not to be prepared. Anthropometer is precise and used in determining the dose in may african countries.
- Make sure that the infectees are aware of the side effects of the chemotherapy. If an infected child suffers from side effects while himself/herself or the parents don't know about the possibility of side effects, they will not receive chemotherapy any more and doubt the importance of the chemotherapy.
- Encourage the infectees have the examination and treatment at least once a year.
- Try every means for not registered or absent children to receive treatment. Have in mind that some infectees who need the chemotherapy could be among them.

⑯ **Details to avoid**

- Pregnant or lactating women : It is safe for them to receive the treatment except for the first trimester.
- Being afraid of conducting chemotherapy for young children : It is safe for the children above the age of 1 to receive chemotherapy.

- Conducting treatment for the unregistered person : A person in charge of chemotherapy should aware of the amount of the anthelminthics which is supplied for the treatment.
- Let anyone receive the anthelminthics : Sometimes it's possible for the people not to take anthelminthics properly or to show some side effects.

## 3. Education for school students and village residents

Sudanese PM or experts in field of parasitology or public health educated students and residents in the project areas to provide sufficient knowledge on schistosomiasis and promoted self-care health awareness for prevention of re-infection. Before administration of praziquantel to school children or village residents, health education had been usually implemented at the playground or classroom in the school of project areas for 1 hour.

The following materials were assembled into a school or community during education :

① 5 copies of each of the posters
② Leaflet as many as enrolled students or total population of village
③ Receipts for per diems of lecturer
④ Beam projector and screen

### ○ Material for the education for school students and village residents

The material is to be utilized in the health education on the health awareness and the symptoms and preventive methods for schistosomiasis for students and residents to elevate the effect of the education.

**① Details of the material**

**- The infection route of schistosomiasis**

People get infected with schistosomiasis when they contact with contaminated water(in case of crossing contaminated stream, puddle, pond, river, etc. or bathing and swimming there) or urine and feces of the infectees.

**② How to prevent schistosomiasis infection**

- Raise the sense of health awareness of residents and informing them of the

dangerousness of schistosomiasis
- Do not urinate or defecate near the source of water supply
- Use latrines when urinating or defecating
- Do not cross stream, puddle, reservoir or river and do not bathe or swim there.
- Conduct repetitive chemotherapy on the endemic areas in 6-month or 1-year intervals.
- Improve the health environment
- Control the intermediate hosts(freshwater snails)

③ **How to exterminate schistosomiasis**
- Participate in the examination and the treatment actively.
- Participate in the project of water resources development(agriculture, irrigation, water storage, sugar cane
- Constitute the advisory committee for exterminating schistosomiasis

④ **Chemotherapy for schistosomiasis :**

When treating someone who get infected with schistosomiasis, praziquantel chemotherapy should be applied. This anthelmintic is safe and effective. The praziquantel dose is determined according to the weight of the infectee(40mg/1kg).

Height of the infectee can be used as the criterion instead of the weight. The dose should be determined with the decision of the doctor, the assistant or the chemotherapy expert. It is best way to take a dose after having meals.

⑤ **The kinds of schistosomiasis and its symptoms**

There are schistosomiasis haematobium, intestinal schistosomiasis, etc. as the kinds of schistosomiasis and their symptoms are like these :
- Scratching the skin as soon as being out of water
- Feeling pain when urinating
- Haematuria(especially, at about the last moment of urinating)
- Pain in the lower abdomen
- Overall poor health
- Bloody excrement

⑥ **Degeneration of the schistosomiasis symptoms are like these :**
- Physical and mental arrested development
- Liver function disorder
- The increase of spleen disorder

- Vomitting
- Esophagus malfunction
- Severe lack of blood
- Hemoptysis
- Bladder cancer

⑦ **The definition and the ecosystem of schistosomiasis**

Schistosomiasis is a kind of parasite species which lives off the inner part of blood vessels near intestines or a bladder. The female lay eggs and some of them pierce the wall of the rectum or bladder and are excreted with urine or feces. When an infectee urinates or defecates in the water, schistosomiasis contained in the urine or feces starts to lay eggs. When the eggs hatch, miracidium comes out. The miracidium live off the freshwater snail, which is the intermediate host and finally grows into cercaria, which can penetrate into the human body, piercing the skin. The cercaria finally grows into schistosomiasis in the human body.

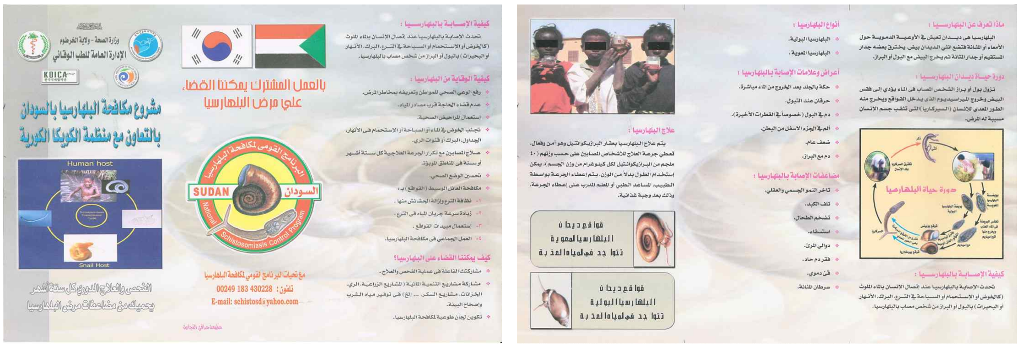


(Figure 14) Leaflet for health education(Front/Back)

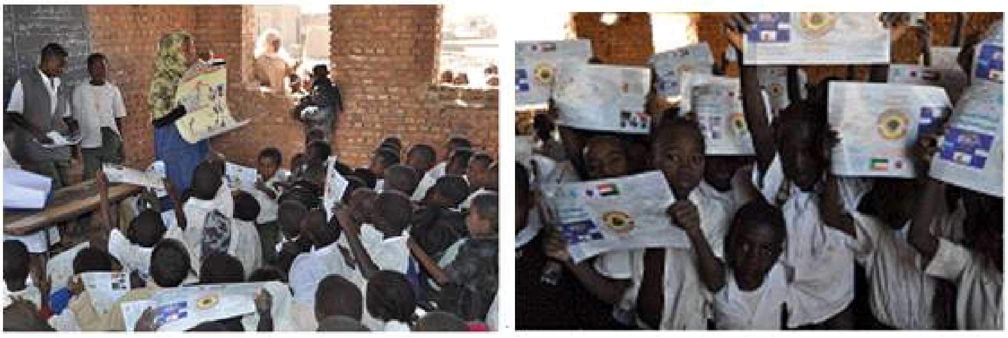

(Figure 15) The scene of health education for students using leaflets

# Ⅷ. Project for Clean Drinking Water Supply

This plan was made for discussion with Health-Medical Team of KOICA to request additional budget for drinking water supply facilities of Project for Combating Schistosomiasis in Sudan

| Category | | Contents |
|---|---|---|
| Project name | Korean | 수단 화이트나일주 알자발라인지역 알히뎁 마을 식수개발사업 |
| | English | Clean drinking water supply scheme in Al Hidaib Village,<br>Al Jabalain Locality, Sudan |
| Purpose of the project and expected contribution | | 1. Prevention of schistosome infection and reinfection after drug treatment by clean drinking water supply<br>2. Prevention of tropical diseases and waterborne diseases by blocking the infection route<br>3. Improving the health promotion and life quality of the residents and children by clean drinking water supply |
| Details of promotion | | 1. Preliminary investigation of Al Hidaib village and the nearby areas by the request of the Ministry of Health, White Nile State and the local Program Manager at January 5th through 23th, 2010.<br>2. Selection of drinking water supply area by joint meetings with National Control Program of Schistosomiasis & STH, White Nile State MOH and local project manager (PM) through comprehensive evaluation of preliminary investigation and other research materials |
| Project area | | Al Hidaib Village, Al Jabalain Locality, White Nile State, Sudan |
| Duration of the project | | From March 1st to 31st August, 2010 (for 6 months) |
| Beneficiary | | 1. Direct beneficiary : About 3,000 residents of Al Hidaib Village, White Nile State, Sudan<br>2. Indirect beneficiary : About 10,000 residents of the nearby areas in Al Hidaib Village, White Nile State, Sudan |
| Contents of the project | | 1. Construction of drinking water supply facilities<br>2. Establishment of advertisement board for Schistosomiasis Combating Project and drinking water supply scheme<br>3. Education of regional director for the facilities management and its own operating system |
| Implementing agency | | 1. Korean side : KOICA, KAHP, KOICA Schistosomiasis Control Center in White Nile State<br>2. Sudanese side : MOH and Waterworks Department of White Nile State, National Control Programe of Schistosomiasis & STH |

## 1. Outline of the Project

### A. Project name

○ Korean : 수단 화이트나일주 알자발라인지역 알 히뎁 마을 식수 개발사업

○ English : Clean drinking water supply scheme in Al Hidaib Village, Al Jabalain Locality, Sudan

### B. Duration of project : From March 1st to 31st August, 2010 (for 6 months)

### C. Purpose of project and expected contribution

#### 1) Purpose

| |
|---|
| Clean drinking water supply facility will be built in Al Hidaib Village, Al Jabalain Unit which is the highest prevalence of schistosomiasis among 6 Units of White Nile State. It will supply clean drinking water without being affected by the dry or wet seasons, and therefore it will solve the water supply shortage problem recurring annually. Also it will contribute to improving the health and life quality of the residents and children by prevention of waterborne diseases including schistosomiasis. |

#### 2) Expected contribution

○ Prevention of schistosome infection and reinfection after drug treatment by clean drinking water supply

○ Risk reduction and disease prevention of tropical diseases and waterborne diseases by blocking the infection route

○ Improving the health promotion and life quality of the residents and children

○ Contributing to the realization of MDGs

→ The Millennium Development Goals (MDGs) were developed out of the eight chapters of the United Nations, signed in September 2000. There are eight goals with 21 targets, and the problem of water shortages was dealt at MDG Goal 7, Target 10.

| [Millennium Development Goal 7 : ensure environmental sustainability] |
|---|
| Target 9 : Integrate the principles of sustainable development into country policies and programmes and reverse the loss of environmental resources |
| Target 10 : Halve, by 2015, the proportion of people without sustainable access to safe drinking water and basic sanitation |
| Target 11 : By 2020, to have achieved a significant improvement in the lives of at least 100 million slum dwellers |

## D. Details of promotion and background

### 1) Details of promotion

a) By the request of the Ministry of Health, White Nile State and the local Program Manager, preliminary investigations in Al Hidaib Village and the nearby areas were made at January 5th through 23th, 2010 and the results are as follows ;

- ○ The prevalence of schistosomiasis in Al Jabalain locality was 54.1%, which was higher infection rate than that of the other localities of White Nile State.
- ○ Among them, a lot of people live in Al Hihaib Village, however there was no clean drinking water supply facilities.

b) The result of the joint meetings with National Control Programe of Schistosomiasis & STH, White Nile State MOH and Sudanese Project Manager (Mr. Ahmed) and Korean Project Manager (Woo-Hyun Kong) was as follows :

- ○ They selected Al Hidaib Village as an area for drinking water supply project because schools and residents are relatively concentrated, and drinking water supply facilities are absolutely required.

### 2) Background

a) The canals of Nile River are well maintained due to development of sugar cane plantations in White Nile State, however the speed of water stream is slow in the canals and many water plants and reeds around the canals are providing an appropriate habitat of intermediate snails.

- ○ Therefore, the risk of schistosomiasis by the infection of the schistosome

larvae is greatly high.

b) As a severe infection area of Al Hidaib Village of Al Jabalain locality, there were no safe water supply systems, so the residents are using contaminated water of the pools and the Nile River sharing with animals.

○ In addition, residents are always exposed to the risk of diarrhea and waterborne diseases such as cholera.

## E. Project area

1) **Project area** : Al Hidaib Village of Al Jabalain Locality, White Nile State

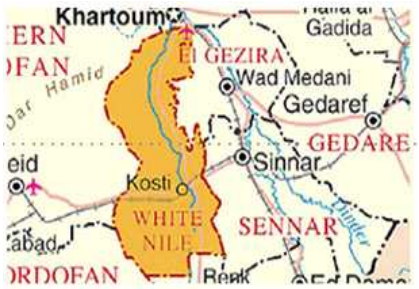

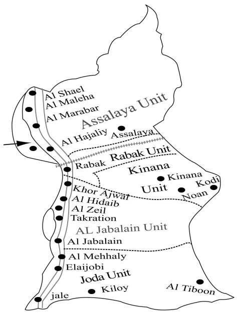


2) **Selection criteria of project area**

a) Prevalence of schistosomiasis

○ According to the survey results by National Control Programe of Schistosomiasis & STH from 2005 through 2007, the prevalence of schistosomiasis was 54.1% in Al Jabalain Unit, which was the highest one among 6 Units of White Nile State.

| Unit | Prevalence (%) | Remark |
|---|---|---|
| Kinana | 28.8 | |
| Rabak | 31.8 | |
| Assalaya | 25.5 | |
| Joda | 53.4 | |
| Jajeera abe | 31.5 | |
| Al Jabalain | 54.1 | Area for drinking water supply facilities |

b) Availability of drinking water supply facilities

○ Among 6 Units, the project area has the most hostile environments and there were no clean drinking water supply facilities.

c) Easiness of construction progress

○ The project area is located near the canals of Nile River (about 180m away), so it is easy to progress the construction of drinking water supply facilities and to maximize the effect of project advertisement due to large population.

## F. Beneficiary

1) Direct beneficiary : About 3,000 residents of Al Hidaib Village, White Nile State, Sudan
2) Indirect beneficiary : About 10,000 residents of the nearby areas from Al Hidaib Village, White Nile State, Sudan

<Table 13> Population status of the project areas.

| No | Unit | Population | Remark |
|---|---|---|---|
| 1 | Al Jabalain | 10,226 | Al Hidaib Village |
| 2 | Assalaya | 10,444 | |
| 3 | Al Jazeera Aba | 6,474 | |
| 4 | Kinana | 17,750 | |
| 5 | Joda | 4,463 | |
| 6 | Rabak | 29,258 | |
| | Total | 78,615 | |

## G. Other details

1) Expert advice : Advice of experts who recommended from KOICA if needed
   ○ Sang-Cheol Hwang, Deputy Department Head of Korea Water Resources Corporation, +82-42-629-3820

2) Budget execution: determined after discussion with KOICA
   a) It is difficult to use the itemized budget, therefore, the project for drinking water supply will be accomplished by additional agreement with KOICA after discussion with KOICA
   b) The project for drinking water supply will be implemented by addition of the reduction amount of KOICA enforcement related to this project (such as equipment, anthelmintic drugs).

## 2. Implementing plan of the project

### A. Implementing steps and role of participants

| Name of Organization | Roles |
|---|---|
| MOH of White Nile State | • Executive Director of the project in White Nile State |
| KOICA Schistosomiasis Control Center in Rabak | • Dispatched Korean PM (Woo-Hyu Kong) and Sudanese PM (Mr. Ahmed Gasm) : Practical supervision from the selection of contractor to completion of facilities<br>• Regular inspection and monitoring of facilities after completion of the project<br>• Education for facilities management to regional director |

### B. Direction and methods of the Project process

#### 1) Flow chart of implementation

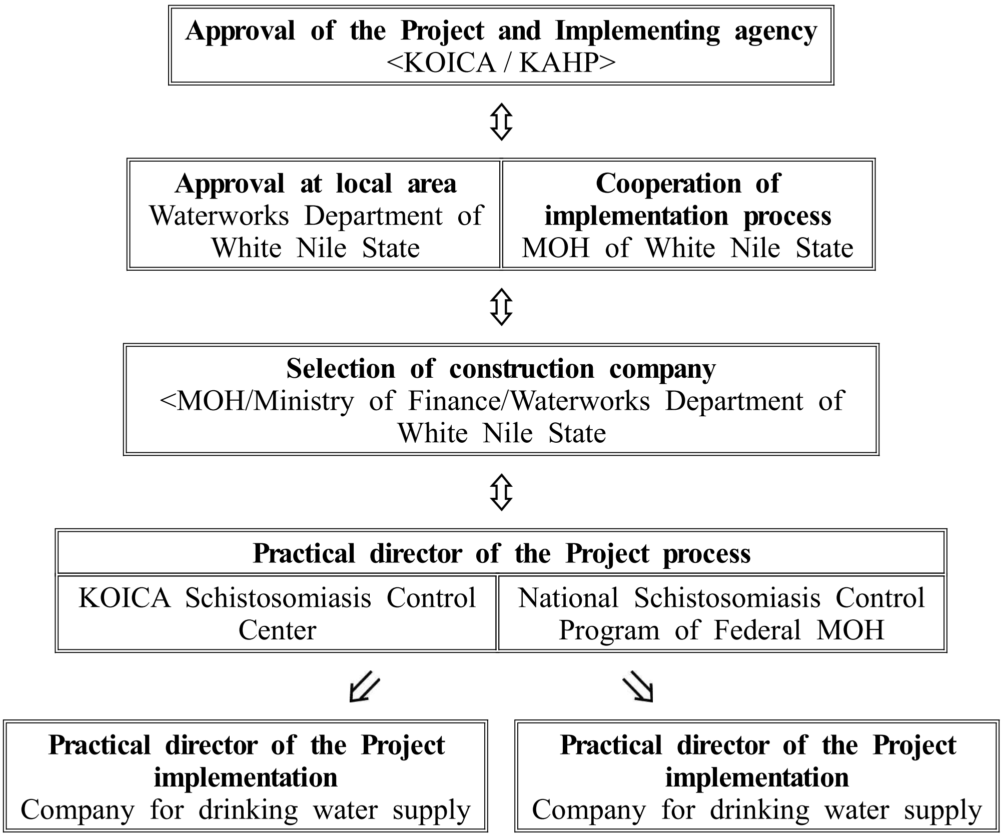


#### 2) Detailed process plan

a) Completion of drinking water supply facilities

○ Selection of contractor

- Designated : mid March to late March
- Selection method

→ Named the lowest bidder of proposed price among water development company in Sudan

○ Drinking water supply facility

- Period : April to August (5 months)
- Outline of drinking water supply facilities

○ How to construct the drinking water supply facilities?

**※ Installation of slow sand filtration system using water-related facilities (pipeline, pumps, tanks for sedimentation, filtration and storage).**

**[ Slow sand filtration method ]**

☞ Filtration method using precipitation of suspended materials in order to decrease the turbidity and chromaticity

☞ We can get safe and high quality good taste water, also it is easy to manage and does not require any drugs and power to operate.

☞ The same drinking water supply facilities (total 8 facilities) have been already settled in White Nile State, and this system is the most commonly used method in Sudan.

○ Drinking water supply system

① Drawing water through pipelines(180m) installed near the Nile River using the pump motor (Pump Motor Ⅰ)

② Store the water at sedimentation tank(the first filtration)

③ Temporary storage of purified water at "storage water tank" filtered passing through 2 filter tanks

④ Store the purified water at tanks located at 6 m in height using Pump Motor (Pump Motor Ⅱ)

⑤ Use as drinking water

○ Water purification time

→ Water tank is automatically fulfilled by automatic valve devices, if a certain

amount of clean water is reduced.

○ Water storage capacity : Up to 59,290 L

- If water usage is 10 L per person daily : about 6,000 beneficiaries
- If water usage is 20 L per person daily : about 3,000 beneficiaries

> In case of emergency situation, the world's humanitarian agencies supply one person 20 L of water minimally applied the definition of Sphere (It is an international NGO to improve the quality of assistance to people affected by disaster and improve the accountability of states and humanitarian agencies to their constituents, donors and the affected populations). However, daily water usage of the poorest oversea regions are usually spending within 10 L per person to drink, cook and wash.
>
> 【Source : Water Journal】

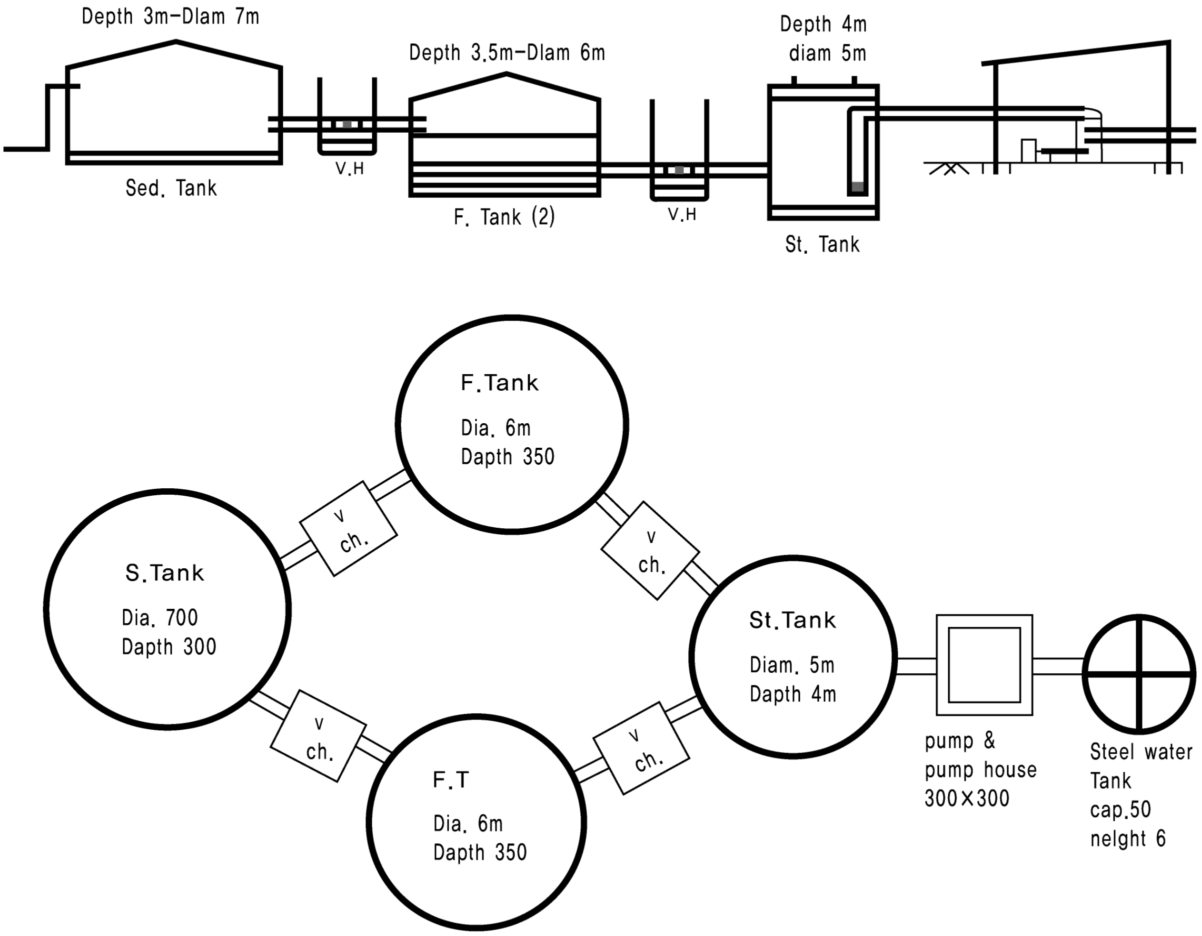


(Figure 16) Slow sand filtration system. Sed. Tank (S. Tank), sedimentation tank; V.H. (V Ch.), Valves in the channel; F. tank (F.T), filtration tank; St. tank, storage tank.

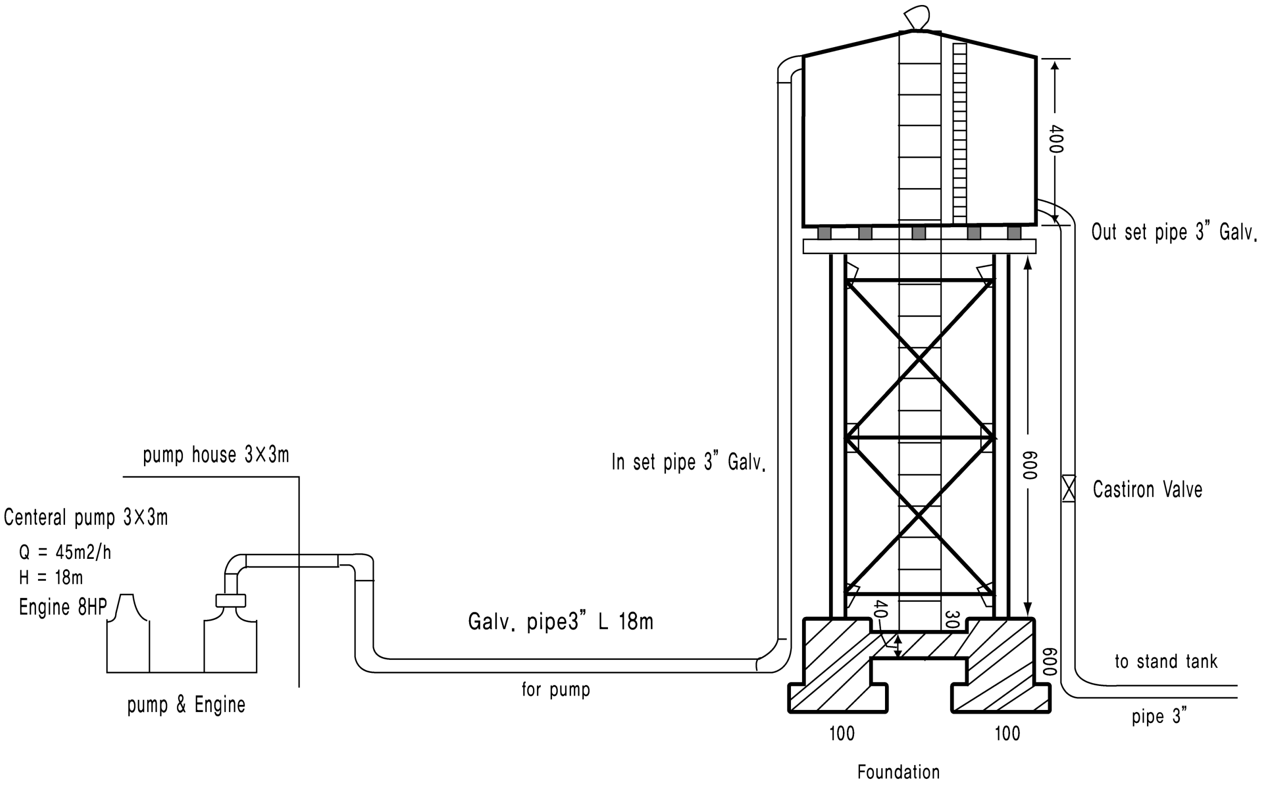


(Figure 17) Blueprint of clean water supply facility

○ How to manage the facilities?

① Gravel, sand, charcoal and electricity are required to operate continually after completion of the facilities.

② According to the manager's discretion, the gravel, sand and charcoals as a filter should be cleaned about 4 times annually depending on the degree of contamination

③ The cost of facility maintenance plans to cover by receiving the low water fees.

④ Maintenance manual for drinking water supply facilities should be prepared and be kept near the facility to cope with machinery failure.

⑤ The facility management chart must be prepared and the facility must be monitored according to the manual periodically.

b) Establishment of advertisement board for publicity

○ Size : immobile steel pole, 75cm × 110cm

○ Contents : schistosomiasis transmission, clinical sign & symptom, prevention method, etc.

○ Basic draft : Both national flags, logos of air-ticket solidarity contribution should be included in the billboard

→ Promote more than one time at the Sudan mass media (TV, newspapers) near the completion of the facilities

c) Training of facility management

○ Instructor : Officials of drinking water development company and Project Manager in Sudan

○ Education target : chief of town and local health officials

○ Contents : operation principles and management methods of drinking water supply facilities.

## 3. Period of detailed process

| Items | February | March | April | May | June | July | August | Remarks |
|---|---|---|---|---|---|---|---|---|
| Selection of target area | Preliminary investigation | | | | | | | |
| Selection of company | | | | | | | | |
| Start of construction | | | | | | | | |
| Duration of construction | | | | | | | | |
| Examination of water quality and completion | | | | | | | | |
| Monitoring and evaluation | | | | | | | | |
| Business discussion | | | Approval | | | | | Including the administrative affairs |

## 4. Evaluation and monitoring plan

○ Officials of MOH of White Nile State, dispatched Korean PM and Sudanese PM will carry out them after completion of the facility.

### A. Methods

1) Quarterly monitoring at schistosomiasis control center in Sudan

2) Joint monitoring with MOH and Waterworks Department of White Nile State, two

times annually

B. Assessment

1) Construction of drinking water supply facilities

a) Is the construction of the drinking water supply facilities completed?

b) Is the water stored in tank through a filtration process safe to drink?

c) Is the drinking water supply facilities located within 30 minutes for drawing water (20 minutes round trip, pumping 10 minutes) or within 250 meters in distance from the center of town?

d) Are the beneficiaries satisfied with drinking water supply facilities?

2) Maintenance and management of drinking water supply facilities

a) Is the drinking water supply facility protected from trespassing?

b) Is the water quality test conducted regularly?

c) If contamination breaks out, will the result of processing it be recorded ?

d) Are the management records such as regular examination or trouble of drinking water supply facilities kept well?

e) Is the drinking water supply facilities maintained in normal function after completion of it?

f) Is the basic operating costs for maintenance of drinking water supply facilities supplied from the community itself?

g) Do the officials keep the operation record and monitor its operating status regularly?

## 5. Self-management plan after completion of the project

### A. Training for facility management

1) Training of the adequate person among village officers for facilities control and maintenance

2) The cost of facility repair and maintenance plans will be covered by the low water fees.

- There are some cases that operating expenses are covered by selling the water to donkey-water seller.

B. Regular monitoring

1) Inspection is quarterly conducted by KOICA Schistosomiasis Control Center in White Nile State, Sudan.

2) Inspection and examination are conducted 2 times annually by MOH of White Nile State, Sudan.

# Ⅸ. How to Monitor and Evaluate the Project Implementation

The Project for Combating Schistosomiasis in Sudan was officially launched 1) to reduce the prevalence of schistosomiasis significantly by the end of 2011, 2) to improve the awareness and practice to prevent the schistosomiasis, 3) to provide the drugs and medical equipments for control of schistosomiasis, and 4) to reinforce the environmental infrastructure for the prevention of schistosomiasis in the pilot areas. Therefore, the monitoring and evaluation of the Project should be related with the primary objectives of the Project.

Data were collected by Korean and Sudanese PM, health workers of main Center in Rabak, and the results of the Project implementation activities will be checked by monitoring and evaluation team. Monitoring and evaluation team should include some or all of the Korean and Sudanese experts, officials of Federal and White Nile State MOH of Sudan. They were monitoring the indicators in schools and communities. Sometimes, they collected registers and administer a questionnaire to a small group of children or adults. They visited selected target schools and villages and sampled for evaluation after treatment.

## 1. Collection of data

A. To monitor and evaluate the Project, Korean and Sudanese experts or Sudanese government officials will carry out the activities below.

1) talk to teachers and village leaders to get their opinion about the schistosomiasis control project, post-treatment effects, discomfort after treatment, and so on.
2) visit sampled schools and villages after treatment.
3) check records at these schools and communities to ensure treatment was done appropriately.
4) check that equipments, supplies and drugs were delivered, and if they were useful and appropriate.
5) check that health education messages were delivered and the messages were appropriate.

B. The useful monitoring and evaluation indicators of this Project are followings :

1) Number of schools participating in the Project

2) Percentage of schools participating in the Project

3) Number of trained teachers

4) Number of tablets (anthelmintics) distributed during activities

5) Number of school-age children treated (enrolled, nonenrolled) and other high risk groups

6) Prevalence of any haematuria or urinary schistosome infections

7) Proportion of visible haematuria or ‘heavy intensity’ urinary schistosome infection

8) Proportion of children with defined clinical signs or symptoms

9) Number of students (village residents) participating in at least one health education activity

10) Percentage of students (village residents) participating in at least one health education activity

11) Number of awareness questionnaire for health education

<Table 14> List of monitoring and evaluation of the Project for Combating Schistosomiasis in Sudan 2009-2011

| Contents | | Goal | % | Outcome indicator | Remarks |
|---|---|---|---|---|---|
| Dispatch of PM | Dispatch of Korean PM to Sudan | 1 person | 10 | | |
| | Dispatch of Korean experts to Sudan | 5 times | 10 | | |
| | Dispatch of Korean inspection teatm to Sudan | 2 times | | | |
| Field survey | Prevalence of schistosomiasis | To reduce more than 50% of initial prevalence after the chemotherapy[6] | 20 | | |
| Treatment | Praziquantel (600 mg) | 400,000 persons | 10 | | |
| | Albendazole (400 mg) | 200,000 persons | | | |
| Health education | Development of health education materials | 7 kinds | 5 | | |
| | Health education to students and residents | 50 times | 5 | | |
| | Health education training to teachers | 150 persons | 5 | | |
| | Establishment of billboard | 20 ea | 2 | | |
| Supply of equipment and supplies | Medical equipment | 6 kinds 23 ea | 7.5 | | KOICA |
| | Supplies | 7 kinds | | | |
| Supply of anthelminthics | Anthelmintics | quantity to treat 550,000 persons | 7.5 | | KOICA |
| Workshop | Monitoring and evaluation | 3 times | 3 | | |
| Development of guideline | Schistosomiasis control guideline | 1 times | 5 | | |
| Supply of drinking water | Construction of clean drinking water supply | 1 ea | 5 | | |
| Promotion of project | Sudan | 2 times | 2.5 | | |
| | Korea | 6 times | 2.5 | | |

6) Aims at the decrease of prevalence more than 50% in the monitoring survey 6 months after the chemotherapy in the baseline survey

## 2. Monitoring and evaluation questionnaire for students and teachers

○ The aims of the questionnaire are like these.

- Check the effects of health education for teachers or students.
- Reinforce each teacher's knowledge about schistosomiasis and health promotion.

## 3. Analysis of data

Differences in the prevalence and intensities of parasite infection at specific points in time can provide a measure of the impact of the intervention(s). It is critical that there is a baseline measurement of prevalence and intensity of infection. Crude differences in prevalence can be directly calculated as the difference between the prevalence obtained at the evaluation timepoint and the prevalence obtained at baseline from the same age group usually.

### A. Major indicators

The relevant parameters to guide the decision-making process for the control of schistosomiasis are like these.

1) Prevalence of infections

a) gives information on the number of infected people in a population

b) The following formula is used to calculate the prevalence of infection in a community:

$$\circ\ \text{Prevalence} = \frac{\text{Number of subjects testing positive}}{\text{Number of subjects investigated}} \times 100$$

2) Intensity of infections

a) gives information on the severity of an infection.

b) The intensity of infection at community level could be expressed in these way.

○ mean EPG

The units for measuring the intensity of infection at the individual level are “eggs per gram of faeces” (EPG) and the “eggs for 10 ml of urine” (when using reagent strips, the intensity can only be estimated approximately).

○ classes of intensity

The thresholds proposed for use by a WHO Expert Committee in 1987 for the

classes of intensity for each helminth in stools are the following, but flexibility in setting threshold may be necessary.

<Table 15> The criterion of parasite infection intensity by the number of eggs

| | light intensity infections | moderate intensity infections | heavy intensity infections |
|---|---|---|---|
| *A. lumbricoides* | 1-4,999 epg | 5,000-49,999 epg | •50,000 |
| *T. trichiura* | 1- 999 epg | 1,000-9,999 epg | •10,000 |
| Hookworms | 1-1,999 epg | 2,000-3,999 epg | • 4,000 |
| *S. mansoni*<br>*S. japonicum* | 1- 99 epg | 100- 399 epg | • 400 |

The classes of intensity proposed for urinary schistosomiasis are the following:

| | light intensity infections | heavy intensity infections |
|---|---|---|
| *S. haematobium* | < 50 egg / 10ml | •50 egg / 10ml or visible haematuria |

**B. Other indicators**

1) Process indicators

a) Training

b) Drug distribution

c) Drug coverage

d) Health education activities

2) Morbidity indicators

Proportion with clinical signs or symptoms

○ Knowledge-Attitude-Practice (KAP)

Where it has been decided to assess whether a change in knowledge, a change in attitude or a change in practice (behavior) has followed from control activities, a KAP study can also be conducted. This type of study is based on interviews and /or questionnaires of a representative sample of the study population, usually at two or more points in time. Changes are assessed by comparing results between time points. KAP results should be linked to the health education component of the control programme.

# Ⅹ. How to Manage Project Implementation

The Project for Combating Schistosomiasis in Sudan was officially launched in Aljabalain locality, White Nile State, in August 2009. KOICA provides grant aid within the budget limit of 1,188 million Korean Won (equivalent to 1.2 million US dollars) for the Project, and the Federal MOH of the Republic of Sudan appropriates a sufficient amount from its budget to cover the expenses required for fulfilling the successful implementation of the Project. This project is managed by KAHP as a Project Management Consultant (PMC). At the national level, implementation is managed by a KOICA in Korean side and Federal MOH in Sudanese side. KOICA team purchases drugs, equipments and supplies.

Korean experts (Korean PM in Korea) are responsible for laboratory settlement in Sudan, consultation of implementation activities, project monitoring and evaluation, joint workshop with Sudanese experts or government officials. At the community level, implementation is managed by Korean PM in Sudan headed by KAHP in Korea, assisted by Sudanese employees and health workers of White Nile MOH of Sudan. They work with the management of laboratory, field survey and treatment, health education, development of health education materials, and supply of drinking water.

KAHP international corporation team works closely with the KOICA, Federal and White Nile MOH of Sudan, National Control Program of Schistosomiasis & STH, and manages this Project generally.

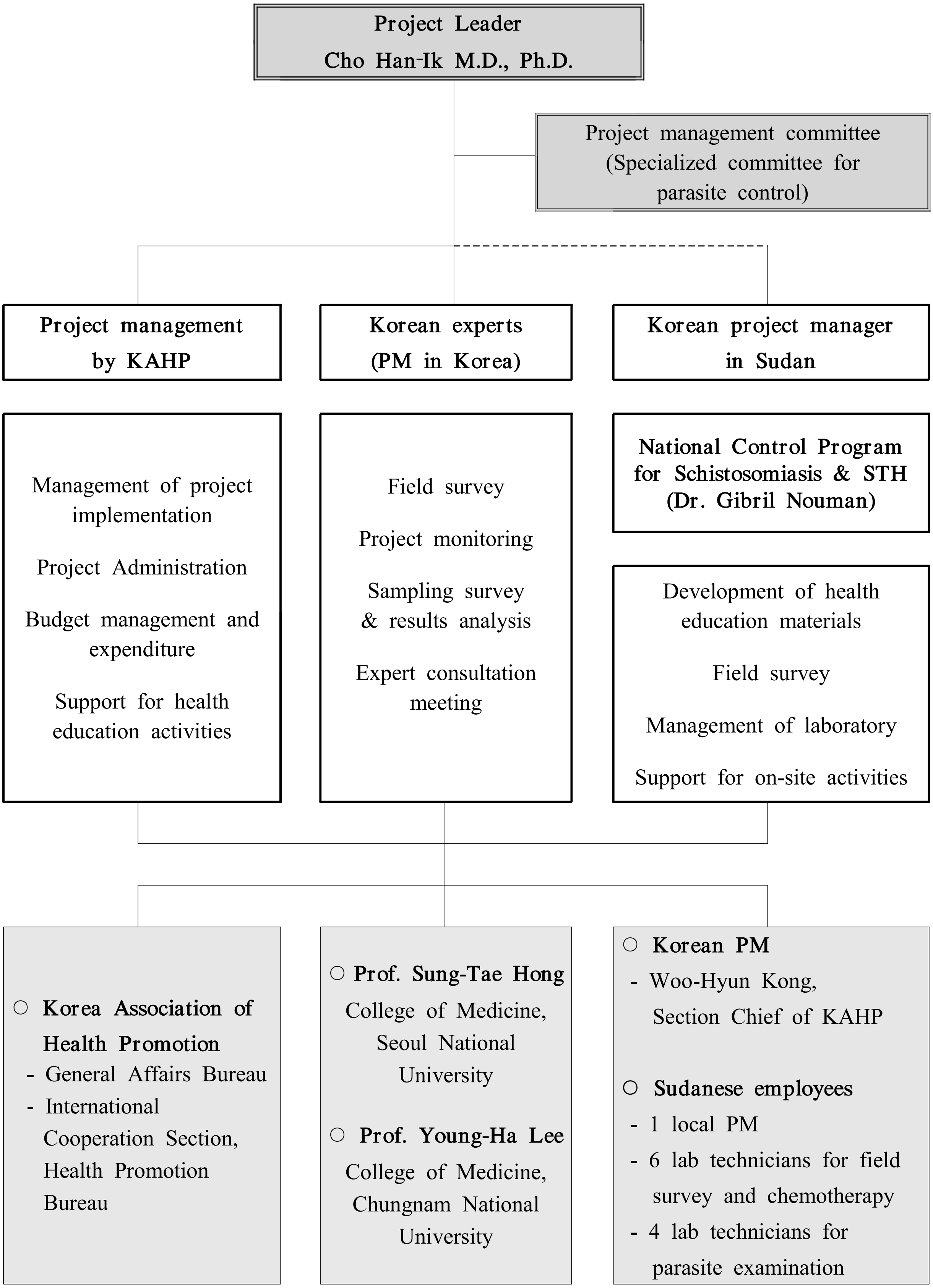


(Figure 17) The organization of the Project for Combating Schistosomiasis in Sudan

# XI. Snail Control by Molluscicide, Niclosamide

## 1. Selection of target areas

- The area for treatment of molluscicide is selected by prevalence of schistosomiasis and snail sampling.
- Frequently water-contact sites of the residents are good point for treatment of molluscicide.
- For baseline investigations, snail samplings are collected from canal or pond of the targeted areas.

## 2. Public relations

### A. Community consent

1) Community consent is obtained from the relevant health, administrative and village authorities,
2) During a preliminary visit, health, administrative and village authorities are contacted and explained the goal of the project.
3) The ethical clearance is obtained from the Federal Ministry of Health and the ethical committee of the Ministry of Health of White Nile State.
4) All study procedures followed are in accordance with the Helsinki Declaration of 1975, as revised in 1983.

### B. Education of population in treated areas

- Since the ultimate aim of using molluscicides is the prevention of bilharziasis, snail control must be understood and supported by the people of the area. Efforts should be made to explain the nature of the life-cycle of the parasite and the method of transmission: the effect on the health of the individual, and the economic impact of the disease on the community. The local population should be made aware also of the necessity of obviating pollution of the water supply and avoiding exposure to water containing schistosome cercariae.

- Radio and television broadcasts and cinema films have played effective roles in advising the public about molluscicinding programs, and precautions desirable in the use of treated water.

## 3. Selection of molluscicide

### A. Characteristics of a good molluscicide:

1) it should have low toxicity for many domestic animals, game an fish; the chemical should kill not only the snails but their eggs;
2) it should be active at very low concentrations since this reduces transport costs significantly;
3) it should have a low cost so that it can be employed economically;
4) it should be reasonably safe in the hands of trained but relatively unsophisticated people;
5) it should be usable with simple, durable equipment;
6) it should be stable in storage and in the habitat after use;
7) tests should be available to measure the concentrations used in the field

### B. General information of niclosamide

○ Niclosamide is a highly effective, WHO recommended, molluscicide, and kills a huge variety of snails, cestodes and cercariae. Niclosamide is widely used in programs to control the vector of Schistosoma species and in recent years it has been increasingly applied to control of *Pomacea* spp. (Golden Apple Snail-GAS) in paddy fields in South-East Asia and in some Caribbean countries.

○ Niclosamide is quickly metabolised in the water and safe to use, safe for applicator and does not possess any long-term effect in the environment. The substance is not irritating to snails, and leads therefore to a complete eradication in treated ponds/paddy fields.

○ Niclosamide does not exhibit any phytotoxic effects on neither the target plant rice nor on non-target plants such as algae, other water plants or zoo- and phytoplankton.

○ Niclosamide has a potential to acutely affect aquatic organisms such as fish,

frogs, tadpoles and other amphibian animals, so the application of niclosamide has a deleterious impact on the environment and biodiversity. Once mollusciciding cease, snail populations have the ability to breed and reach pre-control levels in a matter of two to three years.

- Due to its mode of action niclosamide affects the respiratory system of the target organisms by disturbing the oxidative processes in the respiratory chain (ATP-synthesis). In vitro studies show that niclosamide inhibits the rat's liver mitochondrial synthesis of ATP. Furthermore, niclosamide affects carbohydrate metabolism by inhibiting glucose uptake, leading finally to exhaustion of the energy reserves.
- Niclosamide is now the molluscicide of choice and is virtually the sole commercially available compound at the present time.
- This compound has been used extensively in Zimbabwe, Brazil, St. Lucia, Egypt, Sudan and elsewhere, in attempts to reduce levels of transmission, and hence to reduce levels of prevalence and intensity of infection in the human population.

## C. Properties of niclosamide

| **Ⅰ. Active ingredient :** 5,2 Dichloro-4-nitro-salicylic-anilide | |
|---|---|
| 1. Form | Crystalline powder |
| 2. Boiling point | (melting point 212-224℃) |
| 3. Solubility $H_2O$ | 10 p.p.m. |
| 4. Effect on metals | Non-corrosive |
| 5. Stability | Stable |
| **II. Effects on Plants and Animals** | |
| 1. Molluscicidal (Laboratory) | 4-8 p.p.m × hr (Various snails) |
| 2. Mode of action | Respiratory poison |
| 3. Ovicidal | Same as for adults |
| 4. Cercaricidal | At lower than molluscicidal concentrations |
| 5. Piscicidal | At molluscicidal concentrations |
| 6. Phytotoxic | Slightly for submerged plants. No effect on crops at 10 times molluscicidal concentration |
| 7. Mice, single oral | 2 g/kg "tolerated" |
| 8. Rats, single oral | 5 g/kg "tolerated" |
| 9 Rabbit, single oral | 5 g/kg "tolerated" |

| | |
|---|---|
| 10. Dog | 250×0.1 g/kg : “no effect" |
| 11. Cow, continuous oral | 93×10 mg/kg : “no effect" |
| 12. Man | 2×1 g/60kg : “no side effects" |
| **III. Application in field** | |
| 1. Equipment | Sprayers, dusters and automatic dispensers |
| 2. Methods of application | Spray or drip, granules, briquettes |
| 3. Amount | 4-8 p.p.m. × hr |
| 4. pH | 6-8 optimum rate |
| 5. Turbidity | May protect against ultra-violet light |

## 4. Methods of employing molluscicide

### A. Calculation of dosage for flowing water

- The calculation of the proper amount and application time of a molluscicide for flowing water actually involves four steps. Although most of the necessary information for these calculations can be determined before the application, the last step must be performed in the field. These steps are as follows:

1) *Step* 1. *Standard unit volume of water.* One gram of chemical in a cubic metre of water produces 1 p.p.m. The amount of water passing a given point in one hour in a stream or a canal with a flow of 1 $m^3$/sec. would be 3600 $m^3$ and this would require 3600 g or 3.6 kg of the chemical to give 1 p.p.m. for the total volume.

2) *Step* 2. *P.p.m.-hr rated potency.* This relationship is first determined experimentally in the laboratory and then under field conditions. For example, if it is found that 10 hours of exposure to a concentration of 5 p.p.m. is required to kill the snails, the " rated potency" is 50 p.p.m.-hr.

3) *Step* 3. *Determination of the total amount of a formulation required to treat the total volume at a standard rate of flow, 1 $m^3$/sec.* The initial determinations of p.p.m.-hr are usually made on the basis of the actual amount of active ingredient present. When a compound has reached the stage where it is ready for field use, however, it usually has been mixed with other materials to give it certain qualities which make it easier to apply, more effective, and

so on. Therefore, if the formulation used in a control program is not relatively pure, it is necessary to take this into account. For example, if one has to determine the amount of an 80% formulation of each of the two chemicals, the following calculations would be made.

*Bayluscide* : If 3.6 kg is again required to treat the standard volume, the determined p.p.m.-hr is 7, and an 80% formulation is used, the total amount of material required would be determined as follows :

3.6 x 7 x 100/80 = 31.5 kg

4) *Step* 4. *Determination of amount of material to be applied in a given flowing-water habitat.* Ordinarily only one molluscide and probably only one formulation will commonly be used in a given area. To determine the amount of formulation required in each flowing-water habitat, first calculate the volume of flow. The following examples, using the data given on the preceding page, are for two different volumes of flow.

*Bayluscide* : If an 80% formulation of this compound is to be used in the habitats mentioned, but the chosen treatment time is to be 9 hours, the following calculations would be made:

With a flow of 2.3 $m^3$/see:

31.5 x 2.3 = 72.45 kg of the formulation required

72.45/9 = 8.05 kg/hr.

With a flow of 0.15 $m^3$/see: 31. 5 x 0.15 = 4.725 kg of the formulation required 4.725/ 9 = 0.525 kg/hr.

B. Calculation of dosage for stationary water

○ In still waters the chemical is not as rapidly dissipated as in running water, but will remain active for a much longer time, enables an initial lower dilution to be employed. In most instances, the lower dilutions mentioned for each

chemical will suffice for providing effective concentrations for stationary water. For measuring dosage for stationary waters, the first three steps given may be employed. The volume of water can be estimated in cubic metres or other units by taking the product of the average width X the average length X the average depth.

- Large ponds and lakes present a special problem. It is often neither necessary nor feasible to treat the entire volume of water. Snail hosts are usually concentrated near the margins and may only occur along certain portions of the shore-line. It is customary in such circumstances to apply molluscicides only to the infested parts of the pond or lake.

**C. Calculation of dosage for moist-soil habitats**

- The amphibious snail hosts *(Oncomelania* spp.) of *S. japonicum* frequently must be attacked when they are out of the water habitat. Some of aquatic snail hosts are markedly resistant to desiccation and may survive the dry season and repopulate the area after the rains begin. It is difficult, if not impossible, to destroy snail hosts under completely dry conditions.
- The molluscicide may be applied as a spray, or, under certain circumstances, dusts 'might possibly be effective.

**D. Application in general**

- It is probable that the most effective use of molluscicides is in small water bodies where the transmission foci are well defined in both geographical terms and periods of active transmission.
- When snails are found in marginal vegetation, 2-3 ppm is applied along the shore (to various distances from the shore) from 10-40 m upstream of the water contact sites to 10 m downstream.
- In case of snails found in low submerged vegetation, the chemical was applied at 2-4 $g/m^2$ directly between the plants.
- In the pond, a wettable powder with niclosamide at 70% (w/w) is applied to the ponds at 0.5 $g/m^3$ (0.5 ppm).

The weight of niclosamide required for each application is derived from

estimates of pool volumes assuming that the contours of pools approximated a segment of a sphere. The volume of water in each pond is determined based on a paced measurement of circumference from a standard scale

○ In canal, niclosamide treatment is effected at the river intakes. Two 44-UK-gal drums are connected in parallel to one exit pipe with a nozzle to give a flow of 280 ml/minute (88 UK-gal/24 h). A nozzle of size 9 was large enough to avoid clogging. Molluscicide is applied from constant flow drip cans to the stream sections at a target dose of 8 mg/litre niclosmide every four weeks. The drums are then topped up with water (sieved to prevent nozzle-blocking by grass etc.) and the chemical is allowed to flow out. A steady emission rate is maintained by using the constant-head principle.

## 5. Evaluation

○ The efficacy of mollluscide on snail population is assessed by counting the average number of live snail count in standard 40 dip scoops. Snails are determined to be alive or dead, and separated by species and counted.

○ Investigation of fish, frogs, and tadpole kill is adapted from standard guidelines (APHA 1985). The kill is determined based on the number of dead tadpole, fish or frog per 2000 adjusted paces of the pond circumference as nil (0), minor (1-100 dead), moderate (101-1000 dead) or major (> 1000 dead).

○ The acceptability of the molluscide applications may be assessed through water contact observations, both pre- and post-application, and by in-depth, questionnaire-based interviews with key informants from the local community.

### A. Snail population studies

- Snails are collected with three broadly different objectives and on three levels of refinement as to technique depending upon the objectives of the collector.

1) *General survey.* First snails are collected with the objective of determining their distribution to discover all species that may be vectors and to learn the types of habitats frequented by the snails. Consequently no specific collection

technique need be used, the method usually involves visual search supplemented by the use of sieves depending on the nature of the habitat.

2) *Study of population dynamics.* Second snail collections are necessary when an intensive study is made of such things as snail population dynamics life-history growth ecology etc. These studies are in the nature of snail research and are conducted by a specialist. The techniques required are determined by the needs of the researcher but they usually are highly refined and may require exhaustive collections to determine the total snail population in selected areas.

3) *Evaluation of control measures.* Third snail collections are made to measure the efficacy of snail control measures. One of the aims of snail control is reduction of the snail population and a way must be found to measure this change accurately and usefully. For this purpose an accurate measure of the snail population density in the area in which control is to be attempted must be made and then repeated in exactly the same way after the control measure has been put into effect. It must be emphasized that the two measurements must be made in identical fashion must produce data with statistical validity and must present a true picture of the actual snail population at the time they are made.

**B. Prevalence of infection in lower mammals**

○ Prevalence of trematode and especially schistosome infection in lower animals can be an important index of the success of a snail-control program.

○ In areas where *S. japonicum* is endemic the prevalence of this species in lower mammals is of special importance because they can serve as reservoirs of infection transmissible through snails to man. Field-rats lend themselves well in this respect and prevalence in dogs cows and pigs would provide further confirmatory evidence.

○ In areas where *S. mansoni* or S. *haematobium* are endemic changes in the prevalence of other schistosome species in wild or domesticated animals may

give a more rapid indication of a change in transmission by snails than may study of human infections.

**C. Measurement of human prevalence of the disease and of its severity**

- the following kinds of information will be successively required in connection with human schistosome infection:

1) presence or absence of the disease;
2) delimitation of foci of infection;
3) prevalence of the disease (i.e. the number of persons affected by the disease existing in a given population at a given time) ;
4) incidence of the disease (i.e. the number of new cases of the disease within a given population during a stated period of time) ;
5) severity of the disease.

## 6. Special instructions/advice

**A. Protecting the user:**

1) Wear protective clothing and protective gloves when handling the product.
2) Clean hands and face at work intervals and after work.
3) Avoid contact with spilled product or contaminated surfaces.
4) When using, do not eat, drink or smoke.
5) Store niclosamide-containing product containers tightly closed in a cool, well-ventilated place. Store in a place accessible to authorised persons only. Keep product in original packaging.
6) Keep away from direct sun-light. Protect against frost.

**B. First aid measures:**

1) Remove contaminated, soaked clothing immediately and dispose off safely.
2) In case of inhalation, move the patient to fresh air and keep at rest. If symptoms persist call a physician.
3) In case of skin contact, wash off with soap and plenty of water. When symptoms persist or in all cases of doubt seek medical advice.
4) In case of contact with eyes, rinse immediately with plenty of water and seek

medical advice.

5) In case of ingestion, do not induce vomiting. Wash out mouth with water. Keep at rest. Obtain medical attention.

### C. For the doctor:

- There is no known antidote. Treatment after appropriate decontamination should be symptomatic and supportive.

### D. Protecting water catchments:

- A buffer zone may have to be respected as a precautionary measure between treated area and water bodies.

### E. Disposal of waste:

- Product waste may be taken to waste disposal site or incineration plant after consultation with site operator and/or with the responsible authority.
- Packaging that cannot be cleaned should be disposed of as product waste.

# XII. References

1. Molyneux DH (2007) Control of human parasitic diseases. Academic Press, London, UK
2. Garcia LS (2007) Diagnostic medical parasitology. 5th ed., ASM press, Washington D.C.
3. Cable RM (1968) An illustrated laboratory manual of parasitology. 4th ed., Burgess Publishing Company, Minneapolis, Minn
4. John DT, Petri Jr WA (2006) Markell and Voge's medical parasitology. 9th ed., Saunders Elsevier, St. Louis Missouri.
5. Montresor A, Crompton DW, Hall A, Bundy DAP, Saioli L (1998) Guidelines for the evaluation of soil-transmitted helminthiasis and schistosomiasis at community level. WHO/CTD
6. Ministry of education, science and technology, Ministry of health, Kenya. National worm control in school-age children, teacher training kit.
7. Fenwick A, Stothard JR, Thompson H, Magnussen P, Allen H, Engels D, Montresor A, Mubia L (2004) Uganda national bilharzias and worm control programme. Manual district officials and training of trainers.
8. WHO (2006) Prevention and chemotherapy in human helminthiasis. Coordinated use of antheminthic drugs in control interventions: a manual for health professionals and programme managers
9. Montresor A, Gyorkos TW, Crompton DWT, Bundy DAP, Saioli L (1999) Guidelines for monitoring the impact of control programmes aimed at reducing morbidity caused by soil-transmitted helminths and schistosomes, with particular reference to school-age children. WHO/CTD
10. Gyorkos TW (2003) Monitoring and evaluation of large scale helminth control programmes. Acta Trop 86(2-3):275-82
11. Utzinger J, Raso G, Brooker S, De Savigny D, Tanner M, Ornbjerg N, Singer BH, N'goran EK (2009) Schistosomiasis and neglected tropical diseases: towards integrated and sustainable control and a word of caution. Parasitology 136(13):1859-1874.
12. Takougang I, Meli J, Wabo Poné J, Angwafo F 3rd (2007) Community acceptability of the use of low-dose niclosamide (Bayluscide), as a molluscicide in the control of human schistosomiasis in Sahelian Cameroon. Ann Trop Med Parasitol 101(6):479-86.

13. Fenwick A (1970) The development of snail control methods on an irrigated sugar-cane estate in northern Tanzania. Bull World Health Organ 42(4):589-96.
14. Takougang I, Meli J, Angwafo F 3rd. Field trials of low dose Bayluscide on snail hosts of schistosome and selected non-target organisms in sahelian Cameroon. Mem Inst Oswaldo Cruz 101(4):355-8
15. World Health Organozation (1965) Snail control in the prevention of bilharziasis. World Health Organization Monograph Series No. 50.
